# Network analysis of body-related complaints in patients with neurotic or personality disorders referred to psychotherapy

**DOI:** 10.1016/j.heliyon.2023.e14078

**Published:** 2023-02-24

**Authors:** Katarzyna Klasa, Jerzy A. Sobański, Edyta Dembińska, Anna Citkowska-Kisielewska, Michał Mielimąka, Krzysztof Rutkowski

**Affiliations:** Faculty of Medicine, Department of Psychotherapy, Jagiellonian University Medical College, Poland

**Keywords:** Centrality, Bootstrapping, Network analysis, TMFG networks, Anxiety, Somatic symptoms, Psychotherapy patients

## Abstract

**Background:**

Psychopathology theory and clinical practice require the most complex knowledge about patients’ complaints. In patients seeking for psychotherapy, body-related symptoms often complicate treatment.

**Aim:**

This study aimed at examining connections between body-related symptoms, and identification of symptoms which may be responsible for emergency and sustaining of anxiety, somatoform and personality disorders with the use of network analysis.

**Methods:**

In our retrospective research we used data from a sample of 4616 patients of the Department of Psychotherapy, University Hospital in Cracow, diagnosed with anxiety, somatoform or personality disorders. We constructed the Triangulated Maximally Filtered Graph (TMFG) networks of 44 somatoform symptoms endorsed in the symptom checklist “O” (SCL-O) and identified the most central symptoms within the network for all patients and in subgroups of women vs. men, older vs. younger, and diagnosed in 1980–2000 vs. 2000–2015. We used bootstrap to determine the accuracy and stability of five networks’ parameters: strength, expected influence, eigenvector, bridge strength and hybrid centrality.

**Results:**

The most central symptoms within the overall network, and in six subnetworks were dyspnea and migratory pains. We identified some gender-related differences, but no differences were observed for the age and time of diagnosis.

**Conclusions:**

Self-reported dyspnea and migratory pains are potential important targets for treatment procedures.

## Highlights

• Network analysis of 44 body-related symptoms in a large clinical group.
• Subgroups: women/men, older/younger, diagnosed in 1980–2000 vs. 2000–2015.
• Symptom checklist based bootstrapped TMFG networks.
• Dyspnea and migratory pains always the most central symptoms.
• Reminder of controlled breathing and relaxation techniques in psychotherapy.

## 1 Introduction

Body-related symptoms are present in different extents in different mental disorders, complicating the process of diagnosis and treatment [1,2]. It is a known clinical fact, reflected by diversity of diagnostic terms describing patients with somatoform and conversive disorders, “functional” syndromes and symptoms or “medically unexplained disorders” [3], as well as postulated need for more research on, among others, identification and exploration of factors moderating the clinical course and treatment outcomes, understanding how psychological and somatic symptoms develop from somatic conditions and biological and behavioral pathogenic factors, or ways to personalize treatment [4].

Network analysis is a relatively novel approach in psychopathology research [5]. It assumes that symptoms can cause other symptoms, creating specific constellations. In some circumstances, such constellations of interconnected symptoms are maintained by feedback loops, resulting in a condition which may be described as a disorder, without assuming any underlying “cause” (e.g. latent factor) like in traditional approach to psychopathology [[5], [6], [7]]. As methodology of network analysis is constantly being developed [8], results of network analyses are typically visualized as graphs constituted by “nodes” (symptoms or other elements of mental states), with links between nodes called “edges” (reflecting connections, e.g. partial correlations). Implementation of network analysis (NA) in the field of psychopathology provides a novel way of understanding the role of multiple co-occurring symptoms in diagnosing, planning and conducting treatments [9]. In a review published in 2019, there were 363 articles from 2008 to 2018 including 98 theoretical, 61 methodological and 204 empirical. Regarding empirical studies, the most studied disorders were depression and posttraumatic stress disorder (PTSD) [6]. In 2019 Contreras et al. [10] published a systematic review of empirical studies applying NA in psychopathology published between 2010 and 2017. They identified 13 studies on anxiety disorders, 19 on mood disorders, 7 on psychosis, 1 on substance abuse, 1 on borderline personality, 18 on the association of symptoms between disorders and 6 referring to children and adolescents. Those numbers, even taking into consideration that using NA in psychopathology is a relatively new idea, indicate that there is still a space for research.

Most studies on psychopathology are based on cross-sectional between-subjects data, and networks based on within-person variation (longitudinal) gain importance, yet not all refer to clinical populations. As mentioned above, the most studied topics up to date were depression and posttraumatic stress disorder. Summary of existing literature exceeds the scope of our study but as Robinaugh et al. [6] pointed, an example of interesting finding was that the most consistently central depression symptom seemed to be low energy/fatigue and not the cardinal symptoms of depression. At the same time, in a recent narrative review of as many as 56 network studies regarding depression, Wichers et al. [11] stated that there is a need of more overviews of findings of empirical network studies and that their comparison is complicated due to different methodological approches, e.g. different centrality measures applied.

To our best knowledge, there is almost no data regarding body-related (somatic) symptoms networks in large groups of clinical patients diagnosed via face-to-face interview conducted by a psychiatrist. It is even more true for patients suffering from neurotic (in terms of ICD-10) and/or personality disorders. One example of such research is study by Senger et al. [12] who examined 47 physical symptoms listed in DSM-IV in two clinical groups. The first group consisted of 254 outpatients of “psychological institutions” with at least 3 distressing somatic symptoms, at least one of three psychological factors of DSM-5 Somatic Symptom Disorder and a duration of symptoms of at least 6 months. The second group comprised 574 inpatients treated in a psychosomatic hospital suffering from a somatoform disorder. Patients indicated if they have suffered from the listed somatic symptoms in the past 7 days. Senger et al. examined traditional centrality measures, i.e. strength, betweenness and closeness. As for strength they found that nodes (symptoms) with the highest strength were “discomfort in and around precordium” in the first group and “joint pain” in the second which they assessed as more chronic. Kratzer et al. [13] examined 655 adult inpatients treated in the department of psychotraumatology of one of German clinics, to explore connections between PTSD, somatic and dissociative symptoms. All participants were diagnosed with PTSD caused by childhood abuse, nearly 40% were also diagnosed with a personality disorder, similar number with a somatoform disorder, and around 30% with a dissociative disorder (in terms of ICD-10). The authors considered 7 somatic symptoms: “back pains”, “stomach pains or digestive problems”, “feeling of weakness in individual body parts”, “feeling of heaviness in arms and legs”, “pain in your muscles or joints”, “headaches or face pains” and “numbness or tingling in individual body parts”. In the network of all symptoms they found strongly connected symptoms of dissociation, strongly connected somatic symptoms, and relatively strong connections of symptoms within the three dimensions of PTSD, i.e. intrusion, avoidance, hyperarousal. Muscle or joint pain was among the most central symptoms, and headaches or face pains were among symptoms with the strongest connections across the disorder boundaries.

Another example is a study by Bekhuis et al. [14] who examined connections between somatic symptoms and generalized anxiety disorder (GAD) and major depressive disorder (MDD) domains. Their study was the only one referring to somatic symptoms among 56 studies reviewed by Wichers et al. [11]. In 2704 participants from the Netherlands Study of Depression and Anxiety Bekhuis et al. found out that excessive perspiration and pressure/tight feeling in chest were the two somatic symptoms which were associated with the MDD/GAD domains.

Our study aimed at providing more insights about somatic symptoms domain which seem to have been somehow overlooked in empirical studies based on network analysis in clinical populations. We have decided to address this gap in the literature by building a somatoform symptoms network for typical psychotherapy outpatients, i.e., subjects suffering from anxiety, somatoform, conversive disorders, obsessive-compulsive disorder (OCD), with co-occurring personality disorders, and comparing networks between the women and men, older and younger subjects, and patients treated between 1980 and 2000 versus 2000–2015. We compared the overall structure of pairs of networks, how strongly they are connected and including centrality coefficients estimated and bootstrapped for six subsamples.

The remaining sections of the study are; methods, presented in section two, results in section three, discussion is presented in the next section and conclusions are given in the final section of the study.

## 2 Methods

### 2.1 Data

The study was a retrospective one. Data were derived from the pretherapy assessment at a university outpatient psychotherapy department for adult patients with neurotic and personality disorders. To circumvent the issue that participants might be at different stages of intense psychotherapy, we included only patients entering the admission process for the first time. The process of retrospective sample selection (Supplementary Fig. SF1) started with a basic sample of 8173 consecutive patients, diagnosed in the years 1980–2015, seeking psychotherapy. After the first retrospective search, we excluded 1054 records with incomplete data on symptoms, demographic data (procedure allows patient to omit some data) or unclear diagnosis. Records of patients with diagnoses of psychotic or affective disorders, substance abuse, developmental or nonpsychiatric disorders were excluded. Next, we analyzed 7119 identifiable, complete case records and deselected 1263 cases with ICD-10 codes of dysthymia (F34), stress-related disorders (F43), eating disorders (F50) or still unclear diagnoses. Lastly, we implemented the cut-off criterion of symptom checklist SCL-O (GSL index); thus, another 1240 records were removed.

### 2.2 Subjects

A total of 4616 outpatients suffering from various neurotic and/or personality problems with a symptom level score above cut-off points comprised the final sample. Diagnoses were established according to ICD-10 classification since the late 1990s by psychiatrists; earlier case records were reassessed by two principal investigators in terms of ICD-10 chapter F4x and F6x general categories (Table 1). The sample included 3013 (65%) females and 1603 (35%) males. Age ranged from 18 to 62 years (M = 31.8 years; SD = 8.7), all the participants were White-Europeans. More than 40% were married and more than 70% had more than 12 years of education. GSL ranged from 166 to 909 (M = 400; SD = 134; Me = 383) (Table 1). The most frequently endorsed body-related symptoms were constant fatigue and fatigue in the morning, both with a prevalence around 90% (Supplementary Table ST2).

**Table 1:** Sample characteristics (n = 4616).

Demographics				
Gender	3013 females (65%), 1603 males (35%)			
Mean age (SD), years	31.8 (8.7)			
Marital status, married	42.7%			
Above 12 years of education	71.2%			
Mean GSL score, (SD), median	400 (134), 383			
**Diagnosis**				
ICD-10	DSM-IV	DSM-5	N	%
Personality disorders F60/61	301.x	301.x	1553	33.6
Anxiety disorders F40/41	300.0×, 300.2×, 300.3	300.0×, 300.2×, 300.3	1361	29.5
Obsessive-compulsive disorder F42			150	3.3
Somatoform, conversive disorders F44/45	300.1×, 300.6, 300.7, 300.8×	300.1×, 300.6, 300.7, 300.8×	1179	25.5
Neurasthenia F48			373	8.1
Total			4616	100

Patients from a quite long time-period and with a wide spectrum of disorders were selected for this study for three reasons. First, the population was qualified for the same psychotherapeutic day-hospital facility. Second, diagnostic procedures were relatively stable, including the symptom checklist. Third, because network analyses are considered powered enough if there are several subjects per a node, we needed larger sample size as well as subsamples’ sizes to maintain statistical power and reliability of the results.

Patients were assessed twice by a psychiatrist and by a psychologist. After the first interview, patients completed the SCL-O checklist [15].

### 2.3 Ethical considerations

All participants gave written informed consent, and the retrospective research using de-identified data was approved by the Bioethical Committee of the Jagiellonian University (Institutional Review Board, approval number 122.6120.80.2015). The research was performed in accordance with the Declaration of Helsinki.

### 2.4 Inventory

Symptoms were measured with the commonly used in Poland symptom checklist SCL-O, a 138-item self-report questionnaire formulated in a common language [[16], [17], [18], [19]].

SCL-O assesses, among others, the following types of body-related symptoms: somatoform, hypochondriac, and conversive. Severity of distress caused by each symptom in the past week is assessed on a four-point Likert scale. The SCL-O contains no skip-structures. Its test–retest reliability was satisfactory (0.92) [17].

In order to avoid topological overlap between variables [20], items for the network were selected by a psychiatrist and a psychologist, working together to include only items that reflect unique body-related symptoms. Selected items were also checked with the *goldbricker* function in the networktools package [21], with no recommendation to remove of any item. Selected 44 variables were binarized. Reliability of the new scale containing 44 binarized items was good (Cronbach's alpha 0.895; Guttman split-half 0.894). No data were imputed or removed except of missing data mentioned above (Supplementary Fig. SF1). Main assumptions regarding our data include independent cases and that missing data are missing completely at random. We did not remove outliers.

### 2.5 Statistical packages

The analyses have been conducted using R Studio March 1, 1093. For network estimation and visualization, we used the packages *bootnet* [22] and *qgraph* (Epskamp et al., 2012). Layout of the networks was set with a pre-defined algorithm (“Spring”) or *averageLayout* instruction. To assess differences and accuracy of the edges and centralities, we conducted nonparametric bootstrapping with 20,000 samples. Case-dropping bootstrap (nBoots = 20,000) was applied for calculating the Correlation-Stability (CS) coefficient [22]. The *NetworkComparisonTest* package was used to determine whether the compared networks were significantly different. Distributions of edge weights were estimated with *conn* and *edgerep* commands of the *Network Toolbox* [23].

#### 2.5.1 Network building method

We selected the triangulated maximally filtered graph (TMFG) [24], being a variant of the Information Filtering Network (IFN) approach, recommended by Christensen et al. [25] as producing more consistent results for both global and local network characteristics. The TMFG method filters the network by maintaining planarity, retaining fixed number of edges, and generating the so-called chordal network. The advantages are that TMFGs perfectly represent assumptions of Markov and Bayesian networks and use zero-order correlations, which means that the reliability of network measures between samples should be greater. Thus, the TMFG approach protects comparability between our cross-sectional clinical subsamples, because the number of edges is constant and does not vary with sample size, so network measures should be less biased. Another advantage of the TMFG approach is the development of a hierarchical structure, which complements the structure of psychopathological phenomena forming a conceptual hierarchy of dimensions or scales, from local connections, i.e., items. A limitation is that TMFG may add some unnecessary edges (needed in some cliques) [25].

#### 2.5.2 Centrality indices

Because the assumptions and properties of closeness and betweenness (e.g., an effect can only follow one path at a time; and always travel the shortest path or strongest association) do not necessarily characterize networks of psychiatric symptoms, we have omitted these popular centralities. To quantify how well a node is directly connected to other nodes in the network structure, we investigated as centrality measures: *strength*, *expected influence*, *eigenvector*, *bridge strength* and *hybrid centrality*. *Strength* is the sum of the absolute value of all of a node's edges. For the sake of accuracy when some associations may be negative, we include a new measure of node centrality, i.e., expected influence, considering also the sign of associations [7]. *Expected influence* assesses a node's influence with its neighbors (i.e., the nodes with which it shares an edge), and equals to the summed weight, including positive and negative values. Network simulations suggest that expected influence better predicts observed node influence than do other centrality indices not distinguishing positive and negative edges [7,26,27]. *Eigenvector centrality (EC)* is an index of the quality of connections for each neighboring node, considering the transitive importance of nodes with many important neighbors. Thus, higher EC values are given to nodes that have connections to other central nodes [25,28,29]. *Bridge strength* (the sum of the absolute value of all edges that exist between a node A and all nodes that are not in the same community as node A) is calculated to identify node(s) important in communication between communities. The fifth measure, *the hybrid centrality*, ranks nodes on the basis of their centrality values across several other measures of centrality [25,30], and describes highly central nodes with large values and highly peripheral nodes with small values. Hybrid centrality is expected to provide better singular and continuous measure of overall centrality.

#### 2.5.3 Subgroup comparisons

We compared symptom networks of women/men, older/younger, and patients diagnosed between 1980 and 2000 versus 2000–2015 (descriptive statistics are presented in Supplementary Table ST1). *The network comparison test* (NCT) was used to determine whether the subsamples' networks were significantly different. The NCT calculates both network invariance (i.e., significant differences in the structure of the networks) and global strength invariance (i.e., significant differences in the sum of the strength of all of the edges of the networks) [31]. We used NCT with 1000 iterations. We correlated the weighted adjacency matrices and centralities of each pair of compared networks as an additional measure of similarity. Correlations between edges’ weights illustrate similarity of the networks in edges locations (i.e. connections between symptoms/nodes). Correlations of nodes centralities reflect similarities between importance of nodes (symptoms) and seem to add another bit of information regarding network similarity/dissimilarity. We used also correlations for bootstrapped edges and nodes to explore also similarities of pairs of bootstrapped (so as if “better estimated”) networks. The most similar networks should have all abovementioned parameters highly and significantly correlated. Due to a bit different characteristics of Spearman and Kendall tau correlations we presented both of these nonparametric correlations results.

## 3 Results

### 3.1 Sample characteristics

.

### 3.2 Somatoform symptoms network: sample and bootstrap

#### 3.2.1 The full sample: main network structure of somatoform symptoms

**Fig. 1:**
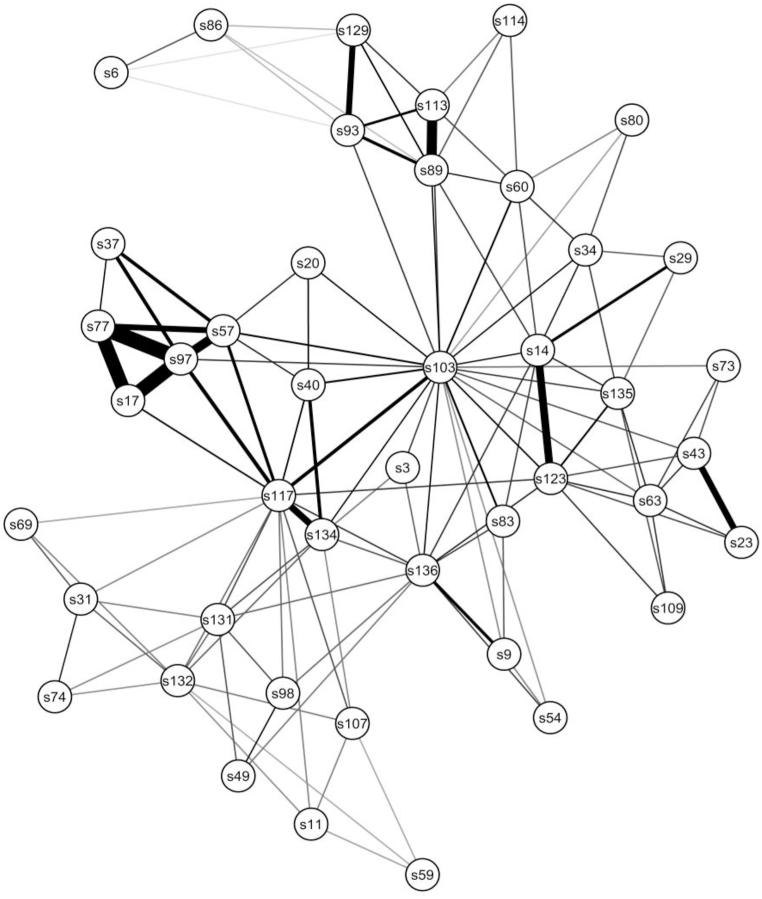
TMFG network structure of somatoform symptoms reported by all patients (n = 4616) at pretherapy assessment.

A graphical depiction of the network of 44 items of somatoform symptoms from the SCL-O checklist in the full sample is presented in Fig. 1 (“Spring” plot), containing item abbreviations, with corresponding symptom descriptions.

Symptoms are represented by nodes and their associations by edges (lines). Thicker edges represent stronger connections (zero-order correlations). s3 indicates choking/“lump”; s6, fatigue in the morning; s9, vomiting in stress; s11, itching or rashes; s14, dizziness; s17, discovering serious diseases; s20, palpitations; s23, loss of sensitivity in parts of the body; s29, persistent headaches; s31, flatulence or involuntary passing of gas; s34, flushes of blood into the head; s37, ritualistic actions to avoid disease; s40, heart pain; s43, temporary paralyses; s49, dry mouth; s54, loss of appetite; s57, focusing on body functions – e.g., pulse; s59, attacks of hunger – e.g., at night; s60, heat or cold w. reasons; s63, periodic blindness or deafness; s69, diarrhea; s73, transient aphonia; s74, constipation; s77, fears about health and contracting diseases; s80, blushing; s83, faintness; s86, constant fatigue; s89, trembling of legs, hands …; s93, muscle cramps; s97, feelings of having serious diseases; s98, excessive thirst; s103, dyspnea; s107, pains in the sexual organs; s109, hypersensitivity; s113, trembling of the face, eyelids, head …; s114, excessive perspiration; s117, undefined “travelling” pains; s123, disorders of balance; s129, muscle tensions; s131, heartburn; s132, passing urine frequently; s134, muscle pains – e.g., in the back; s135, buzzing in the ears; s136, nausea.

#### 3.2.2 Global network properties

The average **connectivity**, measuring the consistency of the edge weights included in the network [32], was 0.27 (SD = 0.09) and **global strength** (sum of absolute edge weights) was 33.55 (sum of bootstrapped means was very similar, 33.84). The edge **density** being the proportion of edges included over the total of all possible connections [25], for any TMFG 44 nodes network is constantly 0.13.

#### 3.2.3 Local network properties

**Sign of connections.** All edges were positive, also in bootstrap results (Supplementary Fig. SF2 and Supplementary Table ST3).

**Fig. 2:**
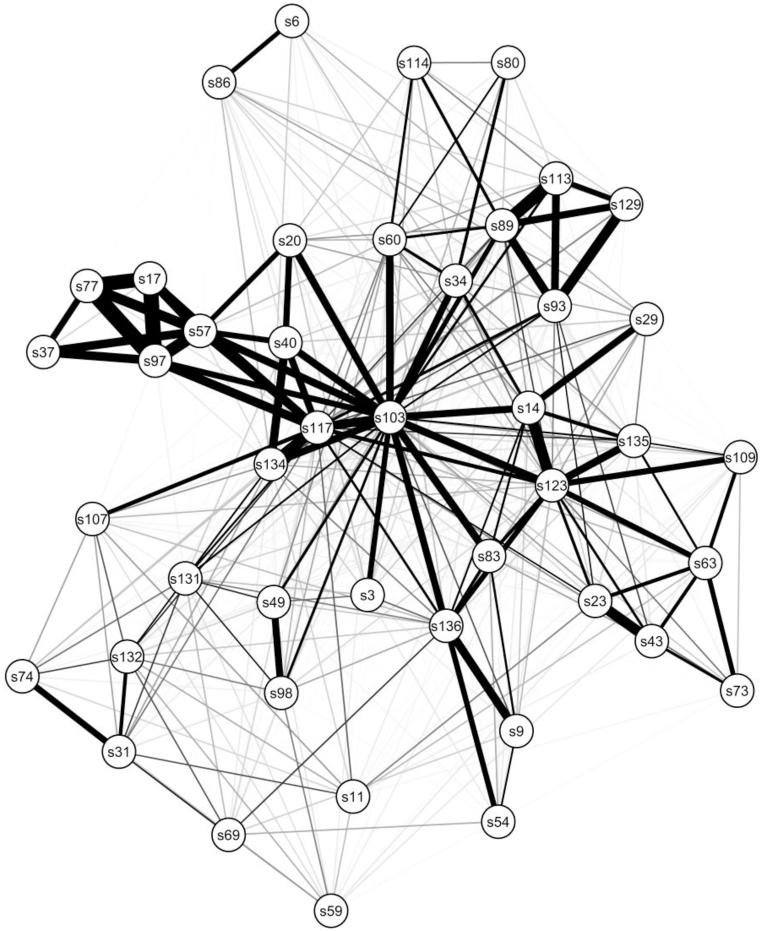
Averaged TMFG network of somatoform symptoms based on mean edges estimated with nonparametric bootstrap analysis (nBoots = 20,000).

**Central symptoms**. As both the sample plot (Fig. 1) and average bootstrap plot (Fig. 2) indicate, the network of 44 somatoform symptoms was organized around two nodes: dyspnea (s103) and migratory pains (s117). The most central node, in terms of all centrality scores, was dyspnea (s103), followed by migratory pains (s117). On the basis of 20,000 bootstrap resamples, the same two items demonstrated significantly greater centralities than almost all other nodes, but were not statistically different from one another (see Supplementary Fig. SF3-4 and Supplementary Table ST4, which present the raw centrality indices for the sample and estimated means for bootstrap with estimated 2.5–97.5 quantiles). All centrality estimates, except bridge strength, were stable (Supplementary Table ST5).

**Subnetworks and clusters**. Despite different item content, five hypochondria symptoms (s17, s37, s57, s77, s97) were strongly interconnected and were placed within a cluster, attached to migratory pains (s117). Another distinct cluster of symptoms was composed of four musculoskeletal symptoms (s89, s93, s113, s129).

**Central hubs**. Bridge strength centrality estimator indicated dyspnea (s103) and migratory pains (s117) as the most central and bridging other symptoms (Supplementary Fig. SF3 and Supplementary Table ST4).

**Results of accuracy and stability checks**. In general, most of the interquantile (2.5–97.5) 95% intervals estimated for weaker edges and lower centrality indices were overlapping (Supplementary Fig. SF3-4 and Supplementary Tables ST3-4). The order of edge/centrality estimates should therefore be interpreted with caution, with the exception of those with the highest scores. Thus, we decided to focus on centrality order, with special regard to the abovementioned significantly higher scores.

Stable centrality estimators indicate high replicability and generalizability of our networks. Of note, CS coefficients above 0.25 indicate moderate, and above 0.50 indicate strong stability [33]. For the main network and all six subnetworks, four of five centrality stability estimates (except bridge strength) were always good to excellent, i.e., CS ranged between 0.680 and 0.880, indicating that above 68% of the data could be dropped to retain with 95% certainty a correlation of 0.70 with the original dataset. Estimated stability of edges was also good (0.440–0.686), except ‘enough’ edge stability of the men subnetwork (0.314) (Supplementary Table ST5). Thus, the case-dropping subset bootstrap procedure showed that the analyzed networks remained stable even after dropping very large proportions of the samples.

### 3.3 Comparisons: gender, age, global symptom level, and date of visit

Local and global network characteristics were compared by the number of edges that replicated between both samples' networks, the differences in the strength of the replicated edge weights, and the strength of rank-order centrality correlations [25,[34], [35], [36], [37], [38]]. We present in the supplementary materials networks’ paired sample plots (Supplementary Fig. SF5, SF9, SF13) with both a fixed (by *averageLayout*) and non-fixed layout because equalized layouts may suggest that network structures are more similar than they actually are. Network *“Spring”* plots placed in the Supplementary File are enriched with average *bootnet* plots based on a large bootstrap (nBoots = 20,000) to underline “probabilistic tendency” illustrated by means of bootnet sampled edges (analogically to Fig. 1, Fig. 2).

#### 3.3.1 Comparison 1. The networks of women and men

Comparing networks between females (n = 3013) and males (n = 1603) yields some significant differences both in the distribution of edge weights (Table 2), and the maximum difference in edges. Further, women's somatoform symptoms network demonstrated significantly lower global strength than men's network. Plots are compared in Supplementary Figure SF5. We then examined centrality indices (Supplementary Fig. SF6-8, Supplementary Table ST7), and we found that in both women and men, the items with the highest centralities were s103 (dyspnea) and s117 (migratory pains). After bootstrapping the networks, there was more evidence that the same two nodes demonstrated the highest centralities. Both networks had similar most and least central nodes, as well as the strongest edges (Supplementary Table ST6). The adjacency matrices (edges' weights) showed strong correlations (both for sample and bootstrapped edges' weights; see Table 2), indicating that the overall structure was quite similar. The similarity was also strong when correlations of centralities were considered (Table 2).

**Table 2:** Summary of global and local characteristics for TMFG networks of 44 somatoform symptom nodes across all pairs of subsamples.

	Comparison 1		Comparison 2		Comparison 3	
	Women n = 3013	Men n = 1603	Older n = 2308	Younger n = 2308	1980–2000 n = 2308	2000–2015 n = 2308
**EDGES**						
No. of edges^a^	126	126	126	126	126	126
Edge density^a^	0.13	0.13	0.13	0.13	0.13	0.13
Connectivity (SD)	0.26 (0.08)	0.29 (0.09)	0.27 (0.09)	0.27 (0.08)	0.26 (0.08)	0.27 (0.08)
Global strength	32.18	36.06	33.37	33.68	32.68	33.92
Mean bootstrap global strength^b^	32.64	36.74	33.72	34.09	33.18	34.22
NCT Global strength invariance test S and p value	3.88, p < 0.01		0.31, p > 0.70		1.24, p > 0.15	
NCT Network invariance test M (max. Difference in edge weights) and p value	0.39, p < 0.005		0.31, p > 0.95		0.34, p > 0.35	
No. of replicated (non-zero) edges^c^ (%)	71 (56%)		80 (64%)		77 (61%)	
No. of non-zero bootstrap edge weight means (%)	766 (81%)		769 (81%)		767 (81%)	
Mean difference of edges (SD)	0.040 (0.027)		0.027 (0.018)		0.031 (0.028)	
**Correlations between non-zero replicated edges' weights (s), and correlations between non-zero edge weights bootstrap means (b)**						
Spearman R	R_s_ = 0.88 R_b_ = 0.72		R_s_ = 0.88 R_b_ = 0.80		R_s_ = 0.77 R_b_ = 0.78	
Kendall tau	τ_s_ = 0.71 τ_b_ = 0.54		τ_s_ = 0.70 τ_b_ = 0.61		τ_s_ = 0.61 τ_b_ = 0.58	
**Correlation between all edges**						
Spearman R	R_s_ = 0.54 R_b_ = 0.80		R_s_ = 0.62 R_b_ = 0.86		R_s_ = 0.60 R_b_ = 0.83	
Kendall tau	τ_s_ = 0.52 τ_b_ = 0.62		τ_s_ = 0.60 r_b_ = 0.68		τ_s_ = 0.57 τ_b_ = 0.66	
**NODE CENTRALITY CORRELATIONS**						
**Strength**						
Spearman R	R_s_ = 0.87 R_b_ = 0.92		R_s_ = 0.83 R_b_ = 0.90		R_s_ = 0.79 R_b_ = 0.89	
Kendall tau	τ_s_ = 0.69 τ_b_ = 0.76		τ_s_ = 0.67 τ_b_ = 0.75		τ_s_ = 0.62 τ_b_ = 0.74	
**Expected influence**						
Spearman R	R_s_ = 0.87 R_b_ = 0.92		R_s_ = 0.83 R_b_ = 0.90		R_s_ = 0.79 R_b_ = 0.89	
Kendall tau	τ_s_ = 0.69 τ_b_ = 0.76		τ_s_ = 0.67 τ_b_ = 0.75		τ_s_ = 0.62 τ_b_ = 0.74	
**Eigenvector centrality**						
Spearman R	R_s_ = 0.82 R_b_ = 0.90		R_s_ = 0.91 R_b_ = 0.95		R_s_ = 0.86 R_b_ = 0.92	
Kendall tau	τ_s_ = 0.62 τ_b_ = 0.72		τ_s_ = 0.75 τ_b_ = 0.82		τ_s_ = 0.69 τ_b_ = 0.78	
**Hybrid**						
Spearman R	R_s_ = 0.80 R_b_ = 0.89		R_s_ = 0.83 R_b_ = 0.90		R_s_ = 0.82 R_b_ = 0.88	
Kendall tau	τ_s_ = 0.61 τ_b_ = 0.71		τ_s_ = 0.67 τ_b_ = 0.74		τ_s_ = 0.67 τ_b_ = 0.73	
**Bridge strength**						
Spearman R	R_s_ = 0.41 R_b_ = 0.83		R_s_ = 0.72 R_b_ = 0.91		R_s_ = 0.32 R_b_ = 0.86	
Kendall tau	τ_s_ = 0.34 τ_b_ = 0.64		τ_s_ = 0.62 τ_b_ = 0.74		τ_s_ = 0.26 τ_b_ = 0.70	
**TWO MOST CENTRAL NODES**^d^						
Criterion: strength	s103, s117	s103, s117	s103, s117	s103, s117	s103, s117	s103, s117
expected influence	s103, s117	s103, s117	s103, s117	s103, s117	s103, s117	s103, s117
eigenvector centrality	s103, s117	s103, s117	s103, s117	s103, s117	s103, s117	s103, s117
bridge strength	s103, s117	s103, s117	s103, s117	s103, s117	s103, s117	s103, s117
hybrid centrality	s103, s117	s103, s117	s103, s117	s103, s117	s103, s117	s103, s117

a Number of edges in TMFG network of 44 nodes is constantly 126 and thus density is constantly 0.133.

b nBoots = 20,000 nonparametric bootstrap.

c Edge densities were equivalent between the TMFG networks, so only one percentage per pair of subsamples was produced.

d Ordered according to bootstrap means ordered. All correlations were statistically significant (p < 0.001).

#### 3.3.2 Comparison 2. The networks of older and younger patients

Comparing networks of older (above median age, i.e., 30 years; n = 2308) and younger patients did not show significant differences neither in distribution of edge weights, nor in network global strength (Table 2). Next, we examined centrality indices (Supplementary Fig. SF10-SF12 and Supplementary Table ST9), and found again that in both age-split networks, the items with the highest node centralities were dyspnea (s103) and migratory pains (s117). Bootstrapping the network confirmed that observation. Both networks had similar strongest edges (Supplementary Table ST8). Supplementary Table ST9 shows similar most central and least central nodes, too. The networks are plotted in Supplementary Figure SF9.

#### 3.3.3 Comparison 3. The networks of patients diagnosed in 1980–2000 and 2000–2015

Plotted graphical depictions of the networks of patients diagnosed in 1980–2000 (split after the 2,308th patient in the midst of the year 2000) and in 2000–2015 suggested similar patterns of connectivity (Supplementary Fig. SF13). Comparing networks with NCT yields no significant differences (Table 2). In both networks, items with greater node centralities than almost all other items were again dyspnea and migratory pains, and it was confirmed with bootstrap (Supplementary Figs. SF14-16 and Supplementary Table ST11). Edge weights (all edges, replicated non-zero edges, and bootstrapped edge means) were strongly correlated (Table 2), indicating a high degree of similarity. Similarly, the consistency in all five centralities between networks was high (correlations, see Table 2). Therefore, networks of patients diagnosed in 1980–2000 and in 2000–2015 are very similar.

## 4 Discussion

To facilitate a better understanding of what the somatoform component is, we applied network analysis to somatoform symptoms in a heterogeneous sample of psychotherapy outpatients, with the aim to determine the most central somatoform complaints endorsed in that particular clinical context.

We analyzed 44 selected body-related symptoms, including hypochondriac ones, derived from the symptom checklist SCL-O. It was a much longer list of symptoms than in previous studies using other questionnaires, like the *Four-Dimensional Symptom Questionnaire* (4DSQ) [14], containing in the somatization scale 16 somatic symptoms, or PHQ-15 [39], containing 15 symptoms. Items used in our analyses were partly similar, but we hoped to get a more comprehensive picture of possible relations among bodily symptoms reported by patients. It is important to note that it is not possible to compare our results with those by Bekhuis et al. [14] or Mostafaei et al. [39], even taking into consideration that some symptoms, including shortness of breath and muscle pain, were examined in all three studies. Bekhuis et al. and Mostafaei et al. focused on connections between somatic symptoms and other complaints, i.e., GAD and MDD domains [14] and major mental problems and personality traits [39] (moreover, with the use of a bit different methodological approach, i.e. exploratory factor analysis and Bayesian regularized quantile regression with adaptive LASSO penalization, in a non-clinical sample). Recently, Senger et al. [12] published a study on 47 physical symptoms listed in DSM-IV in two groups of patients with somatoform disorders, pointing at symptoms of “discomfort in and around precordium” and “joint pain” as important and suggesting that the results may be generalized to similar population of patients. Interestingly, in the study by Kratzer et al. [13] on PTSD inpatients with comorbid personality, somatoform and dissociative disorders, “muscle or joint pain” was among the most central symptoms within the somatic domain of the network they found. We may only speculate to what extent that complaint is similar to “migratory pains” indicated as central in our study.

Because networks can change substantially depending on symptoms included, future researchers may administer other validated measures of somatoform symptoms, as well as clinician-derived ratings, also given concerns with the current taxonomy of DSM-5 and ICD-11. Considering our findings, we recommend inclusion of items referring to health anxiety (hypochondria).

The SCL-O questionnaire contains the most frequent complaints of patients, and variables included reflect neither one latent variable nor one underlying construct, so distortions of centrality estimates by clustering symptoms in one factor, mentioned by Burger et al. [40], are avoided. Also similar bias of trivial confirmation of centrality of symptoms obligatory to diagnose a particular syndrome according to the ICD/DSM criteria [20,41,42] was avoided due to the fact that SCL-O is independent of those classifications. Our study is based on a large clinical sample while sample size in many psychopathological network studies is around hundreds [10,12], rarely achieving dozens of thousands [43]. The existing research rarely considers gender and age of patients, possibly due to limited numbers, and we have not encountered studies comparing networks evaluated in different years/decades.

We identified items assessing dyspnea and migratory pains as most central in the somatoform network. Quite surprisingly, tachycardia and heart pain (despite of being frequently connected to anxiety) appeared not as central as we expected, but centrality is also strongly determined by the estimated network. A possible explanation may be connected with the fact that our sample consisted of patients referred to psychotherapy, thus aware that their cardiac symptoms were not dangerous.

Prior studies identified core depressive, PTSD, anxiety, and other symptoms as central, predictive, bridging, and strongly interrelating each other, in mostly non-clinical populations. The current research shows that dyspnea and migratory pains are particularly important, central hubs maintaining somatoform symptoms networks, whereas cardiac or gastrointestinal complaints are not. However, the finding of Olatunji et al. [44] suggests that the symptoms that are most central may not necessarily be reliable predictors of the level of dysfunction.

To address potential gender differences, we compared the networks between men and women. We found almost no differences in the centrality estimates, although women exhibited lower global network strength and connectivity. This suggests that, while women may have greater levels of somatoform symptomatology [[45], [46], [47]], the associations among complaints are not so much different in men and women. As for potential differences regarding somatic symptoms connected with the age of participants [48,49], we also may conclude that NA showed none, but our sample was relatively young.

Complaints of dyspnea and migratory pains had the highest centrality in the networks of all analyzed symptoms, regardless of age and gender. Comparison of the two long time-periods of 20 vs. 15 years showed no differences (despite some variations year-to-year reported elsewhere), because the centrality is evidenced as being caused more by the interaction of all symptoms in the network than by their frequency. Of note, we analyzed data from before the current pandemic, so we cannot attribute it to its potential influence. One plausible explanation is that dyspnea strongly indicates dysregulation of the autonomic system, omnipresent in neurotic disorders. A second possible explanation is that in contrast to many “understandable” bodily symptoms (e.g., gastrointestinal attributed commonly to intoxication), both dyspnea and migratory pains may be less “understandable” for patients, thus more anxiety provoking and/or loaded with unconscious symbolic meaning (like being choked/controlled). Of course the binarization of our data, by neglecting symptoms intensity may be responsible for some blur, and that issue requires further investigation. Still, our findings provide additional light on connections among somatoform symptoms in typical psychotherapy outpatients.

### 4.1 Clinical implications

As frequently proposed [10], the most central nodes may serve as a vehicle for streamlined treatments targeting symptoms sustaining the rest of the disorder. The suggestion that dyspnea may drive the rest of the somatoform symptoms is clinically interesting, as it may be addressed by several interventions/breathing techniques targeting that symptom [[50], [51], [52], [53], [54], [55], [56]]. The next central symptom of migratory pains as well as all psychogenic muscular cramps and tensions may be addressed through another set of well-established techniques of progressive muscular relaxation, autogenic training or yoga [[57], [58], [59], [60]]. Finally, one could expect that any successful treatment oriented on dyspnea and migratory pains should robustly affect somatoform symptoms, profoundly changing the structure of the network.

## 5 Conclusions

Overall, we conclude that there is much similarity in the pattern of centrality across six subgroups and the whole sample. Integrating all the observations with five centrality estimators and the results of seven bootstrap analyses, we conclude that the two most central nodes of dyspnea and migratory pains were significantly different from most of the other items, meaning they were more important. The abovementioned features of nodes are very convergent for various estimators and in subsamples analyzed as well as confirmed with bootstrap analyses. Because comparing three pairs of networks yielded limited differences, we assume that somatoform symptoms are building similar networks in patients regardless of age, time-period of data collection, and to some extent, gender.

As far as we know, no authors up to date reported a network of somatoform symptoms and its centralities, with regard to split subpopulations. Also, to the best of our knowledge, this is the first study examining the psychopathological networks of somatoform symptoms in a large group of patients referred to psychotherapy. Obviously, replication studies using similar methodology are needed before drawing any firm conclusion. Despite limitations, our research supports the interconnected network structure of body-related symptoms.

Finally, we believe that our study may contribute to the ongoing vivid discussion of networks’ reproducibility and applicability [[34], [35], [36], [37], [38]].

Recommendations: Our findings point at specific symptoms of dyspnea and migratory pains as potential targets for treatment procedures.

### 5.1 Limitations

Our study has several limitations. It employed a cross-sectional approach, so analyses were restricted to the group-level and to a single time-point (qualification to psychotherapy). The study was exploratory and retrospective, not experimental. Accordingly, definitive causal claims cannot be made about how symptoms in the network related to each other. We used the set of symptoms derived from one self-report instrument. Our instrument, similarly to many questionnaires (e.g., the 4DSQ or SOMS-7T), collects data on the previous week and omits the long-term persistence of symptoms. The sample, despite its size, was based on one site only and included only White-European participants. The inclusion of individuals with a GSL above cut-off point and not restricting inclusion to somatoform disorders may also be considered limitations. However, including subjects with a too limited number of symptoms could alter the structure of the network significantly. Excluding patients with diagnoses other than somatoform limits conclusions to a subgroup reporting predominantly somatoform symptoms and omits those with somatoform complaints co-occurring with other. We are also aware of including not many subjects older than 50 due to the characteristics of our department. Other factors that may account for potential bias in our results include a high prevalence of university students and females, nevertheless reflecting our clinical practice. Finally, although in our opinion the TMFG method performed well, i.e., reflected clinically known and important connections, the three- and four-clique structure imposed by that method might not be suitable for all types of psychopathological networks. Moreover, the TMFG-derived specific chordal network structure could include too few (allowing only edges that keep the network a tree or planar) or too many (some spurious edges may be necessary to maintain chordality) connections, than what are in the “true network” [25]. It is also important to note that the direction of evidenced associations is unclear as the presented network is an undirected graph.

## Author contribution statement

Katarzyna Klasa: Conceived and designed the experiments; Performed the experiments; Analyzed and interpreted the data; Wrote the paper.

Jerzy A. Sobański: Conceived and designed the experiments; Performed the experiments; Contributed reagents, materials, analysis tools or data; Analyzed and interpreted the data; Wrote the paper.

Edyta Dembińska; Anna Citkowska-Kisielewska; Michał Mielimąka: Analyzed and interpreted the data; Wrote the paper.

Krzysztof Rutkowski: Contributed reagents, materials, analysis tools or data; Analyzed and interpreted the data; Wrote the paper.

## Funding statement

This work was supported by 10.13039/100009045Uniwersytet Jagielloński Collegium Medicum, Wydział Lekarski [K/ZDS/000422 and 501/NKL/270/L].

## Data availability statement

The data that support the findings of this study are not publicly available due to their containing information that could compromise the privacy of research participants but are available from JAS upon reasonable request.

## Declaration of interest's statement

The authors declare no competing interests.
