# Supplementary material for: Network analysis of body-related complaints in patients with neurotic or personality disorders referred to psychotherapy
Source: Heliyon. 2023 Feb 24;9(3):e14078. doi: 10.1016/j.heliyon.2023.e14078 (PMC10018473; doi:10.1016/j.heliyon.2023.e14078)
Supplement: Multimedia component 1 [file mmc1.pdf]

SUPPLEMENTARY MATERIAL FILE

Network analysis of body-related complaints in patients with neurotic or personality disorders referred to psychotherapy

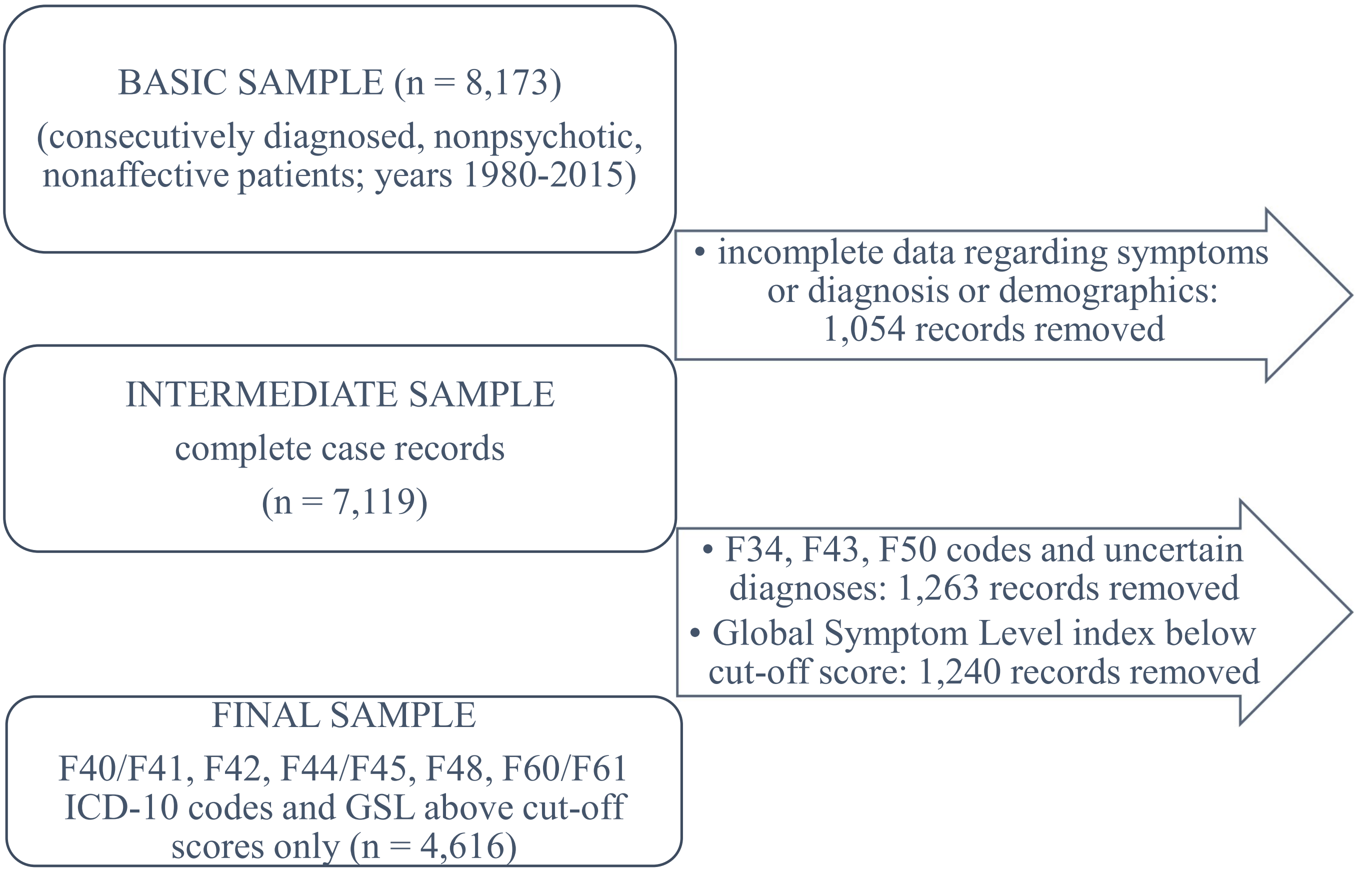

Supplementary Fig. SF1. Flowchart illustrating the process of case records selection.

Supplementary Table ST1. Age and Global Symptom Level in the final sample and subsamples

		Age [years]					Global Symptom Level (GSL)				
	**N**	**Mean ± SD**	**Min**	**Max**	**Me**	**Q1—Q3**	**Mean ± SD**	**Min**	**Max**	**Me**	**Q1—Q3**
**Women**	3,013	32.2 ± 8.7	18.0	62.0	31.0	24.9—38.0	414 ± 133	201	909	400	311—501
**Men**	1,603	30.9 ± 8.4	18.9	62.0	28.0	23.0—37.0	373 ± 132	166	852	349	269—459
**Older**	2,308	38.8 ± 6.4	29.9	62.0	38.0	33.0—43.0	405 ± 136	169	852	389	297—495
**Younger**	2,308	24.7 ± 3.0	18.0	29.9	23.9	23.0—28.0	395 ± 131	166	909	379	289—478
**1980–2000**	2,308	33.0 ± 8.5	19.0	53.0	33.0	28.0—38.0	408 ± 137	166	869	392	298—502
**2000–2015**	2,308	30.5 ± 8.6	18.0	62.0	28.0	23.9—34.9	392 ± 130	166	909	375	289—475
**Total**	4,616	31.8 ± 8.7	18.0	62.0	29.9	23.9—38.0	400 ± 134	166	909	383	293—487

Mean – arithmetic mean, SD – standard deviation, Min – minimum, Max – maximum, Me – median, Q1—Q3 – range between lower and upper quartile, Older – age above median (i.e., 29.9 years), Younger – age below median, 1980–2000 – first 2,308 patients diagnosed between 1980 and 2000, 2000–2015 – next 2,308 patients diagnosed between 2000 and 2015.

Supplementary Table ST2. Somatoform symptoms endorsements in subgroups

Node	Total	Women	Men	Older	Younger	1980-2000	2000-2015
s3	68.0%	72.5%	59.5%	70.8%	65.2%	68.2%	67.9%
s6	90.1%	91.3%	87.9%	90.6%	89.6%	89.0%	91.3%
s9	20.9%	23.8%	15.6%	23.4%	18.4%	23.8%	18.0%
s11	38.8%	42.2%	32.4%	42.7%	34.9%	41.7%	35.9%
s14	66.8%	71.4%	58.1%	70.2%	63.3%	71.1%	62.4%
s17	65.5%	64.3%	67.7%	68.9%	62.0%	67.9%	63.1%
s20	80.4%	83.0%	75.4%	81.5%	79.2%	84.1%	76.7%
s23	25.9%	27.5%	22.8%	29.9%	21.8%	28.4%	23.4%
s29	64.4%	68.7%	56.2%	67.8%	61.0%	68.0%	60.7%
s31	50.0%	50.7%	48.5%	52.8%	47.1%	47.3%	52.6%
s34	55.3%	55.2%	55.5%	57.5%	53.0%	61.1%	49.4%
s37	51.7%	50.4%	54.0%	54.0%	49.3%	53.2%	50.1%
s40	61.0%	62.7%	57.8%	66.1%	55.9%	68.9%	53.1%
s43	20.0%	21.6%	17.2%	23.6%	16.5%	24.0%	16.1%
s49	61.9%	63.6%	58.9%	65.2%	58.7%	62.0%	61.9%
s54	52.0%	54.0%	48.2%	53.2%	50.7%	55.0%	49.0%
s57	56.4%	54.7%	59.6%	59.1%	53.8%	60.1%	52.8%
s59	33.1%	35.0%	29.4%	32.8%	33.4%	30.9%	35.2%
s60	63.2%	67.5%	55.0%	65.6%	60.8%	67.3%	59.1%
s63	31.0%	32.0%	29.2%	34.6%	27.5%	36.8%	25.3%
s69	35.4%	35.4%	35.4%	34.4%	36.4%	31.4%	39.3%
s73	23.4%	23.4%	23.3%	24.0%	22.7%	28.0%	18.7%
s74	40.0%	46.1%	28.6%	43.5%	36.5%	40.2%	39.8%
s77	68.7%	68.2%	69.6%	71.8%	65.6%	72.1%	65.3%
s80	46.9%	49.9%	41.2%	47.3%	46.4%	47.4%	46.3%
s83	26.9%	30.2%	20.8%	30.8%	23.1%	33.3%	20.5%
s86	89.6%	90.2%	88.5%	90.3%	89.0%	89.5%	89.8%
s89	66.9%	68.7%	63.6%	68.8%	65.0%	73.1%	60.7%
s93	47.4%	48.9%	44.7%	52.0%	42.9%	49.4%	45.5%
s97	51.3%	48.7%	56.3%	54.5%	48.1%	55.2%	47.4%
s98	46.2%	46.2%	46.1%	46.6%	45.8%	50.7%	41.7%
**s103**	**57.8%**	**60.9%**	**52.2%**	**62.5%**	**53.2%**	**64.0%**	**51.7%**
s107	24.1%	27.5%	17.8%	24.0%	24.2%	26.0%	22.2%
s109	46.0%	49.3%	39.9%	50.6%	41.5%	54.2%	37.8%
s113	56.3%	56.9%	55.1%	58.1%	54.4%	61.9%	50.6%
s114	75.4%	76.2%	73.8%	76.9%	73.9%	81.1%	69.7%
**s117**	**47.0%**	**48.9%**	**43.5%**	**53.6%**	**40.5%**	**54.5%**	**39.5%**
s123	48.4%	51.9%	41.6%	50.9%	45.8%	53.8%	42.9%
s129	64.6%	67.3%	59.5%	64.9%	64.3%	66.1%	63.1%
s131	41.8%	40.0%	45.1%	44.8%	38.7%	43.6%	39.9%
s132	48.3%	49.8%	45.5%	51.3%	45.4%	50.4%	46.2%
s134	64.0%	66.0%	60.2%	70.7%	57.4%	69.2%	58.9%
s135	41.9%	42.7%	40.2%	46.6%	37.1%	45.9%	37.8%
s136	48.0%	52.2%	40.0%	48.2%	47.8%	49.8%	46.2%

Nodes/Symptoms: s3 indicates choking/"lump"; s6, fatigue in the morning; s9, vomiting in stress; s11, itching or rashes; s14, dizziness; s17, discovering serious diseases; s20, palpitations; s23, loss of sensitivity in parts of the body; s29, persistent headaches; s31, flatulence or involuntary passing of gas; s34, flushes of blood into the head; s37, ritualistic actions to avoid disease; s40, heart pain; s43, temporary paralyses; s49, dry mouth; s54, loss of appetite; s57, focusing on body functions – e.g., pulse; s59, attacks of hunger – e.g., at night; s60, heat or cold w. reasons; s63, periodic blindness or deafness; s69, diarrhea; s73, transient aphonia; s74, constipation; s77, fears about health and contracting diseases; s80, blushing; s83, faintness; s86, constant fatigue; s89, trembling of legs, hands…; s93, muscle cramps; s97, feelings of having serious diseases; s98, excessive thirst; s103, dyspnea; s107, pains in the sexual organs; s109, hypersensitivity; s113, trembling of the face, eyelids, head…; s114, excessive perspiration; s117, undefined "travelling" pains; s123, disorders of balance; s129, muscle tensions; s131, heartburn; s132, passing urine frequently; s134, muscle pains – e.g., in the back; s135, buzzing in the ears; s136, nausea.

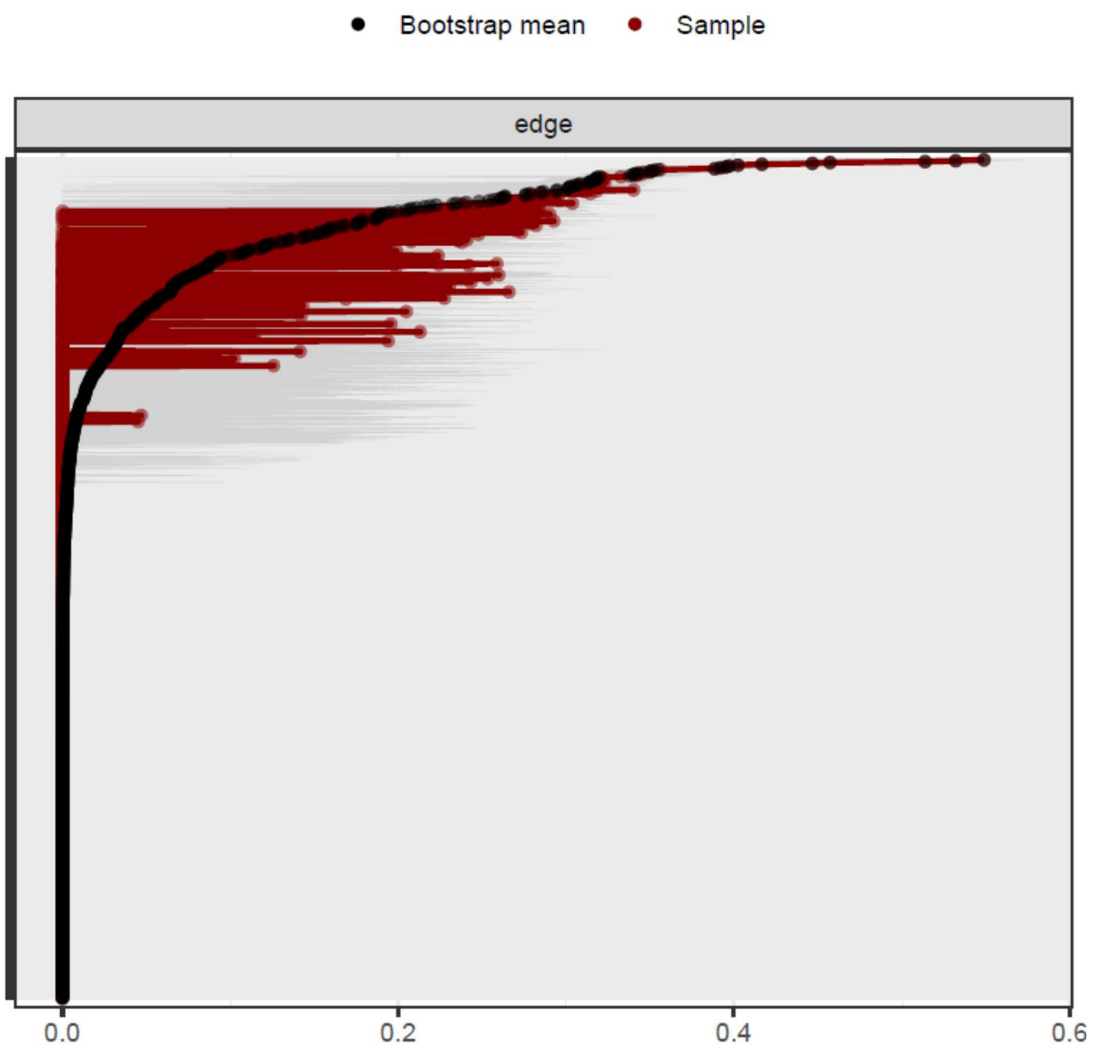

Supplementary Fig. SF2. Accuracy of the edge-weights for the estimated network model. The horizontal area within the plot represents the 95% quantile range of the parameter values across 20,000 bootstraps. The *red line* indicates the sample values and the *gray area* the bootstrapped CIs. Each *horizontal line* represents one edge of the network. The order is set from the strongest to the weakest edge (according to bootstrap means). Network of 44 somatoform symptoms is based on 4,616 patients. The mean of the bootstrap samples (*black line*) was used in ordering the edges. The *y*-axis labels are raw scores, and X-axis labels are removed to avoid cluttering.

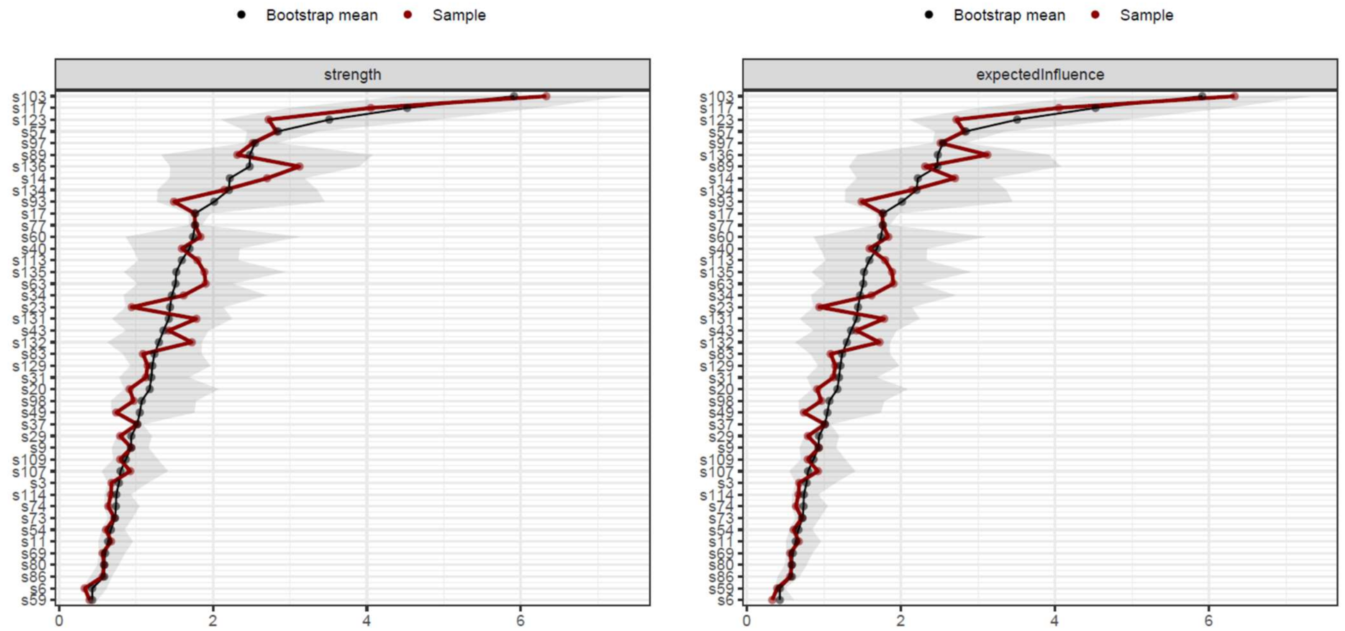

Supplementary Fig. SF3. Bootstrapped (nBoots = 20,000) confidence intervals of estimated node centrality for the network of 44 somatoform symptoms.

UPPER LEFT PANEL: strength
LOWER LEFT PANEL: eigenvector

UPPER RIGHT PANEL: expected influence
LOWER RIGHT PANEL: bridge strength

The *red line* indicates the sample values and the *gray area* the bootstrapped CIs. Each *horizontal line* represents one node of the network. The order is set from the strongest to the weakest node (according to bootstrap means). Network of 44 somatoform symptoms is based on 4,616 patients. The mean of the bootstrap samples (*black line*) was used in ordering the edges. The *y*-axis labels are raw scores.

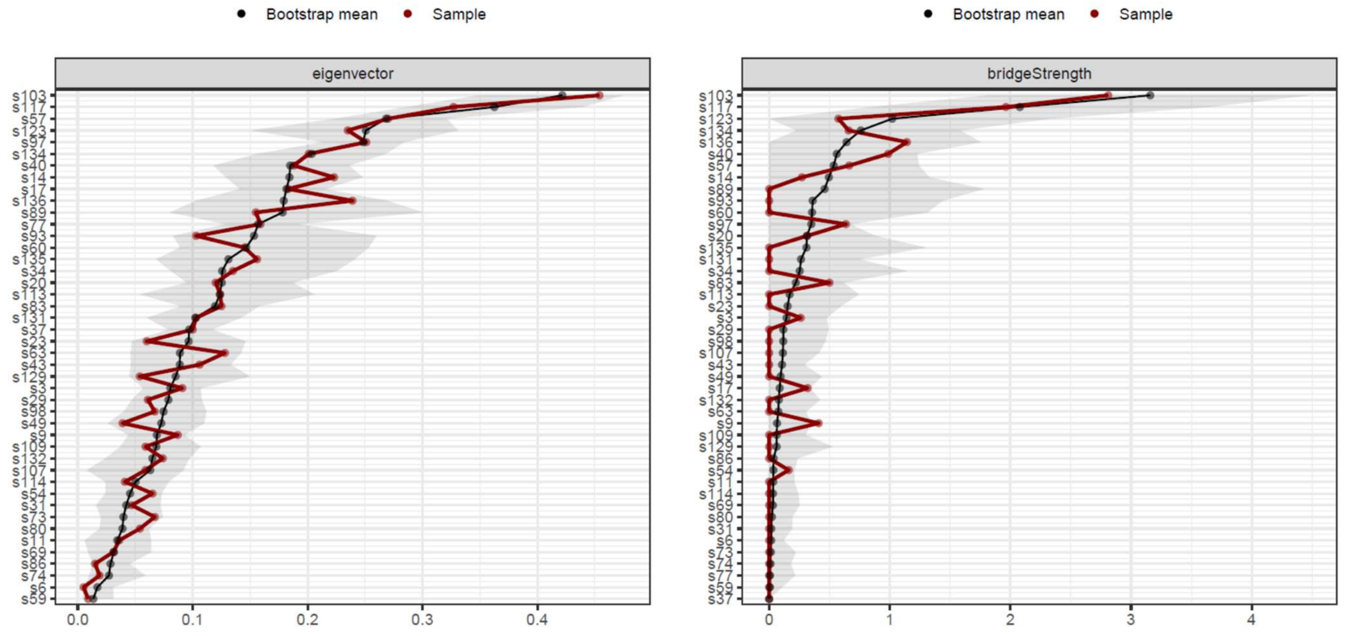

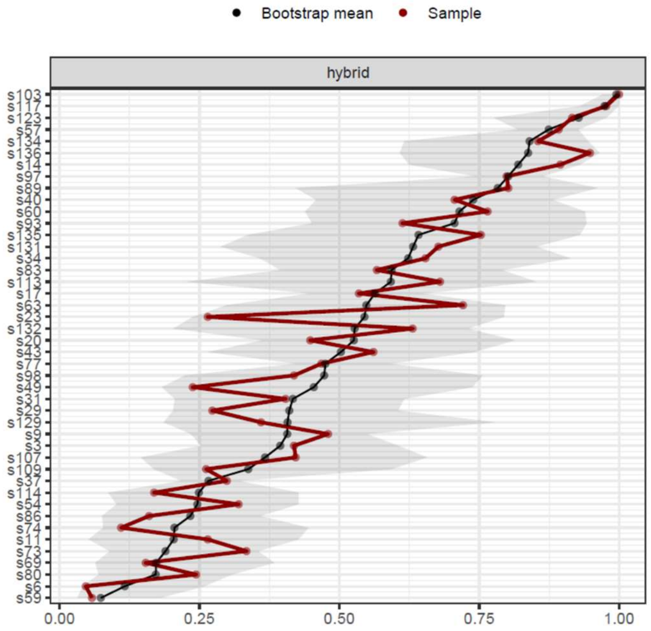

Supplementary Fig. SF4. Bootstrapped (nBoots = 20,000) confidence intervals of estimated node **hybrid** centrality for the network of 44 somatoform symptoms.

The *red line* indicates the sample values and the *gray area* the bootstrapped CIs. Each *horizontal line* represents one node of the network. The order is set from the strongest to the weakest node (according to bootstrap means). Network of 44 somatoform symptoms is based on 4,616 patients. The mean of the bootstrap samples (*black line*) was used in ordering the edges. The *y*-axis labels are raw scores.

Supplementary Table ST3. All patients; main network. Twenty edges in the network with the highest mean weights (raw scores): sample and bootstrap estimates

Edges				
id	Sa	Bo	q2.5	q97.5
s77--s97	0.55	0.55	0.52	0.57
s17--s97	0.53	0.53	0.51	0.56
s17--s77	0.51	0.51	0.48	0.54
s89--s113	0.46	0.46	0.43	0.49
s57--s97	0.45	0.45	0.42	0.48
s14--s123	0.42	0.42	0.39	0.44
s117--s134	0.40	0.40	0.37	0.43
s23--s43	0.40	0.40	0.36	0.43
s17--s57	0.40	0.40	0.37	0.43
s57--s77	0.39	0.39	0.36	0.42
s93--s129	0.39	0.39	0.36	0.42
s97--s117	0.36	0.35	0.32	0.39
s37--s97	0.35	0.35	0.32	0.38
s103--s117	0.35	0.35	0.32	0.38
s37--s57	0.35	0.35	0.32	0.38
s9--s136	0.34	0.34	0.31	0.37
s40--s134	0.35	0.34	0.31	0.38
s89--s93	0.34	0.34	0.31	0.37
s57--s117	0.34	0.34	0.31	0.37
s93--s113	0.33	0.32	0.00	0.36

id – edge identifier (with codes of the two nodes), Sa – score of the sample, Bo – mean of the bootstrap, q2.5 – 2.5th percentile, q97.5 – 97.5th percentile

Supplementary Table ST4. All patients; main network. Sample raw scores and bootstrap estimated means for all five centralities

Strength centrality					Expected influence centrality					Eigenvector centrality					Bridge strength centrality					Hybrid centrality				
Sy	Sa	Bo	q2.5	q97.5	Sy	Sa	Bo	q2.5	q97.5	Sy	Sa	Bo	q2.5	q97.5	Sy	Sa	Bo	q2.5	q97.5	Sy	Sa	Bo	q2.5	q97.5
s103	6.33	5.92	4.44	7.33	s103	6.33	5.92	4.48	7.32	s103	0.45	0.42	0.35	0.48	s103	3.65	3.17	1.81	4.51	s103	1.00	1.00	0.97	1.00
s117	4.05	4.53	3.00	6.24	s117	4.05	4.52	3.00	6.21	s117	0.33	0.36	0.29	0.44	s117	1.64	2.08	0.85	3.66	s117	0.98	0.97	0.93	1.00
s123	2.72	3.50	2.11	4.89	s123	2.72	3.50	2.11	4.87	s57	0.27	0.27	0.23	0.32	s123	0.57	1.03	0.00	2.32	s123	0.92	0.93	0.78	0.98
s57	2.82	2.85	2.45	3.36	s57	2.82	2.84	2.44	3.36	s123	0.24	0.25	0.15	0.33	s134	0.31	0.76	0.23	1.59	s57	0.89	0.87	0.82	0.93
s97	2.52	2.54	2.42	2.83	s97	2.52	2.54	2.42	2.82	s97	0.25	0.25	0.21	0.28	s136	1.14	0.65	0.00	1.76	s134	0.86	0.84	0.62	0.95
s89	2.31	2.48	1.33	4.10	s89	2.31	2.49	1.33	4.07	s134	0.20	0.20	0.16	0.27	s40	0.93	0.56	0.00	1.23	s136	0.95	0.84	0.61	0.97
s136	3.12	2.47	1.44	3.93	s136	3.12	2.48	1.44	3.92	s40	0.19	0.19	0.12	0.24	s57	0.96	0.53	0.00	1.24	s14	0.90	0.82	0.63	0.93
s14	2.70	2.21	1.42	3.17	s14	2.70	2.22	1.43	3.16	s14	0.22	0.18	0.13	0.25	s14	0.54	0.50	0.00	1.33	s97	0.80	0.80	0.74	0.86
s134	2.15	2.21	1.28	3.37	s134	2.15	2.21	1.28	3.34	s17	0.18	0.18	0.14	0.21	s89	0.86	0.46	0.00	1.76	s89	0.80	0.78	0.42	0.96
s93	1.49	2.02	1.27	3.46	s93	1.49	2.01	1.27	3.42	s136	0.24	0.18	0.10	0.27	s93	0.27	0.36	0.00	1.46	s40	0.71	0.74	0.46	0.88
s17	1.76	1.77	1.68	1.97	s17	1.76	1.77	1.68	1.97	s89	0.16	0.18	0.08	0.30	s60	0.78	0.35	0.00	1.32	s60	0.77	0.71	0.44	0.94
s77	1.77	1.77	1.69	1.85	s77	1.77	1.77	1.69	1.85	s77	0.16	0.16	0.13	0.19	s97	0.64	0.35	0.00	0.67	s93	0.61	0.71	0.45	0.94
s60	1.84	1.74	0.87	3.09	s60	1.84	1.75	0.87	3.14	s93	0.10	0.15	0.08	0.26	s20	0.63	0.31	0.00	0.88	s135	0.75	0.64	0.34	0.93
s40	1.59	1.69	0.95	2.34	s40	1.59	1.69	0.95	2.34	s60	0.15	0.15	0.09	0.25	s135	0.00	0.31	0.00	1.30	s131	0.68	0.63	0.29	0.85
s113	1.80	1.59	1.03	2.33	s113	1.80	1.59	1.03	2.33	s135	0.16	0.13	0.07	0.24	s131	0.00	0.27	0.00	0.75	s34	0.65	0.62	0.36	0.91
s135	1.89	1.52	0.84	2.96	s135	1.89	1.52	0.84	2.97	s34	0.14	0.13	0.08	0.23	s34	0.00	0.25	0.00	1.11	s83	0.57	0.59	0.40	0.79
s63	1.90	1.51	1.01	2.25	s63	1.90	1.52	1.02	2.26	s20	0.12	0.13	0.10	0.19	s83	0.50	0.22	0.00	0.62	s113	0.68	0.59	0.23	0.85
s34	1.62	1.47	0.84	2.69	s34	1.62	1.47	0.84	2.73	s113	0.12	0.12	0.05	0.21	s113	0.52	0.17	0.00	0.74	s17	0.54	0.56	0.45	0.65
s23	0.94	1.44	0.87	2.13	s23	0.94	1.44	0.86	2.14	s83	0.13	0.12	0.09	0.16	s23	0.00	0.15	0.00	0.60	s63	0.72	0.55	0.30	0.80
s131	1.78	1.42	0.69	2.24	s131	1.78	1.42	0.69	2.24	s131	0.10	0.10	0.06	0.14	s3	0.26	0.14	0.00	0.48	s23	0.27	0.54	0.24	0.79
s43	1.42	1.35	0.87	1.94	s43	1.42	1.35	0.87	1.93	s37	0.10	0.10	0.08	0.12	s29	0.00	0.12	0.00	0.49	s132	0.63	0.53	0.20	0.73
s132	1.72	1.29	0.62	1.85	s132	1.72	1.29	0.63	1.85	s23	0.06	0.10	0.05	0.15	s98	0.00	0.12	0.00	0.46	s20	0.45	0.53	0.36	0.81
s83	1.09	1.24	0.83	1.85	s83	1.09	1.23	0.83	1.84	s63	0.13	0.09	0.05	0.14	s107	0.00	0.12	0.00	0.41	s43	0.56	0.50	0.27	0.74
s129	1.15	1.21	0.93	1.98	s129	1.15	1.21	0.93	1.98	s43	0.11	0.09	0.05	0.13	s43	0.00	0.11	0.00	0.40	s77	0.47	0.48	0.42	0.59
s31	1.13	1.19	0.77	1.67	s31	1.13	1.19	0.78	1.68	s129	0.05	0.09	0.05	0.15	s49	0.00	0.10	0.00	0.44	s98	0.42	0.47	0.22	0.76
s20	0.91	1.18	0.85	2.09	s20	0.91	1.17	0.85	2.09	s3	0.09	0.08	0.06	0.11	s17	0.32	0.08	0.00	0.34	s49	0.24	0.45	0.18	0.74
s98	0.97	1.07	0.67	1.79	s98	0.97	1.07	0.67	1.79	s29	0.06	0.08	0.05	0.11	s132	0.00	0.08	0.00	0.42	s31	0.40	0.42	0.21	0.62
s49	0.74	1.05	0.68	1.75	s49	0.74	1.05	0.68	1.75	s98	0.07	0.07	0.04	0.11	s63	0.00	0.08	0.00	0.31	s29	0.27	0.41	0.20	0.61
s37	1.01	1.01	0.94	1.08	s37	1.01	1.01	0.94	1.08	s49	0.04	0.07	0.03	0.11	s9	0.41	0.06	0.00	0.34	s129	0.36	0.41	0.19	0.78
s29	0.79	0.93	0.75	1.19	s29	0.79	0.94	0.75	1.19	s9	0.09	0.07	0.04	0.09	s109	0.00	0.06	0.00	0.31	s9	0.48	0.41	0.24	0.55
s9	0.94	0.93	0.68	1.16	s9	0.94	0.93	0.68	1.16	s109	0.06	0.07	0.04	0.11	s129	0.00	0.06	0.00	0.53	s3	0.42	0.39	0.25	0.61
s109	0.79	0.86	0.70	1.27	s109	0.79	0.86	0.70	1.27	s132	0.07	0.06	0.02	0.10	s86	0.00	0.04	0.00	0.24	s107	0.42	0.37	0.15	0.66
s107	0.92	0.80	0.55	1.41	s107	0.92	0.80	0.55	1.40	s107	0.06	0.06	0.01	0.09	s54	0.16	0.03	0.00	0.21	s109	0.26	0.34	0.17	0.59
s3	0.68	0.78	0.64	1.15	s3	0.68	0.77	0.64	1.15	s114	0.04	0.05	0.02	0.08	s11	0.00	0.03	0.00	0.21	s37	0.30	0.27	0.22	0.32
s114	0.67	0.74	0.59	0.93	s114	0.67	0.74	0.59	0.93	s54	0.07	0.05	0.02	0.07	s69	0.00	0.03	0.00	0.25	s114	0.17	0.25	0.09	0.43
s74	0.64	0.74	0.58	1.05	s74	0.64	0.74	0.58	1.05	s31	0.05	0.04	0.02	0.07	s114	0.24	0.03	0.00	0.24	s54	0.32	0.25	0.10	0.43
s73	0.72	0.72	0.63	0.95	s73	0.72	0.72	0.63	0.95	s73	0.07	0.04	0.02	0.07	s80	0.00	0.02	0.00	0.19	s86	0.16	0.23	0.08	0.36
s54	0.61	0.67	0.55	0.85	s54	0.61	0.67	0.55	0.85	s80	0.05	0.04	0.02	0.06	s6	0.00	0.02	0.00	0.12	s74	0.11	0.21	0.08	0.45
s11	0.67	0.63	0.51	0.96	s11	0.67	0.63	0.51	0.96	s11	0.04	0.03	0.01	0.06	s31	0.00	0.02	0.00	0.19	s11	0.27	0.20	0.05	0.42
s69	0.56	0.60	0.51	0.85	s69	0.56	0.60	0.51	0.86	s69	0.03	0.03	0.01	0.06	s73	0.00	0.01	0.00	0.22	s73	0.33	0.19	0.09	0.36
s80	0.58	0.59	0.49	0.78	s80	0.58	0.59	0.49	0.77	s86	0.02	0.03	0.01	0.05	s74	0.00	0.01	0.00	0.18	s69	0.15	0.17	0.06	0.38
s86	0.56	0.58	0.40	0.68	s86	0.56	0.58	0.40	0.68	s74	0.02	0.03	0.01	0.06	s77	0.00	0.01	0.00	0.25	s80	0.24	0.17	0.07	0.31
s6	0.33	0.43	0.30	0.61	s6	0.33	0.43	0.30	0.61	s6	0.01	0.02	0.01	0.03	s59	0.00	0.01	0.00	0.10	s6	0.05	0.12	0.04	0.26
s59	0.39	0.43	0.37	0.50	s59	0.39	0.43	0.37	0.50	s59	0.01	0.01	0.00	0.03	s37	0.00	0.00	0.00	0.00	s59	0.06	0.07	0.03	0.18

Sy – symptom (node) identifier, Sa – score of the sample, Bo – mean of the bootstrap, q2.5 – 2.5th percentile, q97.5 – 97.5th percentile
Note: raw scores are used instead of z-scores to avoid inflation of dissimilarities between centrality indices.

Supplementary Table ST5. Stability of TMFG somatoform symptoms networks. Stability of edges and five centrality indices estimated in sample and eight subsamples by case-dropping bootstrap

Estimators: / Subgroups:	Edges	Strength	Expected influence	Eigenvector centrality	Bridge strength	Hybrid centrality
All patients	0.657	0.867	0.872	0.880	0.657	0.829
Women	0.572	0.851	0.845	0.836	0.147	0.757
Men	0.314	0.745	0.732	0.825	0.459	0.757
Older	0.486	0.688	0.684	0.714	0.055	0.700
Younger	0.414	0.802	0.780	0.847	0.532	0.743
1980-2000	0.514	0.800	0.800	0.786	0.074	0.686
2000-2015	0.443	0.771	0.748	0.771	0.147	0.672

CS-coefficients assessed with case-dropping bootstrap analysis (nBoots = 20,000). This procedure evaluates if centrality of indices remains the same after re-estimating the network with fewer cases. The network is repeatedly re-estimated with a larger and larger percentage of cases dropped. This procedure results in CS-coefficients representing the maximum proportion of cases that can be dropped for the correlation between the centrality indices of the full sample and the centrality indices of the subset of cases, to be retained at 0.70 with 95% confidence. Many researchers suggested that CS should be greater than 0.50 (or at least 0.25) for centrality estimates to be interpretable. Our CS-coefficients were frequently much higher.

Comparison 1. Women versus Men Somatoform Symptoms Networks

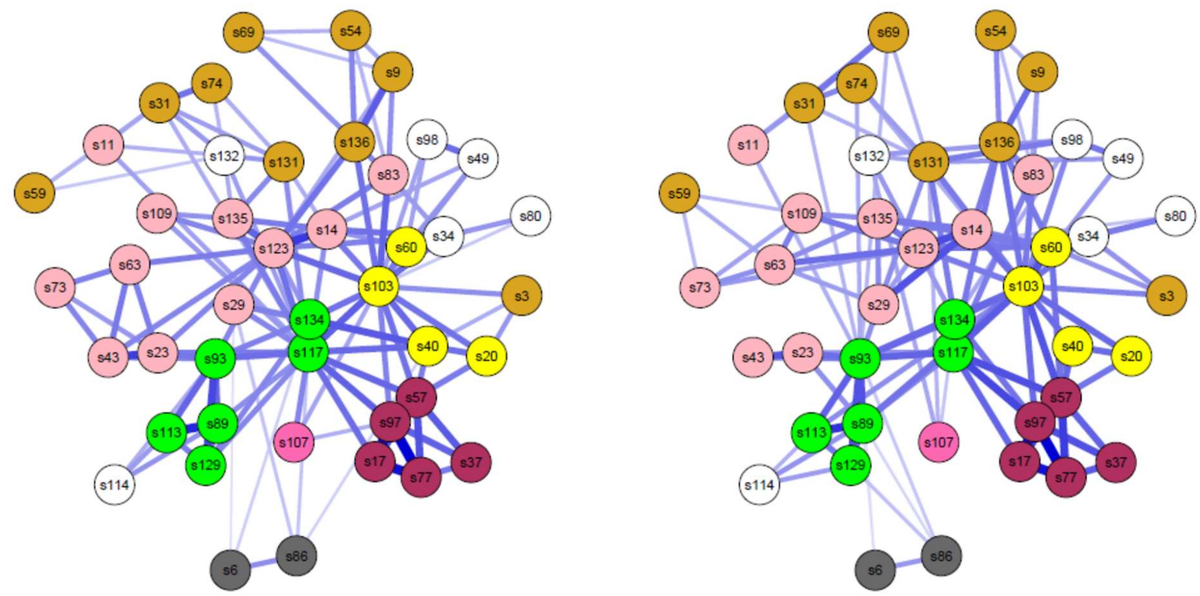

Supplementary Fig. SF5A. Plot with average layout aimed to compare edges; **Women** (left panel) versus **Men** (right panel).

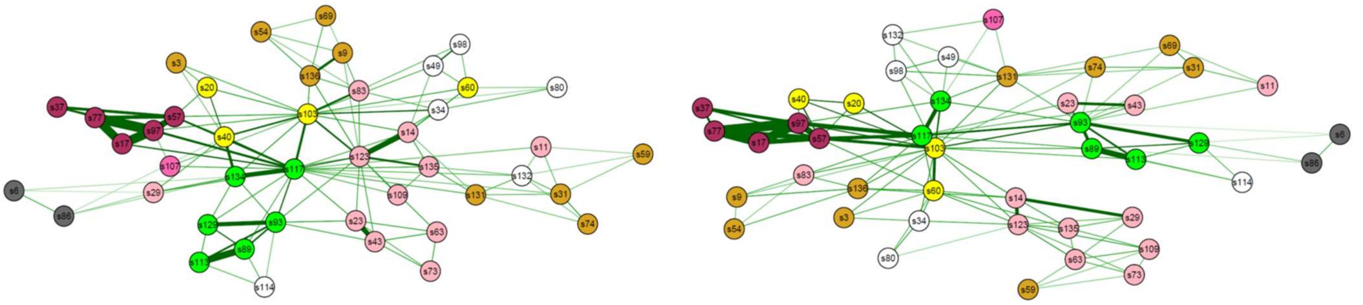

Suplementary Fig. SF5B. Sample network colored plots; **Women** (left panel) versus **Men** (right panel).

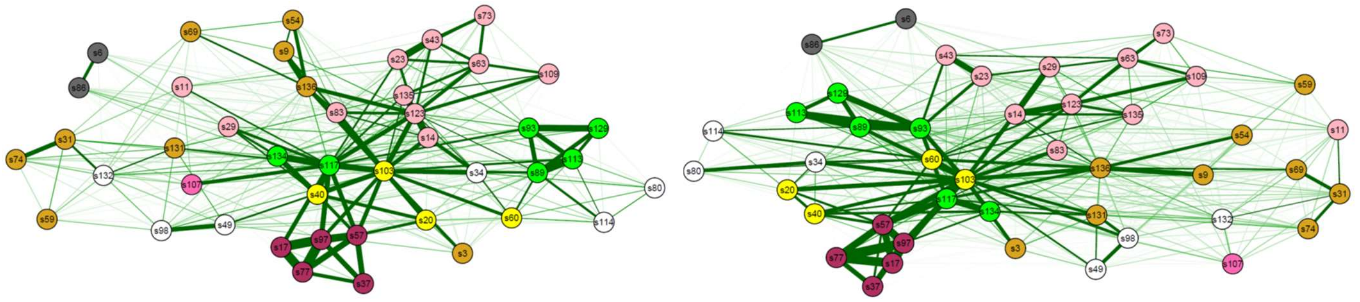

Supplementary Fig. SF5C. Mean bootnet plots (nonparametric bootstrap; nBoots = 20,000); **Women** (left panel) versus **Men** (right panel).

s3 indicates choking/"lump"; s6, fatigue in the morning; s9, vomiting in stress; s11, itching or rashes; s14, dizziness; s17, discovering serious diseases; s20, palpitations; s23, loss of sensitivity in parts of the body; s29, persistent headaches; s31, flatulence or involuntary passing of gas; s34, flushes of blood into the head; s37, ritualistic actions to avoid disease; s40, heart pain; s43, temporary paralyses; s49, dry mouth; s54, loss of appetite; s57, focusing on body functions – e.g., pulse; s59, attacks of hunger – e.g., at night; s60, heat or cold w. reasons; s63, periodic blindness or deafness; s69, diarrhea; s73, transient aphonia; s74, constipation; s77, fears about health and contracting diseases; s80, blushing; s83, faintness; s86, constant fatigue; s89, trembling of legs, hands…; s93, muscle cramps; s97, feelings of having serious diseases; s98, excessive thirst; s103, dyspnea; s107, pains in the sexual organs; s109, hypersensitivity; s113, trembling of the face, eyelids, head…; s114, excessive perspiration; s117, undefined "travelling" pains; s123, disorders of balance; s129, muscle tensions; s131, heartburn; s132, passing urine frequently; s134, muscle pains – e.g., in the back; s135, buzzing in the ears; s136, nausea.

Comparison 1. continued

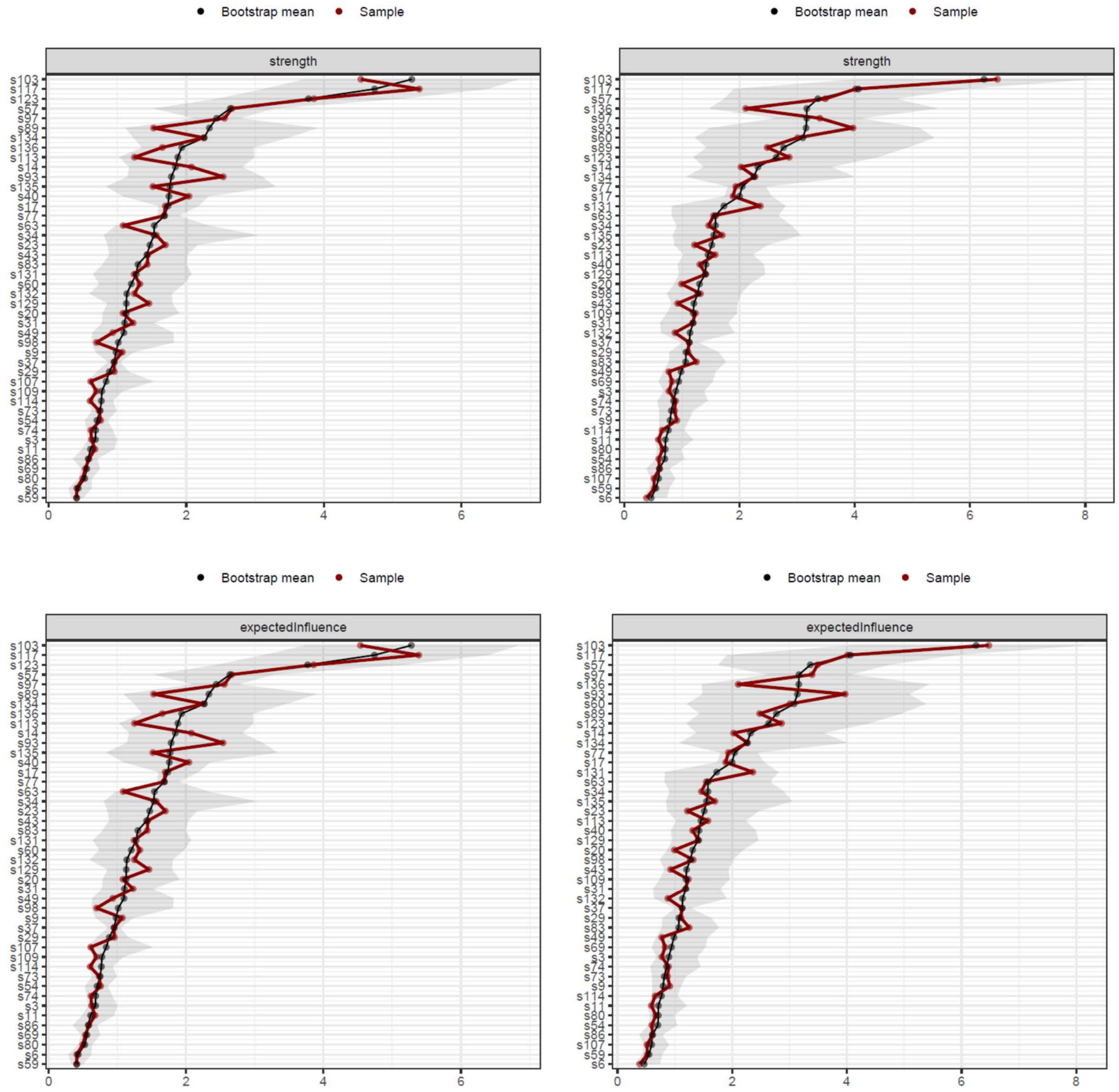

Supplementary Fig. SF6. Bootstrapped (nBoots = 20,000) confidence intervals of estimated node centrality for the network of 44 somatoform symptoms in Women vs. Men.

UPPER LEFT PANEL: strength in **Women**.

LOWER LEFT PANEL: expected influence in **Women**.

UPPER RIGHT PANEL: strength in **Men**.

LOWER RIGHT PANEL: expected influence in **Men**.

The *red line* indicates the sample values and the *gray area* the bootstrapped CIs. Each *horizontal line* represents one node of the network. The order is set from the strongest to the weakest node (according to bootstrap means). The mean of the bootstrap samples (*black line*) was used in ordering the edges. The *y*-axis labels are raw scores.

Comparison 1. continued

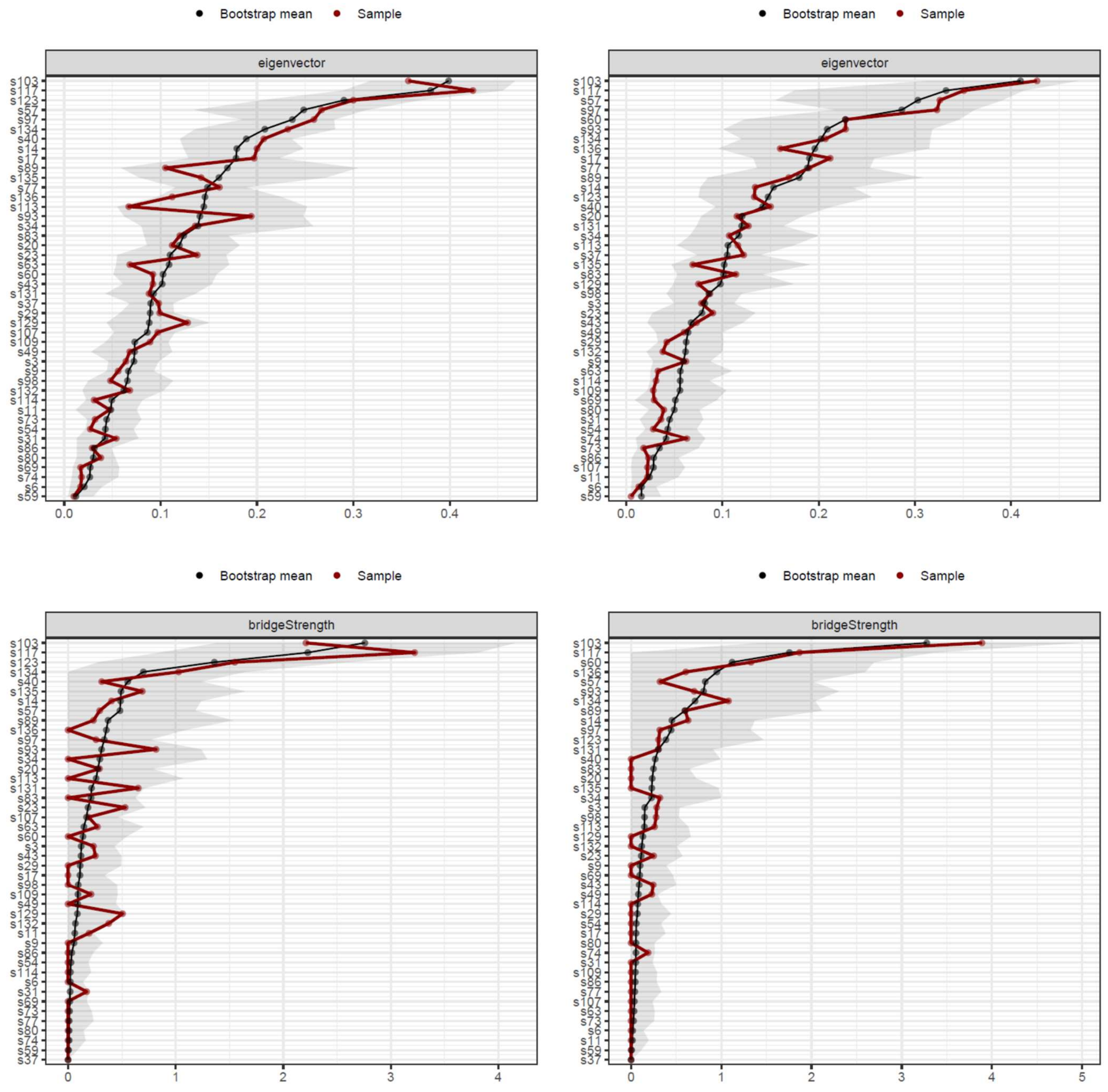

Supplementary Fig. SF7. Bootstrapped (nBoots = 20,000) confidence intervals of estimated node centrality for the network of 44 somatoform symptoms in Women vs. Men.

UPPER LEFT PANEL: eigenvector in **Women**.
UPPER RIGHT PANEL: eigenvector in **Men**.
LOWER LEFT PANEL: bridge strength in **Women**.
LOWER RIGHT PANEL: bridge strength in **Men**.

The *red line* indicates the sample values and the *gray area* the bootstrapped CIs. Each *horizontal line* represents one node of the network. The order is set from the strongest to the weakest node (according to bootstrap means). The mean of the bootstrap samples (*black line*) was used in ordering the edges. The *y*-axis labels are raw scores.

Comparison 1. continued

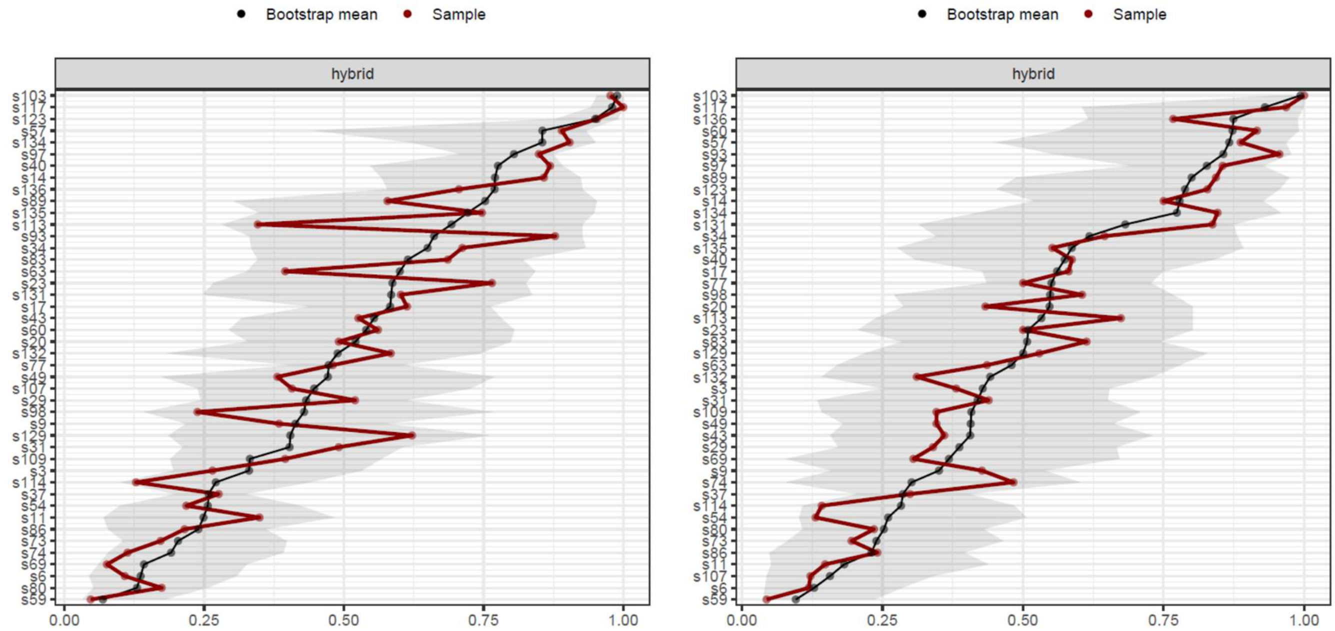

Supplementary Fig. SF8. Bootstrapped (nBoots = 20,000) confidence intervals of estimated node **hybrid** centrality for the network of 44 somatoform symptoms in Women vs. Men.
LEFT PANEL: in **Women**. **RIGHT PANEL:** in **Men**.

The *red line* indicates the sample values and the *gray area* the bootstrapped CIs. Each *horizontal line* represents one node of the network. The order is set from the strongest to the weakest node (according to bootstrap means). The mean of the bootstrap samples (*black line*) was used in ordering the edges. The *y*-axis labels are raw scores.

Supplementary Table ST6. Comparison 1. Twenty edges in the network with the highest mean weights (raw scores): sample and bootstrap estimates

Comparison 1 and edges									
Women					Men				
id	Sa	Bo	q2.5	q97.5	id	Sa	Bo	q2.5	q97.5
**s77--s97**	0.53	0.53	0.50	0.56	**s77--s97**	0.58	0.58	0.54	0.62
**s17--s97**	0.52	0.52	0.49	0.55	**s17--s97**	0.56	0.56	0.51	0.60
**s17--s77**	0.50	0.50	0.46	0.54	**s17--s77**	0.54	0.54	0.49	0.59
**s89--s113**	0.45	0.45	0.42	0.49	**s57--s97**	0.48	0.48	0.43	0.53
**s57--s97**	0.43	0.42	0.39	0.46	**s89--s113**	0.47	0.47	0.42	0.51
**s14--s123**	0.41	0.41	0.38	0.45	**s57--s77**	0.44	0.44	0.39	0.49
**s23--s43**	0.40	0.40	0.36	0.44	**s17--s57**	0.44	0.43	0.38	0.48
**s117--s134**	0.39	0.39	0.35	0.43	**s97--s117**	0.42	0.42	0.37	0.47
**s93--s129**	0.38	0.38	0.35	0.42	**s117--s134**	0.42	0.42	0.37	0.47
**s17--s57**	0.37	0.37	0.34	0.41	**s14--s123**	0.41	0.41	0.36	0.45
**s57--s77**	0.37	0.37	0.33	0.40	**s93--s129**	0.40	0.39	0.35	0.44
**s37--s57**	0.34	0.34	0.30	0.38	**s37--s97**	0.40	0.39	0.33	0.45
**s40--s134**	0.35	0.34	0.28	0.38	**s14--s29**	0.39	0.39	0.33	0.44
**s9--s136**	0.34	0.34	0.30	0.37	**s89--s93**	0.39	0.39	0.33	0.44
**s103--s117**	0.33	0.33	0.30	0.37	**s60--s103**	0.39	0.39	0.33	0.44
**s123--s135**	0.32	0.32	0.29	0.36	**s23--s43**	0.38	0.38	0.32	0.45
**s97--s117**	0.33	0.32	0.00	0.37	**s57--s117**	0.40	0.38	0.00	0.45
**s37--s97**	0.33	0.32	0.00	0.36	**s57--s103**	0.39	0.37	0.00	0.44
**s83--s103**	0.32	0.32	0.28	0.36	**s103--s117**	0.37	0.36	0.00	0.42
**s103--s123**	0.31	0.31	0.28	0.35	**s93--s113**	0.36	0.35	0.00	0.41

id – edge identifier (with codes of the two nodes), Sa – score of the sample, Bo – mean of the bootstrap, q2.5 – 2.5th percentile, q97.5 – 97.5th percentile

Supplementary Table ST7. Comparison 1: sample raw scores and bootstrap estimated means of five centralities

Strength centrality

Women					Men				
Sy	Sa	Bo	q2.5	q97.5	Sy	Sa	Bo	q2.5	q97.5
**s103**	4.54	5.28	3.73	6.83	**s103**	6.48	6.24	4.03	8.12
**s117**	5.39	4.73	3.12	6.40	**s117**	4.01	4.06	1.90	5.91
**s123**	3.86	3.77	2.40	5.13	**s57**	3.49	3.36	1.73	4.71
**s57**	2.66	2.64	1.54	3.28	**s136**	2.11	3.17	1.47	5.44
**s97**	2.56	2.44	2.11	2.75	**s97**	3.39	3.17	2.63	4.09
**s89**	1.53	2.33	1.09	3.89	**s93**	3.97	3.15	1.49	5.13
**s134**	2.25	2.26	1.24	3.49	**s60**	3.01	3.10	1.22	5.38
**s136**	1.65	1.93	1.36	2.98	**s89**	2.48	2.76	1.43	4.92
**s113**	1.25	1.87	1.02	2.96	**s123**	2.86	2.64	1.20	4.39
**s14**	2.08	1.84	1.17	2.81	**s14**	2.02	2.32	1.38	3.59
**s93**	2.54	1.79	1.14	3.12	**s134**	2.27	2.25	1.08	4.00
**s135**	1.51	1.76	0.84	3.31	**s77**	1.93	2.05	1.80	2.45
**s40**	2.04	1.75	1.09	2.34	**s17**	1.89	2.00	1.74	2.56
**s17**	1.70	1.73	1.60	2.17	**s131**	2.36	1.73	0.82	2.80
**s77**	1.68	1.68	1.58	1.89	**s63**	1.55	1.58	0.82	2.67
**s34**	1.56	1.54	0.80	3.05	**s34**	1.46	1.58	0.91	2.90
**s63**	1.08	1.53	0.98	2.32	**s135**	1.70	1.55	0.85	3.06
**s23**	1.70	1.47	0.86	2.16	**s23**	1.22	1.52	0.87	2.48
**s43**	1.44	1.43	0.88	2.01	**s113**	1.58	1.45	1.03	2.23
**s83**	1.43	1.30	0.78	2.00	**s40**	1.31	1.42	0.93	2.43
**s131**	1.24	1.28	0.64	2.07	**s129**	1.42	1.41	0.95	2.44
**s60**	1.33	1.21	0.74	1.98	**s20**	0.99	1.30	0.87	2.18
**s132**	1.25	1.14	0.59	1.79	**s98**	1.32	1.27	0.74	2.02
**s129**	1.46	1.13	0.85	1.81	**s43**	0.93	1.21	0.82	1.96
**s20**	1.08	1.13	0.79	1.91	**s109**	1.24	1.20	0.74	1.95
**s31**	1.23	1.10	0.77	1.52	**s31**	1.19	1.20	0.62	1.80
**s49**	0.93	1.10	0.63	1.84	**s132**	0.88	1.14	0.64	1.91
**s98**	0.70	1.01	0.63	1.81	**s37**	1.13	1.13	1.00	1.27
**s9**	1.07	0.98	0.68	1.25	**s29**	1.11	1.07	0.78	1.64
**s37**	0.95	0.95	0.86	1.04	**s83**	1.25	1.06	0.78	1.76
**s29**	0.96	0.88	0.70	1.16	**s49**	0.77	0.98	0.68	1.58
**s107**	0.61	0.84	0.57	1.52	**s69**	0.83	0.94	0.59	1.53
**s109**	0.70	0.77	0.65	1.07	**s3**	0.77	0.89	0.69	1.38
**s114**	0.60	0.77	0.57	0.93	**s74**	0.89	0.86	0.57	1.45
**s73**	0.73	0.75	0.63	0.99	**s73**	0.87	0.82	0.61	1.19
**s54**	0.76	0.70	0.53	0.86	**s9**	0.91	0.79	0.60	1.12
**s74**	0.62	0.68	0.55	0.93	**s114**	0.66	0.77	0.61	1.04
**s3**	0.62	0.68	0.57	0.99	**s11**	0.59	0.71	0.52	1.19
**s11**	0.67	0.61	0.49	0.96	**s80**	0.67	0.71	0.57	0.99
**s86**	0.58	0.58	0.35	0.70	**s54**	0.60	0.70	0.54	1.04
**s69**	0.53	0.56	0.47	0.75	**s86**	0.61	0.61	0.38	0.78
**s80**	0.50	0.53	0.43	0.63	**s107**	0.51	0.60	0.47	0.88
**s6**	0.41	0.43	0.29	0.62	**s59**	0.52	0.55	0.43	0.78
**s59**	0.41	0.41	0.33	0.50	**s6**	0.39	0.47	0.31	0.74

Expected influence centrality

Women					Men				
Sy	Sa	Bo	q2.5	q97.5	Sy	Sa	Bo	q2.5	q97.5
**s103**	4.54	5.28	3.70	6.84	**s103**	6.48	6.25	4.06	8.11
**s117**	5.39	4.74	3.14	6.41	**s117**	4.01	4.06	1.92	5.90
**s123**	3.86	3.76	2.38	5.10	**s57**	3.49	3.37	1.75	4.75
**s57**	2.66	2.64	1.53	3.27	**s136**	2.11	3.17	1.48	5.49
**s97**	2.56	2.44	2.13	2.76	**s97**	3.39	3.17	2.64	4.08
**s89**	1.53	2.34	1.09	3.90	**s93**	3.97	3.16	1.50	5.11
**s134**	2.25	2.26	1.23	3.50	**s60**	3.01	3.08	1.21	5.39
**s136**	1.65	1.94	1.36	2.95	**s89**	2.48	2.76	1.44	4.96
**s113**	1.25	1.88	1.02	3.00	**s123**	2.86	2.64	1.21	4.38
**s14**	2.08	1.84	1.17	2.82	**s14**	2.02	2.32	1.38	3.59
**s93**	2.54	1.78	1.14	3.09	**s134**	2.27	2.25	1.09	4.01
**s135**	1.51	1.76	0.84	3.30	**s77**	1.93	2.04	1.80	2.45
**s40**	2.04	1.75	1.09	2.34	**s17**	1.89	2.00	1.74	2.57
**s17**	1.70	1.73	1.60	2.19	**s131**	2.36	1.73	0.82	2.82
**s77**	1.68	1.68	1.58	1.89	**s34**	1.46	1.58	0.91	2.89
**s63**	1.08	1.53	0.98	2.32	**s63**	1.55	1.58	0.82	2.68
**s34**	1.56	1.53	0.80	3.05	**s135**	1.70	1.55	0.84	3.06
**s23**	1.70	1.47	0.86	2.15	**s23**	1.22	1.52	0.87	2.48
**s43**	1.44	1.43	0.88	2.03	**s113**	1.58	1.45	1.03	2.24
**s83**	1.43	1.30	0.78	2.00	**s40**	1.31	1.42	0.93	2.44
**s131**	1.24	1.28	0.64	2.09	**s129**	1.42	1.41	0.95	2.45
**s60**	1.33	1.20	0.74	1.98	**s20**	0.99	1.30	0.87	2.18
**s132**	1.25	1.14	0.60	1.80	**s98**	1.32	1.28	0.74	2.04
**s129**	1.46	1.13	0.86	1.78	**s43**	0.93	1.20	0.82	1.95
**s20**	1.08	1.12	0.79	1.91	**s31**	1.19	1.20	0.63	1.80
**s31**	1.23	1.10	0.77	1.51	**s109**	1.24	1.19	0.74	1.93
**s49**	0.93	1.10	0.63	1.82	**s132**	0.88	1.13	0.64	1.90
**s98**	0.70	1.02	0.62	1.82	**s37**	1.13	1.13	1.00	1.27
**s9**	1.07	0.98	0.68	1.25	**s29**	1.11	1.07	0.78	1.63
**s37**	0.95	0.95	0.87	1.04	**s83**	1.25	1.07	0.78	1.79
**s29**	0.96	0.88	0.70	1.16	**s49**	0.77	0.98	0.68	1.56
**s107**	0.61	0.83	0.57	1.51	**s69**	0.83	0.94	0.60	1.53
**s109**	0.70	0.77	0.65	1.08	**s3**	0.77	0.89	0.69	1.38
**s114**	0.60	0.77	0.57	0.93	**s74**	0.89	0.86	0.57	1.47
**s73**	0.73	0.75	0.63	0.99	**s73**	0.87	0.82	0.61	1.20
**s54**	0.76	0.70	0.53	0.86	**s9**	0.91	0.80	0.61	1.11
**s3**	0.62	0.68	0.57	1.01	**s114**	0.66	0.76	0.61	1.05
**s74**	0.62	0.68	0.55	0.93	**s11**	0.59	0.71	0.52	1.20
**s11**	0.67	0.61	0.49	0.94	**s80**	0.67	0.71	0.57	0.99
**s86**	0.58	0.57	0.35	0.70	**s54**	0.60	0.70	0.54	1.04
**s69**	0.53	0.56	0.47	0.75	**s86**	0.61	0.61	0.38	0.78
**s80**	0.50	0.53	0.43	0.63	**s107**	0.51	0.60	0.47	0.88
**s6**	0.41	0.43	0.28	0.62	**s59**	0.52	0.55	0.43	0.78
**s59**	0.41	0.41	0.33	0.50	**s6**	0.39	0.46	0.30	0.74

Eigenvector centrality

Women					Men				
Sy	Sa	Bo	q2.5	q97.5	Sy	Sa	Bo	q2.5	q97.5
**s103**	0.36	0.40	0.32	0.47	**s103**	0.43	0.41	0.31	0.47
**s117**	0.42	0.38	0.28	0.46	**s117**	0.35	0.33	0.17	0.41
**s123**	0.30	0.29	0.20	0.37	**s57**	0.33	0.30	0.16	0.39
**s57**	0.27	0.25	0.13	0.32	**s97**	0.32	0.29	0.22	0.36
**s97**	0.26	0.24	0.18	0.28	**s60**	0.23	0.23	0.12	0.35
**s134**	0.23	0.21	0.13	0.28	**s93**	0.23	0.21	0.10	0.32
**s40**	0.21	0.19	0.12	0.24	**s134**	0.21	0.20	0.12	0.32
**s14**	0.20	0.18	0.12	0.25	**s136**	0.16	0.20	0.10	0.32
**s17**	0.20	0.18	0.13	0.22	**s17**	0.21	0.19	0.13	0.26
**s89**	0.11	0.17	0.06	0.30	**s77**	0.19	0.19	0.14	0.26
**s135**	0.14	0.16	0.08	0.27	**s89**	0.17	0.18	0.09	0.30
**s77**	0.16	0.15	0.12	0.19	**s14**	0.13	0.15	0.08	0.24
**s136**	0.11	0.15	0.10	0.23	**s123**	0.13	0.15	0.08	0.25
**s113**	0.07	0.15	0.06	0.25	**s40**	0.15	0.14	0.09	0.21
**s93**	0.19	0.14	0.06	0.25	**s20**	0.12	0.12	0.08	0.19
**s34**	0.14	0.14	0.08	0.26	**s131**	0.13	0.12	0.07	0.16
**s83**	0.12	0.12	0.08	0.17	**s34**	0.11	0.12	0.07	0.20
**s20**	0.11	0.12	0.09	0.18	**s113**	0.12	0.11	0.05	0.17
**s23**	0.14	0.11	0.06	0.17	**s37**	0.12	0.11	0.08	0.14
**s63**	0.07	0.11	0.05	0.17	**s135**	0.07	0.10	0.05	0.19
**s60**	0.09	0.10	0.05	0.16	**s83**	0.11	0.10	0.05	0.14
**s43**	0.09	0.10	0.06	0.15	**s129**	0.08	0.10	0.05	0.18
**s131**	0.09	0.09	0.04	0.14	**s98**	0.09	0.09	0.04	0.12
**s37**	0.10	0.09	0.07	0.11	**s3**	0.08	0.08	0.05	0.11
**s29**	0.10	0.09	0.06	0.11	**s23**	0.09	0.08	0.03	0.13
**s129**	0.13	0.09	0.05	0.15	**s43**	0.07	0.07	0.02	0.12
**s107**	0.10	0.09	0.05	0.11	**s49**	0.06	0.06	0.03	0.10
**s109**	0.09	0.07	0.04	0.11	**s29**	0.04	0.06	0.03	0.10
**s49**	0.07	0.07	0.03	0.11	**s132**	0.04	0.06	0.02	0.10
**s3**	0.06	0.07	0.05	0.10	**s9**	0.06	0.06	0.03	0.09
**s9**	0.06	0.07	0.04	0.09	**s63**	0.03	0.06	0.03	0.11
**s98**	0.05	0.07	0.02	0.11	**s114**	0.03	0.06	0.03	0.09
**s132**	0.07	0.06	0.02	0.10	**s109**	0.03	0.06	0.02	0.10
**s114**	0.03	0.05	0.02	0.08	**s69**	0.03	0.05	0.01	0.09
**s11**	0.05	0.05	0.01	0.08	**s80**	0.04	0.05	0.02	0.08
**s73**	0.03	0.04	0.02	0.08	**s31**	0.04	0.05	0.01	0.08
**s54**	0.03	0.04	0.02	0.07	**s54**	0.03	0.04	0.02	0.07
**s31**	0.05	0.04	0.01	0.08	**s74**	0.06	0.04	0.01	0.08
**s86**	0.03	0.03	0.01	0.05	**s73**	0.02	0.03	0.01	0.07
**s80**	0.04	0.03	0.01	0.05	**s107**	0.02	0.03	0.01	0.06
**s69**	0.02	0.03	0.01	0.06	**s86**	0.02	0.03	0.01	0.05
**s74**	0.02	0.03	0.01	0.06	**s11**	0.02	0.02	0.01	0.05
**s6**	0.02	0.02	0.01	0.04	**s6**	0.01	0.02	0.01	0.03
**s59**	0.01	0.01	0.00	0.03	**s59**	0.01	0.02	0.01	0.04

Bridge strength centrality

Women					Men				
Sy	Sa	Bo	q2.5	q97.5	Sy	Sa	Bo	q2.5	q97.5
**s103**	2.21	2.74	1.35	4.16	**s103**	3.89	3.27	1.43	4.95
**s117**	2.43	2.24	0.85	3.82	**s117**	1.87	1.76	0.00	3.35
**s123**	1.55	1.36	0.28	2.59	**s60**	1.33	1.12	0.00	2.68
**s134**	0.51	0.70	0.00	1.65	**s136**	0.61	0.94	0.00	2.56
**s40**	0.31	0.55	0.00	1.24	**s57**	0.32	0.83	0.00	1.99
**s135**	0.69	0.49	0.00	1.66	**s93**	0.70	0.79	0.00	2.34
**s14**	0.40	0.48	0.00	1.25	**s134**	1.08	0.70	0.00	2.03
**s57**	0.29	0.48	0.00	1.18	**s89**	0.60	0.61	0.00	2.09
**s89**	0.00	0.38	0.00	1.59	**s14**	0.63	0.45	0.00	1.38
**s136**	0.00	0.35	0.00	1.08	**s97**	0.32	0.45	0.00	1.33
**s97**	0.26	0.34	0.00	0.77	**s123**	0.30	0.38	0.00	1.50
**s93**	0.79	0.32	0.00	1.24	**s131**	0.30	0.30	0.00	0.90
**s34**	0.00	0.29	0.00	1.27	**s40**	0.00	0.27	0.00	0.99
**s20**	0.29	0.28	0.00	0.79	**s83**	0.00	0.25	0.00	0.64
**s113**	0.00	0.26	0.00	1.07	**s20**	0.00	0.24	0.00	0.73
**s131**	0.65	0.22	0.00	0.71	**s34**	0.32	0.23	0.00	0.99
**s83**	0.00	0.21	0.00	0.62	**s135**	0.00	0.23	0.00	0.96
**s23**	0.82	0.19	0.00	0.70	**s3**	0.28	0.15	0.00	0.54
**s107**	0.19	0.17	0.00	0.47	**s113**	0.26	0.15	0.00	0.67
**s63**	0.27	0.15	0.00	0.69	**s98**	0.28	0.15	0.00	0.52
**s60**	0.00	0.14	0.00	0.55	**s129**	0.00	0.13	0.00	0.67
**s3**	0.24	0.12	0.00	0.43	**s132**	0.00	0.12	0.00	0.50
**s43**	0.51	0.12	0.00	0.50	**s23**	0.25	0.11	0.00	0.57
**s29**	0.00	0.11	0.00	0.49	**s9**	0.00	0.10	0.00	0.39
**s17**	0.00	0.11	0.00	0.34	**s69**	0.00	0.10	0.00	0.47
**s98**	0.00	0.09	0.00	0.45	**s43**	0.25	0.08	0.00	0.41
**s109**	0.21	0.09	0.00	0.46	**s49**	0.23	0.08	0.00	0.29
**s49**	0.00	0.09	0.00	0.44	**s114**	0.00	0.08	0.00	0.38
**s129**	0.00	0.09	0.00	0.53	**s29**	0.00	0.07	0.00	0.42
**s132**	0.38	0.07	0.00	0.40	**s54**	0.00	0.06	0.00	0.31
**s11**	0.20	0.06	0.00	0.24	**s80**	0.00	0.06	0.00	0.27
**s9**	0.00	0.05	0.00	0.32	**s17**	0.00	0.05	0.00	0.38
**s86**	0.00	0.04	0.00	0.23	**s31**	0.00	0.05	0.00	0.24
**s54**	0.00	0.03	0.00	0.19	**s74**	0.19	0.05	0.00	0.26
**s114**	0.00	0.02	0.00	0.22	**s109**	0.00	0.05	0.00	0.29
**s6**	0.00	0.02	0.00	0.14	**s86**	0.00	0.04	0.00	0.28
**s31**	0.17	0.02	0.00	0.19	**s77**	0.00	0.04	0.00	0.33
**s69**	0.00	0.02	0.00	0.17	**s107**	0.00	0.04	0.00	0.24
**s73**	0.00	0.01	0.00	0.22	**s63**	0.00	0.03	0.00	0.26
**s77**	0.00	0.01	0.00	0.23	**s73**	0.00	0.03	0.00	0.25
**s74**	0.00	0.01	0.00	0.16	**s6**	0.00	0.02	0.00	0.13
**s80**	0.00	0.01	0.00	0.14	**s11**	0.00	0.01	0.00	0.20
**s59**	0.00	0.00	0.00	0.09	**s59**	0.00	0.01	0.00	0.12
**s37**	0.00	0.00	0.00	0.00	**s37**	0.00	0.00	0.00	0.00

Hybrid centrality

Women					Men				
Sy	Sa	Bo	q2.5	q97.5	Sy	Sa	Bo	q2.5	q97.5
**s103**	0.98	0.99	0.95	1.00	**s103**	1.00	0.99	0.95	1.00
**s117**	1.00	0.98	0.94	1.00	**s117**	0.97	0.93	0.61	1.00
**s123**	0.95	0.95	0.88	0.99	**s136**	0.77	0.87	0.62	0.99
**s57**	0.89	0.86	0.45	0.93	**s60**	0.92	0.87	0.57	0.99
**s134**	0.90	0.86	0.62	0.95	**s57**	0.89	0.87	0.45	0.97
**s97**	0.85	0.80	0.71	0.88	**s93**	0.96	0.86	0.56	0.98
**s40**	0.87	0.78	0.55	0.89	**s97**	0.86	0.83	0.69	0.93
**s14**	0.86	0.77	0.57	0.92	**s89**	0.84	0.80	0.49	0.97
**s136**	0.71	0.77	0.58	0.93	**s123**	0.83	0.79	0.45	0.96
**s89**	0.58	0.75	0.30	0.95	**s14**	0.75	0.78	0.52	0.93
**s135**	0.75	0.72	0.35	0.95	**s134**	0.85	0.77	0.42	0.96
**s113**	0.35	0.69	0.28	0.93	**s131**	0.84	0.68	0.31	0.88
**s93**	0.88	0.66	0.33	0.93	**s34**	0.65	0.62	0.35	0.88
**s34**	0.71	0.65	0.33	0.93	**s135**	0.55	0.59	0.28	0.89
**s83**	0.69	0.61	0.35	0.82	**s40**	0.59	0.57	0.31	0.84
**s63**	0.40	0.60	0.33	0.84	**s17**	0.58	0.56	0.43	0.75
**s23**	0.77	0.59	0.27	0.83	**s77**	0.50	0.55	0.44	0.73
**s131**	0.60	0.58	0.25	0.84	**s98**	0.61	0.55	0.27	0.77
**s17**	0.61	0.58	0.44	0.76	**s20**	0.43	0.55	0.29	0.80
**s43**	0.53	0.55	0.32	0.78	**s113**	0.67	0.53	0.24	0.80
**s60**	0.56	0.54	0.29	0.81	**s23**	0.50	0.51	0.21	0.80
**s20**	0.49	0.52	0.33	0.80	**s83**	0.61	0.51	0.29	0.74
**s132**	0.58	0.49	0.18	0.74	**s129**	0.53	0.50	0.22	0.83
**s77**	0.48	0.47	0.41	0.61	**s63**	0.44	0.48	0.17	0.78
**s49**	0.38	0.47	0.17	0.77	**s132**	0.31	0.44	0.14	0.73
**s107**	0.41	0.45	0.24	0.73	**s3**	0.38	0.43	0.25	0.65
**s29**	0.52	0.43	0.24	0.61	**s31**	0.44	0.42	0.13	0.65
**s98**	0.24	0.43	0.14	0.77	**s109**	0.35	0.41	0.14	0.71
**s9**	0.38	0.41	0.22	0.57	**s49**	0.35	0.41	0.18	0.66
**s129**	0.62	0.40	0.19	0.76	**s43**	0.36	0.41	0.16	0.73
**s31**	0.49	0.40	0.21	0.61	**s29**	0.34	0.39	0.17	0.67
**s109**	0.40	0.33	0.19	0.57	**s69**	0.31	0.37	0.08	0.67
**s3**	0.27	0.33	0.20	0.53	**s9**	0.43	0.35	0.16	0.54
**s114**	0.13	0.27	0.10	0.44	**s74**	0.48	0.30	0.08	0.60
**s37**	0.28	0.26	0.20	0.32	**s37**	0.30	0.29	0.21	0.40
**s54**	0.22	0.26	0.10	0.42	**s114**	0.14	0.28	0.11	0.49
**s11**	0.35	0.25	0.07	0.48	**s54**	0.13	0.26	0.10	0.51
**s86**	0.22	0.24	0.08	0.36	**s80**	0.24	0.25	0.11	0.43
**s73**	0.17	0.20	0.11	0.40	**s73**	0.20	0.24	0.08	0.47
**s74**	0.11	0.19	0.08	0.39	**s86**	0.24	0.23	0.05	0.40
**s69**	0.08	0.14	0.06	0.33	**s11**	0.15	0.18	0.05	0.44
**s6**	0.11	0.14	0.04	0.31	**s107**	0.12	0.16	0.04	0.34
**s80**	0.17	0.13	0.05	0.26	**s6**	0.12	0.13	0.04	0.29
**s59**	0.05	0.07	0.03	0.18	**s59**	0.04	0.10	0.04	0.24

Sy – symptom (node) identifier, Sa – score of the sample, Bo – mean of the bootstrap, q2.5 – 2.5th percentile, q97.5 – 97.5th percentile

Note: raw scores are used instead of z-scores to avoid inflation of dissimilarities between centrality indices.

Comparison 2. Older Patients versus Younger Patients Somatoform Symptoms Networks

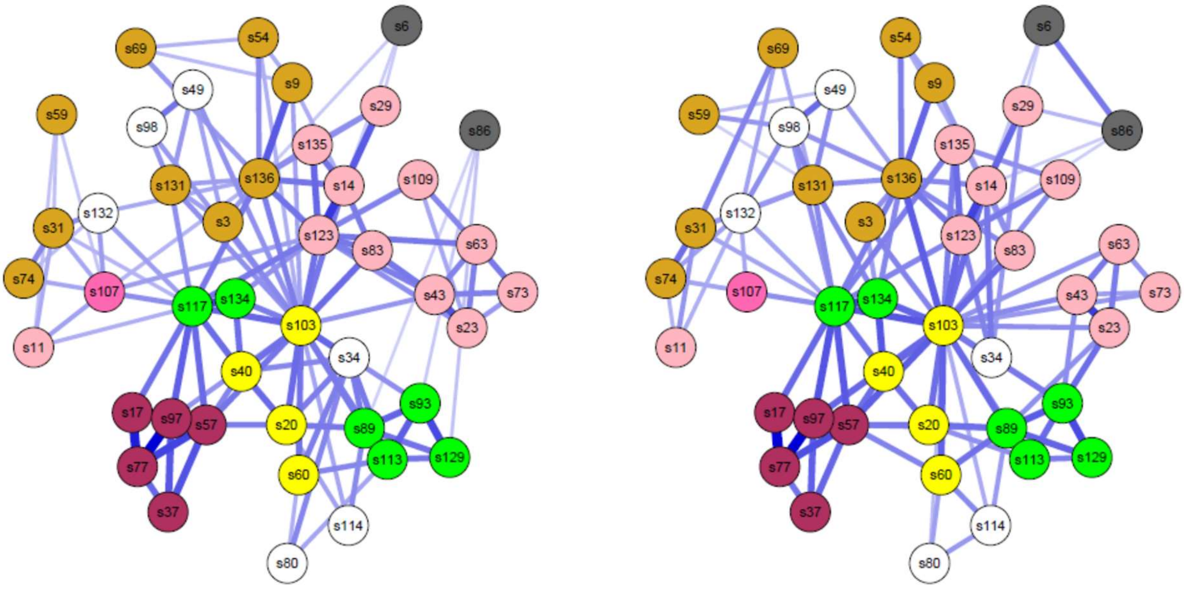

Supplementary Fig. SF9A. Plot with average layout aimed to compare edges; **Older Patients** (left panel) versus **Younger Patients** (right panel).

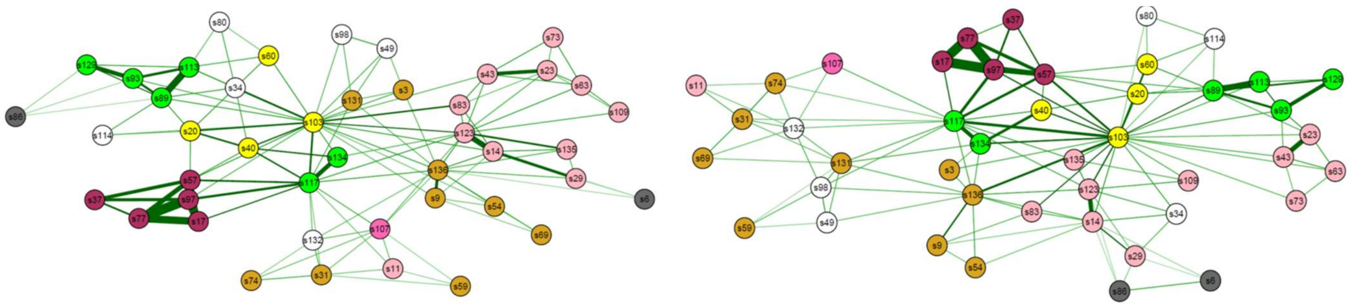

Supplementary Fig. SF9B. Sample network colored plots; **Older Patients** (left panel) versus **Younger Patients** (right panel).

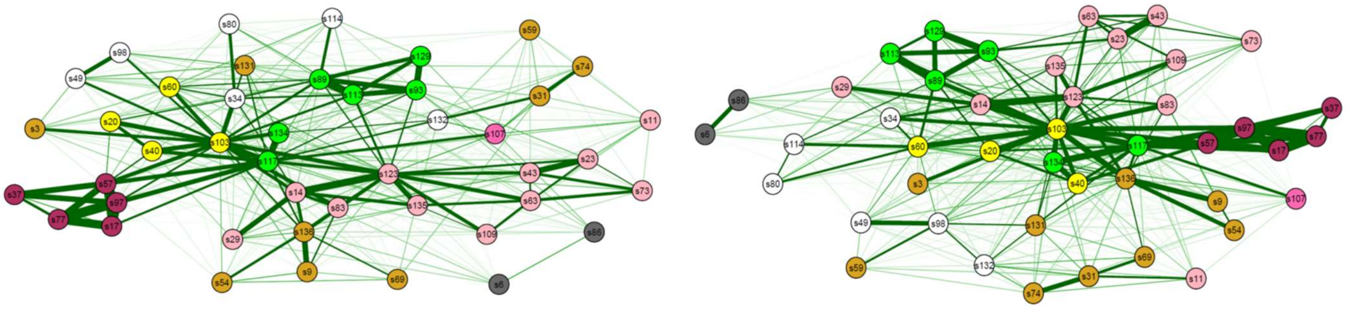

Supplementary Fig. SF9C. Mean bootnet plots (nonparametric bootstrap; nBoots = 20,000); **Older Patients** (left panel) versus **Younger Patients** (right panel).

s3 indicates choking/"lump"; s6, fatigue in the morning; s9, vomiting in stress; s11, itching or rashes; s14, dizziness; s17, discovering serious diseases; s20, palpitations; s23, loss of sensitivity in parts of the body; s29, persistent headaches; s31, flatulence or involuntary passing of gas; s34, flushes of blood into the head; s37, ritualistic actions to avoid disease; s40, heart pain; s43, temporary paralyses; s49, dry mouth; s54, loss of appetite; s57, focusing on body functions – e.g., pulse; s59, attacks of hunger – e.g., at night; s60, heat or cold w. reasons; s63, periodic blindness or deafness; s69, diarrhea; s73, transient aphonia; s74, constipation; s77, fears about health and contracting diseases; s80, blushing; s83, faintness; s86, constant fatigue; s89, trembling of legs, hands…; s93, muscle cramps; s97, feelings of having serious diseases; s98, excessive thirst; s103, dyspnea; s107, pains in the sexual organs; s109, hypersensitivity; s113, trembling of the face, eyelids, head…; s114, excessive perspiration; s117, undefined "travelling" pains; s123, disorders of balance; s129, muscle tensions; s131, heartburn; s132, passing urine frequently; s134, muscle pains – e.g., in the back; s135, buzzing in the ears; s136, nausea.

Comparison 2. continued

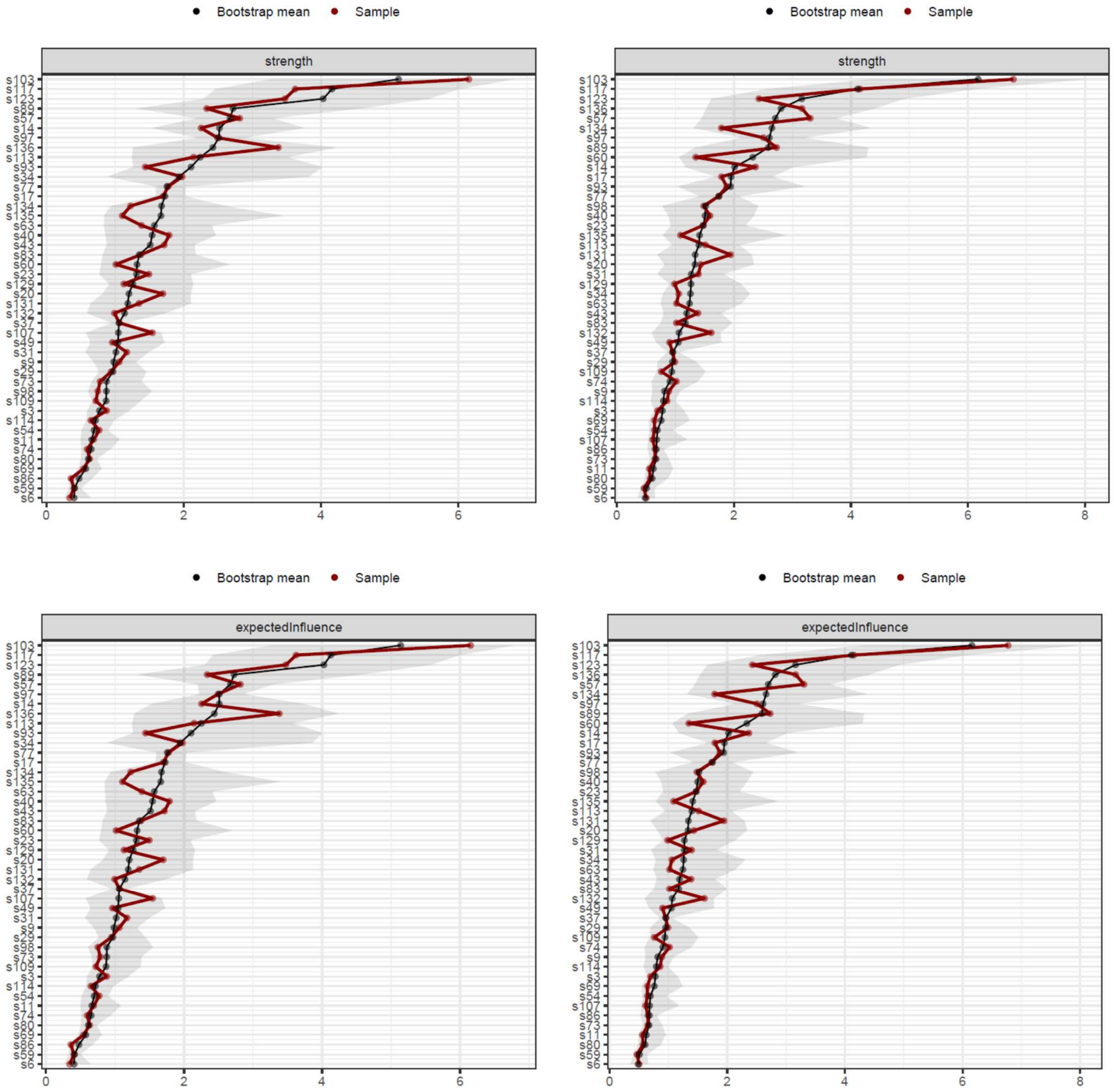

Supplementary Fig. SF10. Bootstrapped (nBoots = 20,000) confidence intervals of estimated node centrality for the network of 44 somatoform symptoms in older vs. younger patients.

UPPER LEFT PANEL: strength; Older Patients.
UPPER RIGHT PANEL: strength; Younger Patients.
LOWER LEFT PANEL: expected influence; Older Patients.
LOWER RIGHT PANEL: expected influence; Younger Patients.

The *red line* indicates the sample values and the *gray area* the bootstrapped CIs. Each *horizontal line* represents one node of the network. The order is set from the strongest to the weakest node (according to bootstrap means). The mean of the bootstrap samples (*black line*) was used in ordering the edges. The *y*-axis labels are raw scores.

Comparison 2. continued

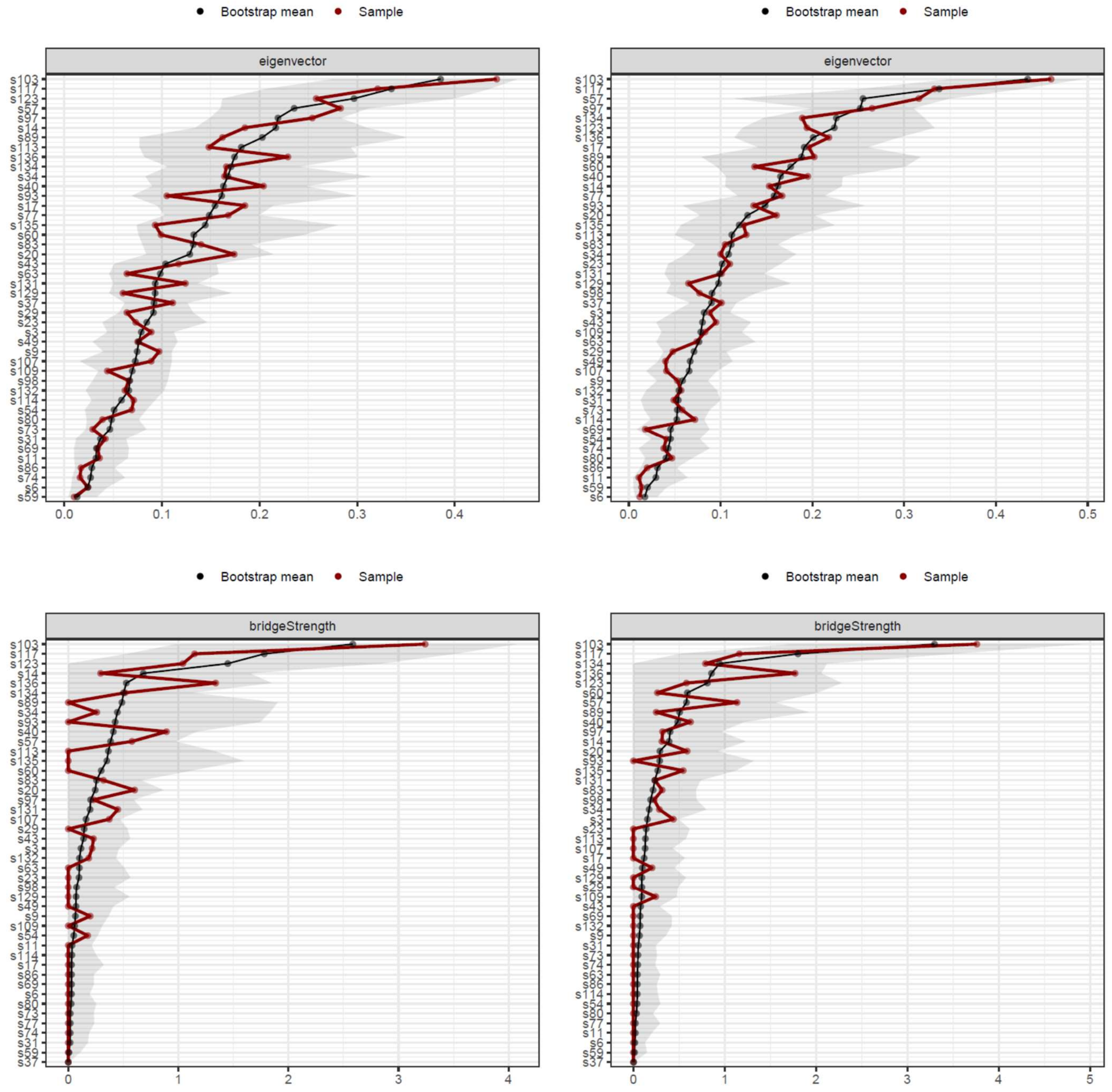

Supplementary Fig. SF11. Bootstrapped (nBoots = 20,000) confidence intervals of estimated node centrality for the network of 44 somatoform symptoms in Older vs. Younger patients.

UPPER LEFT PANEL: eigenvector; **Older Patients**.
LOWER LEFT PANEL: bridge strength; **Older Patients**.

UPPER RIGHT PANEL: eigenvector; **Younger Patients**.
LOWER RIGHT PANEL: bridge strength; **Younger Patients**.

The *red line* indicates the sample values and the *gray area* the bootstrapped CIs. Each *horizontal line* represents one node of the network. The order is set from the strongest to the weakest node (according to bootstrap means). The mean of the bootstrap samples (*black line*) was used in ordering the edges. The *y*-axis labels are raw scores**.**

Comparison 2. continued

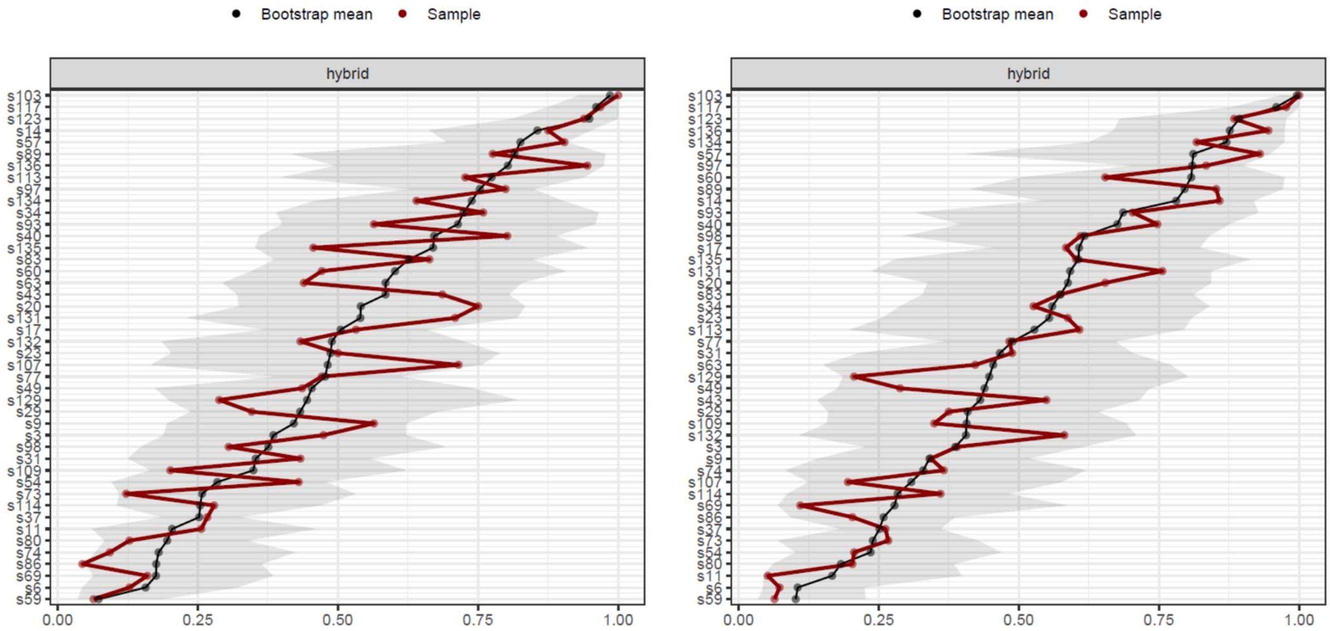

Supplementary Fig. SF12. Bootstrapped (nBoots = 20,000) confidence intervals of estimated node **hybrid** centrality for the network of 44 somatoform symptoms in Older vs. Younger patients.

LEFT PANEL: in **Older Patients**. **RIGHT PANEL:** in **Younger Patients**.

The *red line* indicates the sample values and the *gray area* the bootstrapped CIs. Each *horizontal line* represents one node of the network. The order is set from the strongest to the weakest node (according to bootstrap means). The mean of the bootstrap samples (*black line*) was used in ordering the edges. The *y*-axis labels are raw scores.

Supplementary Table ST8. Comparison 2. Twenty edges in the network with the highest mean weights (raw scores): sample and bootstrap estimates

Comparison 2 and edges									
Older					Younger				
id	Sa	Bo	q2.5	q97.5	id	Sa	Bo	q2.5	q97.5
**s77--s97**	0.55	0.55	0.52	0.59	**s77--s97**	0.54	0.54	0.51	0.58
**s17--s97**	0.52	0.52	0.48	0.56	**s17--s97**	0.54	0.54	0.50	0.54
**s17--s77**	0.49	0.49	0.44	0.53	**s17--s77**	0.53	0.53	0.49	0.53
**s57--s97**	0.48	0.48	0.44	0.52	**s89--s113**	0.44	0.44	0.40	0.50
**s89--s113**	0.47	0.47	0.43	0.51	**s57--s97**	0.41	0.41	0.37	0.49
**s14--s123**	0.43	0.43	0.39	0.47	**s23--s43**	0.41	0.41	0.36	0.46
**s57--s77**	0.41	0.41	0.37	0.45	**s17--s57**	0.40	0.40	0.36	0.46
**s117--s134**	0.40	0.40	0.36	0.44	**s14--s123**	0.40	0.40	0.36	0.45
**s93--s129**	0.40	0.40	0.35	0.44	**s117--s134**	0.39	0.38	0.34	0.45
**s17--s57**	0.38	0.38	0.34	0.43	**s93--s129**	0.38	0.38	0.34	0.43
**s23--s43**	0.38	0.37	0.32	0.42	**s57--s77**	0.37	0.37	0.33	0.41
**s37--s57**	0.37	0.37	0.32	0.42	**s97--s117**	0.36	0.36	0.32	0.39
**s9--s136**	0.36	0.36	0.32	0.40	**s103--s117**	0.36	0.36	0.32	0.39
**s37--s97**	0.36	0.36	0.00	0.41	**s40--s134**	0.36	0.35	0.00	0.39
**s93--s113**	0.35	0.33	0.00	0.39	**s103--s136**	0.34	0.34	0.30	0.38
**s89--s93**	0.34	0.33	0.27	0.38	**s89--s93**	0.34	0.34	0.30	0.38
**s14--s29**	0.36	0.33	0.00	0.40	**s60--s103**	0.33	0.33	0.29	0.39
**s103--s117**	0.33	0.33	0.28	0.37	**s9--s136**	0.33	0.33	0.29	0.39
**s123--s135**	0.33	0.32	0.28	0.37	**s37--s97**	0.34	0.32	0.00	0.37
**s34--s103**	0.33	0.31	0.00	0.37	**s89--s129**	0.33	0.32	0.00	0.38

id – edge identifier (with codes of the two nodes), Sa – score of the sample, Bo – mean of the bootstrap, q2.5 – 2.5th percentile, q97.5 – 97.5th percentile

Supplementary Table ST9. Comparison 2: sample raw scores and bootstrap estimated means of five centralities

Strength centrality

Older					Younger				
Sy	Sa	Bo	q2.5	q97.5	Sy	Sa	Bo	q2.5	q97.5
**s103**	6.15	5.14	3.29	6.80	**s103**	6.78	6.18	4.35	8.06
**s117**	3.62	4.14	2.44	6.03	**s117**	4.15	4.10	2.55	6.10
**s123**	3.48	4.02	2.23	5.58	**s123**	2.43	3.17	1.66	4.94
**s89**	2.34	2.72	1.33	4.57	**s136**	3.16	2.80	1.53	4.42
**s57**	2.82	2.67	2.23	3.30	**s57**	3.30	2.69	1.45	3.74
**s14**	2.25	2.52	1.52	3.76	**s134**	1.79	2.66	1.31	4.36
**s97**	2.49	2.51	2.21	2.88	**s97**	2.51	2.61	2.16	3.20
**s136**	3.38	2.43	1.27	4.20	**s89**	2.73	2.60	1.33	4.31
**s113**	2.14	2.24	1.23	3.52	**s60**	1.35	2.32	1.06	4.22
**s93**	1.44	2.11	1.26	4.00	**s14**	2.37	2.01	1.18	3.01
**s34**	1.98	1.93	0.89	3.81	**s17**	1.79	1.95	1.69	2.67
**s77**	1.78	1.76	1.60	2.04	**s93**	1.88	1.94	1.04	3.22
**s17**	1.70	1.73	1.59	2.02	**s77**	1.74	1.75	1.43	2.09
**s134**	1.23	1.68	0.94	2.61	**s98**	1.49	1.52	0.77	2.44
**s135**	1.11	1.66	0.85	3.41	**s40**	1.59	1.50	0.88	2.33
**s63**	1.39	1.57	0.85	2.40	**s23**	1.47	1.47	0.92	2.19
**s40**	1.79	1.54	0.86	2.46	**s135**	1.09	1.41	0.79	2.84
**s43**	1.72	1.52	0.89	2.17	**s113**	1.51	1.40	0.95	2.08
**s83**	1.38	1.35	0.82	2.18	**s131**	1.95	1.35	0.69	2.29
**s60**	1.01	1.32	0.80	2.66	**s20**	1.43	1.33	0.82	2.33
**s23**	1.50	1.32	0.77	2.11	**s31**	1.39	1.28	0.71	1.81
**s129**	1.13	1.27	0.92	2.17	**s129**	0.99	1.27	0.91	2.13
**s20**	1.70	1.21	0.82	2.09	**s34**	1.06	1.26	0.79	2.28
**s131**	1.35	1.19	0.65	2.13	**s63**	1.02	1.25	0.75	2.15
**s132**	0.99	1.15	0.59	1.73	**s43**	1.39	1.19	0.80	1.79
**s37**	1.06	1.06	0.96	1.16	**s83**	1.02	1.18	0.75	2.02
**s49**	0.96	1.05	0.65	1.73	**s132**	1.61	1.06	0.60	1.77
**s107**	1.54	1.05	0.57	1.67	**s49**	0.90	1.05	0.67	1.79
**s31**	1.17	1.02	0.58	1.48	**s37**	0.95	0.96	0.86	1.06
**s9**	1.06	0.98	0.69	1.31	**s29**	0.99	0.95	0.70	1.34
**s29**	0.95	0.97	0.77	1.45	**s109**	0.76	0.94	0.68	1.53
**s73**	0.78	0.88	0.67	1.40	**s74**	1.02	0.91	0.59	1.39
**s98**	0.75	0.88	0.61	1.54	**s9**	0.89	0.82	0.61	1.09
**s109**	0.72	0.87	0.67	1.37	**s114**	0.86	0.80	0.58	1.00
**s3**	0.88	0.77	0.59	1.20	**s3**	0.70	0.78	0.64	1.15
**s114**	0.65	0.72	0.58	0.97	**s69**	0.65	0.76	0.55	1.25
**s54**	0.77	0.70	0.51	0.90	**s54**	0.64	0.69	0.56	0.95
**s11**	0.69	0.66	0.50	1.06	**s107**	0.61	0.68	0.54	1.18
**s74**	0.60	0.65	0.51	0.93	**s86**	0.65	0.68	0.53	0.80
**s80**	0.63	0.61	0.51	0.74	**s73**	0.65	0.67	0.57	0.89
**s69**	0.54	0.58	0.47	0.81	**s11**	0.56	0.63	0.49	0.95
**s86**	0.36	0.48	0.32	0.67	**s80**	0.57	0.60	0.48	0.87
**s59**	0.40	0.41	0.33	0.50	**s59**	0.46	0.51	0.41	0.65
**s6**	0.34	0.40	0.26	0.65	**s6**	0.50	0.49	0.35	0.66

Expected influence centrality

Older					Younger				
Sy	Sa	Bo	q2.5	q97.5	Sy	Sa	Bo	q2.5	q97.5
**s103**	6.15	5.13	3.27	6.78	**s103**	6.78	6.17	4.33	8.03
**s117**	3.62	4.14	2.44	5.98	**s117**	4.15	4.11	2.56	6.09
**s123**	3.48	4.04	2.28	5.61	**s123**	2.43	3.18	1.67	4.93
**s89**	2.34	2.72	1.33	4.60	**s136**	3.16	2.81	1.56	4.40
**s57**	2.82	2.67	2.21	3.29	**s57**	3.30	2.71	1.45	3.74
**s14**	2.25	2.52	1.54	3.75	**s134**	1.79	2.65	1.31	4.37
**s97**	2.49	2.51	2.23	2.88	**s97**	2.51	2.61	2.15	3.19
**s136**	3.38	2.43	1.27	4.20	**s89**	2.73	2.59	1.34	4.32
**s113**	2.14	2.24	1.24	3.52	**s60**	1.35	2.31	1.04	4.25
**s93**	1.44	2.12	1.27	4.01	**s14**	2.37	2.03	1.18	3.04
**s34**	1.98	1.94	0.89	3.85	**s17**	1.79	1.95	1.69	2.66
**s77**	1.78	1.76	1.60	2.03	**s93**	1.88	1.94	1.04	3.21
**s17**	1.70	1.73	1.59	2.01	**s77**	1.74	1.75	1.42	2.09
**s134**	1.23	1.67	0.95	2.59	**s98**	1.49	1.52	0.77	2.42
**s135**	1.11	1.67	0.85	3.44	**s40**	1.59	1.50	0.88	2.32
**s63**	1.39	1.56	0.84	2.41	**s23**	1.47	1.47	0.91	2.19
**s40**	1.79	1.54	0.86	2.47	**s135**	1.09	1.41	0.79	2.88
**s43**	1.72	1.51	0.88	2.16	**s113**	1.51	1.40	0.95	2.07
**s83**	1.38	1.35	0.82	2.17	**s131**	1.95	1.34	0.69	2.29
**s60**	1.01	1.32	0.80	2.70	**s20**	1.43	1.33	0.82	2.34
**s23**	1.50	1.31	0.77	2.10	**s31**	1.39	1.27	0.70	1.82
**s129**	1.13	1.26	0.92	2.16	**s129**	0.99	1.27	0.91	2.15
**s20**	1.70	1.21	0.82	2.11	**s34**	1.06	1.26	0.79	2.29
**s131**	1.35	1.19	0.65	2.12	**s63**	1.02	1.25	0.75	2.14
**s132**	0.99	1.14	0.59	1.73	**s43**	1.39	1.19	0.80	1.80
**s37**	1.06	1.06	0.96	1.16	**s83**	1.02	1.18	0.75	2.00
**s107**	1.54	1.05	0.58	1.67	**s132**	1.61	1.06	0.60	1.77
**s49**	0.96	1.04	0.64	1.72	**s49**	0.90	1.05	0.67	1.80
**s31**	1.17	1.01	0.57	1.49	**s37**	0.95	0.96	0.86	1.06
**s9**	1.06	0.98	0.69	1.32	**s29**	0.99	0.95	0.70	1.35
**s29**	0.95	0.97	0.77	1.45	**s109**	0.76	0.95	0.68	1.54
**s73**	0.78	0.88	0.67	1.38	**s74**	1.02	0.91	0.59	1.39
**s98**	0.75	0.88	0.61	1.54	**s9**	0.89	0.81	0.61	1.08
**s109**	0.72	0.87	0.67	1.38	**s114**	0.86	0.80	0.58	1.00
**s3**	0.88	0.77	0.60	1.20	**s3**	0.70	0.78	0.64	1.16
**s114**	0.65	0.71	0.58	0.98	**s69**	0.65	0.76	0.55	1.25
**s54**	0.77	0.70	0.51	0.90	**s54**	0.64	0.70	0.56	0.96
**s11**	0.69	0.66	0.50	1.06	**s107**	0.61	0.68	0.54	1.19
**s74**	0.60	0.65	0.51	0.93	**s86**	0.65	0.68	0.53	0.80
**s80**	0.63	0.61	0.51	0.74	**s73**	0.65	0.67	0.57	0.89
**s69**	0.54	0.58	0.47	0.81	**s11**	0.56	0.63	0.49	0.95
**s86**	0.36	0.48	0.32	0.67	**s80**	0.57	0.60	0.48	0.87
**s59**	0.40	0.41	0.33	0.50	**s59**	0.46	0.51	0.41	0.65
**s6**	0.34	0.41	0.26	0.65	**s6**	0.50	0.49	0.35	0.66

Eigenvector centrality

Older					Younger				
Sy	Sa	Bo	q2.5	q97.5	Sy	Sa	Bo	q2.5	q97.5
**s103**	0.44	0.39	0.28	0.46	**s103**	0.46	0.44	0.35	0.49
**s117**	0.32	0.33	0.21	0.44	**s117**	0.33	0.34	0.25	0.43
**s123**	0.26	0.30	0.16	0.40	**s57**	0.32	0.25	0.12	0.35
**s57**	0.28	0.24	0.16	0.31	**s97**	0.27	0.25	0.20	0.31
**s97**	0.25	0.22	0.15	0.28	**s134**	0.19	0.23	0.15	0.32
**s14**	0.19	0.22	0.13	0.31	**s123**	0.19	0.22	0.12	0.33
**s89**	0.16	0.20	0.08	0.35	**s136**	0.22	0.20	0.11	0.29
**s113**	0.15	0.18	0.08	0.30	**s17**	0.20	0.19	0.14	0.26
**s136**	0.23	0.17	0.08	0.30	**s89**	0.20	0.19	0.08	0.32
**s134**	0.17	0.17	0.11	0.24	**s60**	0.14	0.18	0.10	0.30
**s34**	0.16	0.17	0.08	0.32	**s40**	0.20	0.17	0.11	0.24
**s40**	0.20	0.16	0.09	0.25	**s14**	0.15	0.16	0.11	0.23
**s93**	0.11	0.16	0.07	0.30	**s77**	0.17	0.16	0.12	0.21
**s17**	0.19	0.15	0.09	0.21	**s93**	0.14	0.15	0.06	0.26
**s77**	0.17	0.15	0.10	0.20	**s20**	0.16	0.13	0.08	0.20
**s135**	0.09	0.14	0.07	0.28	**s135**	0.13	0.12	0.06	0.23
**s83**	0.14	0.13	0.08	0.19	**s113**	0.13	0.11	0.05	0.18
**s60**	0.10	0.13	0.08	0.25	**s83**	0.11	0.11	0.07	0.16
**s20**	0.17	0.13	0.08	0.21	**s34**	0.10	0.11	0.06	0.18
**s43**	0.12	0.10	0.05	0.16	**s23**	0.11	0.10	0.04	0.17
**s63**	0.06	0.10	0.05	0.16	**s131**	0.10	0.10	0.04	0.15
**s129**	0.06	0.09	0.05	0.17	**s129**	0.07	0.10	0.05	0.18
**s131**	0.12	0.09	0.05	0.14	**s98**	0.08	0.09	0.04	0.14
**s37**	0.11	0.09	0.06	0.13	**s37**	0.10	0.09	0.07	0.12
**s29**	0.06	0.09	0.06	0.13	**s3**	0.09	0.08	0.05	0.11
**s23**	0.07	0.08	0.04	0.15	**s43**	0.10	0.08	0.03	0.13
**s3**	0.09	0.08	0.05	0.11	**s109**	0.08	0.08	0.04	0.12
**s49**	0.08	0.08	0.03	0.12	**s63**	0.07	0.08	0.03	0.14
**s9**	0.10	0.07	0.04	0.11	**s29**	0.05	0.07	0.04	0.11
**s107**	0.09	0.07	0.02	0.11	**s49**	0.04	0.07	0.03	0.11
**s109**	0.04	0.07	0.04	0.11	**s107**	0.04	0.07	0.02	0.10
**s98**	0.07	0.07	0.03	0.10	**s9**	0.05	0.06	0.03	0.09
**s132**	0.06	0.07	0.02	0.10	**s132**	0.06	0.05	0.02	0.10
**s114**	0.07	0.06	0.03	0.09	**s31**	0.05	0.05	0.01	0.10
**s54**	0.07	0.05	0.02	0.08	**s73**	0.06	0.05	0.02	0.09
**s80**	0.04	0.05	0.02	0.08	**s114**	0.07	0.05	0.02	0.08
**s73**	0.03	0.05	0.02	0.08	**s54**	0.04	0.05	0.02	0.08
**s31**	0.04	0.04	0.01	0.06	**s69**	0.02	0.05	0.01	0.09
**s69**	0.03	0.03	0.01	0.07	**s74**	0.04	0.04	0.01	0.09
**s11**	0.04	0.03	0.01	0.07	**s80**	0.05	0.04	0.02	0.07
**s86**	0.02	0.03	0.01	0.05	**s86**	0.02	0.03	0.01	0.05
**s74**	0.02	0.03	0.01	0.06	**s11**	0.01	0.03	0.01	0.06
**s6**	0.02	0.02	0.01	0.04	**s59**	0.01	0.02	0.01	0.04
**s59**	0.01	0.01	0.00	0.03	**s6**	0.01	0.02	0.01	0.03

Bridge strength centrality

Older					Younger				
Sy	Sa	Bo	q2.5	q97.5	Sy	Sa	Bo	q2.5	q97.5
**s103**	3.24	2.58	0.99	4.06	**s103**	3.70	3.30	1.73	4.92
**s117**	1.15	1.77	0.53	3.47	**s117**	2.22	1.80	0.52	3.46
**s123**	1.04	1.44	0.00	2.83	**s134**	0.68	0.94	0.00	2.14
**s14**	0.29	0.68	0.00	1.67	**s136**	0.88	0.85	0.00	2.06
**s136**	1.34	0.54	0.00	1.87	**s123**	0.87	0.81	0.00	2.25
**s134**	0.52	0.50	0.00	1.11	**s57**	1.80	0.59	0.00	1.56
**s89**	0.00	0.49	0.00	1.89	**s60**	0.26	0.59	0.00	1.96
**s34**	0.26	0.44	0.00	1.84	**s89**	0.25	0.51	0.00	1.92
**s93**	0.00	0.42	0.00	1.71	**s40**	0.97	0.49	0.00	1.21
**s40**	0.89	0.41	0.00	1.15	**s97**	0.68	0.41	0.00	0.95
**s57**	0.58	0.38	0.00	0.99	**s14**	1.13	0.39	0.00	1.23
**s113**	0.00	0.37	0.00	1.32	**s20**	0.28	0.29	0.00	0.93
**s135**	0.00	0.35	0.00	1.60	**s93**	0.00	0.29	0.00	1.28
**s60**	0.00	0.30	0.00	1.10	**s135**	0.55	0.27	0.00	1.14
**s83**	0.32	0.26	0.00	0.68	**s131**	0.00	0.23	0.00	0.73
**s20**	0.60	0.24	0.00	0.85	**s83**	0.55	0.21	0.00	0.66
**s97**	0.23	0.20	0.00	0.59	**s98**	0.00	0.19	0.00	0.69
**s131**	0.45	0.20	0.00	0.67	**s34**	0.28	0.17	0.00	0.79
**s107**	0.37	0.16	0.00	0.47	**s3**	0.26	0.15	0.00	0.49
**s29**	0.00	0.15	0.00	0.55	**s23**	0.00	0.14	0.00	0.62
**s43**	0.23	0.14	0.00	0.56	**s113**	0.00	0.13	0.00	0.56
**s3**	0.22	0.11	0.00	0.46	**s107**	0.00	0.13	0.00	0.43
**s132**	0.19	0.10	0.00	0.43	**s17**	0.32	0.12	0.00	0.56
**s63**	0.00	0.10	0.00	0.51	**s49**	0.00	0.10	0.00	0.45
**s23**	0.00	0.09	0.00	0.56	**s129**	0.00	0.10	0.00	0.57
**s98**	0.00	0.08	0.00	0.41	**s109**	0.24	0.09	0.00	0.48
**s49**	0.00	0.07	0.00	0.41	**s29**	0.00	0.09	0.00	0.49
**s129**	0.00	0.07	0.00	0.56	**s43**	0.00	0.08	0.00	0.29
**s9**	0.20	0.07	0.00	0.36	**s132**	0.00	0.07	0.00	0.43
**s109**	0.00	0.06	0.00	0.30	**s69**	0.00	0.07	0.00	0.41
**s54**	0.18	0.05	0.00	0.26	**s9**	0.16	0.07	0.00	0.35
**s11**	0.00	0.04	0.00	0.21	**s31**	0.00	0.05	0.00	0.25
**s114**	0.00	0.03	0.00	0.25	**s73**	0.00	0.05	0.00	0.26
**s69**	0.00	0.03	0.00	0.23	**s74**	0.00	0.05	0.00	0.24
**s17**	0.00	0.03	0.00	0.32	**s63**	0.00	0.05	0.00	0.30
**s86**	0.00	0.03	0.00	0.23	**s86**	0.00	0.04	0.00	0.26
**s6**	0.00	0.03	0.00	0.20	**s114**	0.00	0.04	0.00	0.26
**s80**	0.00	0.03	0.00	0.26	**s54**	0.18	0.04	0.00	0.29
**s74**	0.00	0.02	0.00	0.19	**s80**	0.00	0.03	0.00	0.22
**s73**	0.00	0.02	0.00	0.23	**s77**	0.00	0.02	0.00	0.29
**s77**	0.00	0.02	0.00	0.24	**s11**	0.00	0.02	0.00	0.20
**s31**	0.00	0.02	0.00	0.18	**s6**	0.00	0.02	0.00	0.12
**s59**	0.00	0.01	0.00	0.11	**s59**	0.00	0.01	0.00	0.15
**s37**	0.00	0.00	0.00	0.00	**s37**	0.00	0.00	0.00	0.00

Hybrid centrality

Older					Younger				
Sy	Sa	Bo	q2.5	q97.5	Sy	Sa	Bo	q2.5	q97.5
**s103**	1.00	0.99	0.93	1.00	**s103**	1.00	1.00	0.97	1.00
**s117**	0.97	0.96	0.88	1.00	**s117**	0.98	0.96	0.88	1.00
**s123**	0.94	0.95	0.81	1.00	**s123**	0.88	0.89	0.68	0.98
**s14**	0.88	0.86	0.66	0.95	**s136**	0.95	0.88	0.67	0.97
**s57**	0.90	0.83	0.70	0.91	**s134**	0.82	0.87	0.63	0.97
**s89**	0.78	0.82	0.42	0.98	**s57**	0.93	0.81	0.40	0.95
**s136**	0.95	0.80	0.50	0.97	**s97**	0.83	0.81	0.71	0.90
**s113**	0.73	0.77	0.40	0.94	**s60**	0.65	0.81	0.50	0.97
**s97**	0.80	0.75	0.63	0.85	**s89**	0.85	0.80	0.41	0.97
**s134**	0.64	0.74	0.46	0.90	**s14**	0.86	0.78	0.54	0.92
**s34**	0.76	0.72	0.39	0.97	**s93**	0.70	0.69	0.31	0.93
**s93**	0.56	0.71	0.40	0.96	**s40**	0.75	0.68	0.40	0.88
**s40**	0.80	0.67	0.36	0.89	**s98**	0.61	0.62	0.29	0.84
**s135**	0.46	0.67	0.35	0.95	**s17**	0.58	0.61	0.46	0.82
**s83**	0.66	0.63	0.39	0.85	**s135**	0.60	0.61	0.28	0.91
**s60**	0.47	0.60	0.35	0.91	**s131**	0.76	0.59	0.24	0.84
**s63**	0.44	0.58	0.29	0.83	**s20**	0.65	0.59	0.34	0.84
**s43**	0.69	0.58	0.32	0.81	**s83**	0.57	0.57	0.33	0.81
**s20**	0.75	0.54	0.32	0.83	**s34**	0.53	0.56	0.29	0.84
**s131**	0.71	0.54	0.23	0.82	**s23**	0.59	0.55	0.26	0.81
**s17**	0.53	0.50	0.37	0.66	**s113**	0.61	0.53	0.20	0.79
**s132**	0.43	0.49	0.19	0.72	**s77**	0.48	0.49	0.37	0.67
**s23**	0.50	0.49	0.20	0.79	**s31**	0.49	0.47	0.22	0.70
**s107**	0.72	0.48	0.17	0.76	**s63**	0.42	0.45	0.15	0.77
**s77**	0.47	0.48	0.39	0.61	**s129**	0.21	0.45	0.19	0.80
**s49**	0.44	0.45	0.17	0.74	**s49**	0.29	0.44	0.18	0.73
**s129**	0.29	0.44	0.20	0.82	**s43**	0.55	0.43	0.14	0.72
**s29**	0.35	0.43	0.24	0.68	**s29**	0.38	0.41	0.16	0.64
**s9**	0.56	0.42	0.20	0.62	**s109**	0.35	0.41	0.16	0.69
**s3**	0.47	0.38	0.19	0.62	**s132**	0.58	0.41	0.11	0.71
**s98**	0.31	0.38	0.15	0.69	**s3**	0.39	0.39	0.23	0.61
**s31**	0.43	0.35	0.13	0.56	**s9**	0.34	0.34	0.14	0.52
**s109**	0.20	0.35	0.17	0.62	**s74**	0.37	0.33	0.08	0.62
**s54**	0.43	0.28	0.10	0.48	**s107**	0.20	0.31	0.13	0.58
**s73**	0.12	0.26	0.11	0.53	**s114**	0.36	0.28	0.09	0.48
**s114**	0.28	0.25	0.11	0.47	**s69**	0.11	0.28	0.07	0.59
**s37**	0.27	0.25	0.19	0.32	**s86**	0.20	0.26	0.09	0.39
**s11**	0.26	0.20	0.06	0.46	**s37**	0.26	0.25	0.18	0.36
**s80**	0.13	0.20	0.08	0.34	**s73**	0.27	0.24	0.07	0.43
**s74**	0.09	0.18	0.07	0.42	**s54**	0.21	0.24	0.10	0.47
**s86**	0.04	0.18	0.05	0.36	**s80**	0.20	0.18	0.06	0.38
**s69**	0.16	0.18	0.06	0.38	**s11**	0.05	0.17	0.04	0.40
**s6**	0.13	0.16	0.04	0.32	**s6**	0.07	0.11	0.04	0.22
**s59**	0.06	0.07	0.04	0.19	**s59**	0.06	0.10	0.04	0.23

Sy – symptom (node) identifier, Sa – score of the sample, Bo – mean of the bootstrap, q2.5 – 2.5th percentile, q97.5 – 97.5th percentile

Note: raw scores are used instead of z-scores to avoid inflation of dissimilarities between centrality indices.

Comparison 3. Patients Diagnosed in 1980-2000 versus Patients Diagnosed in 2000-2015 Somatoform Symptoms Networks

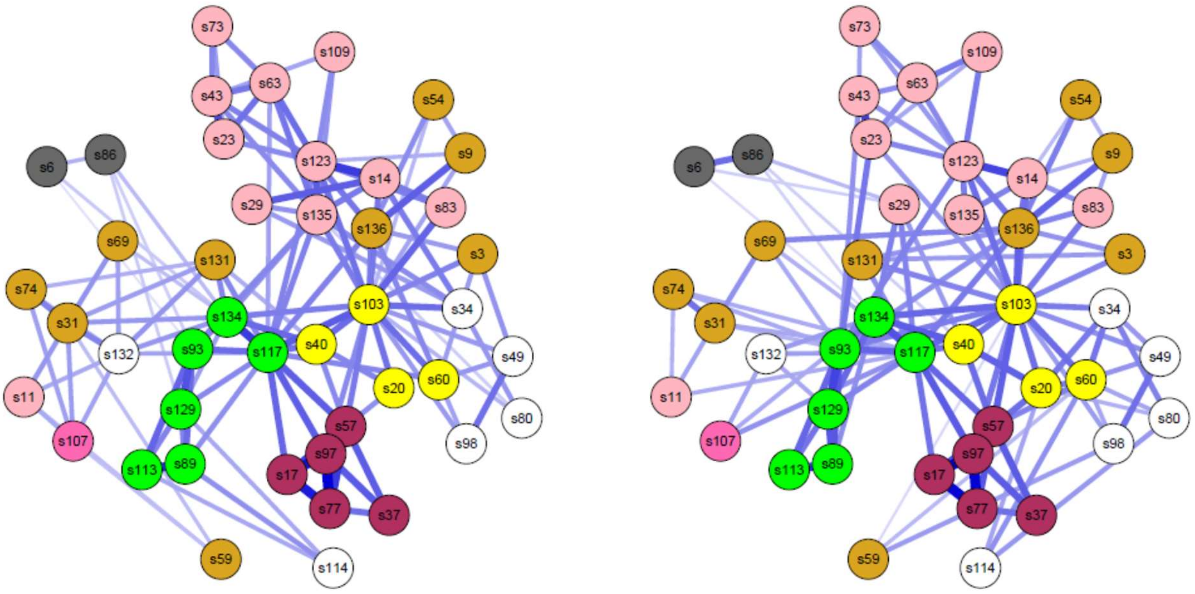

Supplementary Fig. SF13A. Plot with average layout aimed to compare edges; Patients diagnosed in **1980-2000** (left panel) versus **2000-2015** (right panel).

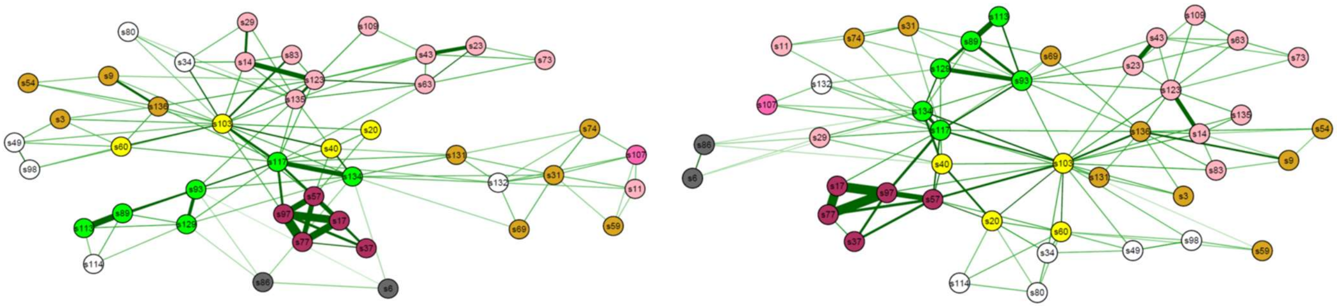

Supplementary Fig. SF13B. Sample network colored plots; Patients diagnosed in **1980-2000** (left panel) versus **2000-2015** (right panel).

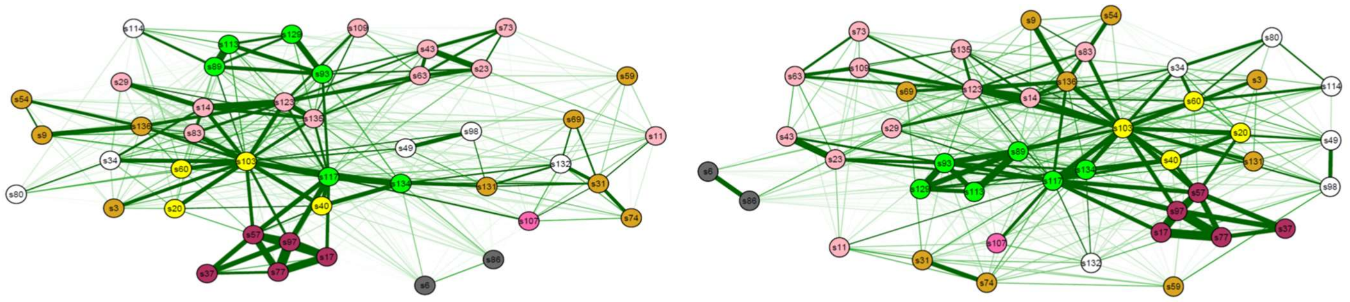

Supplementary Fig. SF13C. Mean bootnet plots (nonparametric bootstrap; nBoots = 20,000); Patients diagnosed in **1980-2000** (left panel) versus **2000-2015** (right panel).

s3 indicates choking/"lump"; s6, fatigue in the morning; s9, vomiting in stress; s11, itching or rashes; s14, dizziness; s17, discovering serious diseases; s20, palpitations; s23, loss of sensitivity in parts of the body; s29, persistent headaches; s31, flatulence or involuntary passing of gas; s34, flushes of blood into the head; s37, ritualistic actions to avoid disease; s40, heart pain; s43, temporary paralyses; s49, dry mouth; s54, loss of appetite; s57, focusing on body functions – e.g., pulse; s59, attacks of hunger – e.g., at night; s60, heat or cold w. reasons; s63, periodic blindness or deafness; s69, diarrhea; s73, transient aphonia; s74, constipation; s77, fears about health and contracting diseases; s80, blushing; s83, faintness; s86, constant fatigue; s89, trembling of legs, hands…; s93, muscle cramps; s97, feelings of having serious diseases; s98, excessive thirst; s103, dyspnea; s107, pains in the sexual organs; s109, hypersensitivity; s113, trembling of the face, eyelids, head…; s114, excessive perspiration; s117, undefined "travelling" pains; s123, disorders of balance; s129, muscle tensions; s131, heartburn; s132, passing urine frequently; s134, muscle pains – e.g., in the back; s135, buzzing in the ears; s136, nausea.

Comparison 3. continued

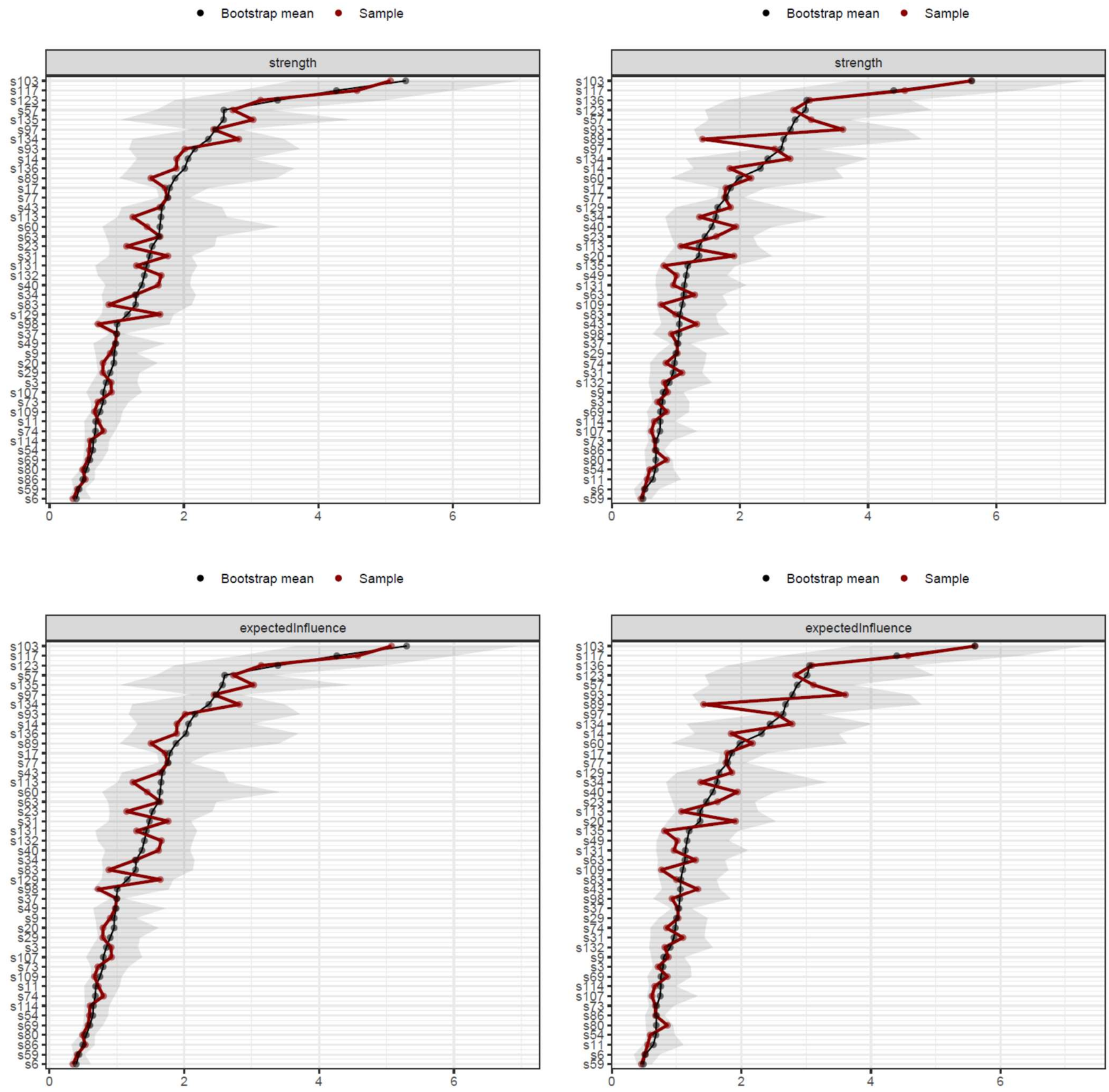

Supplementary Fig. SF14. Bootstrapped (nBoots = 20,000) confidence intervals of estimated node centrality for the network of 44 somatoform symptoms in patients diagnosed in 1980–2000 vs. 2000–2015.

UPPER LEFT PANEL: strength; 1980-2000.
LOWER LEFT PANEL: expected influence; 1980-2000.

UPPER RIGHT PANEL: strength; 2000-2015.
LOWER RIGHT PANEL: expected influence; 2000-2015.

The *red line* indicates the sample values and the *gray area* the bootstrapped CIs. Each *horizontal line* represents one node of the network. The order is set from the strongest to the weakest node (according to bootstrap means). The mean of the bootstrap samples (*black line*) was used in ordering the edges. The *y*-axis labels are raw scores**.**

Comparison 3. continued

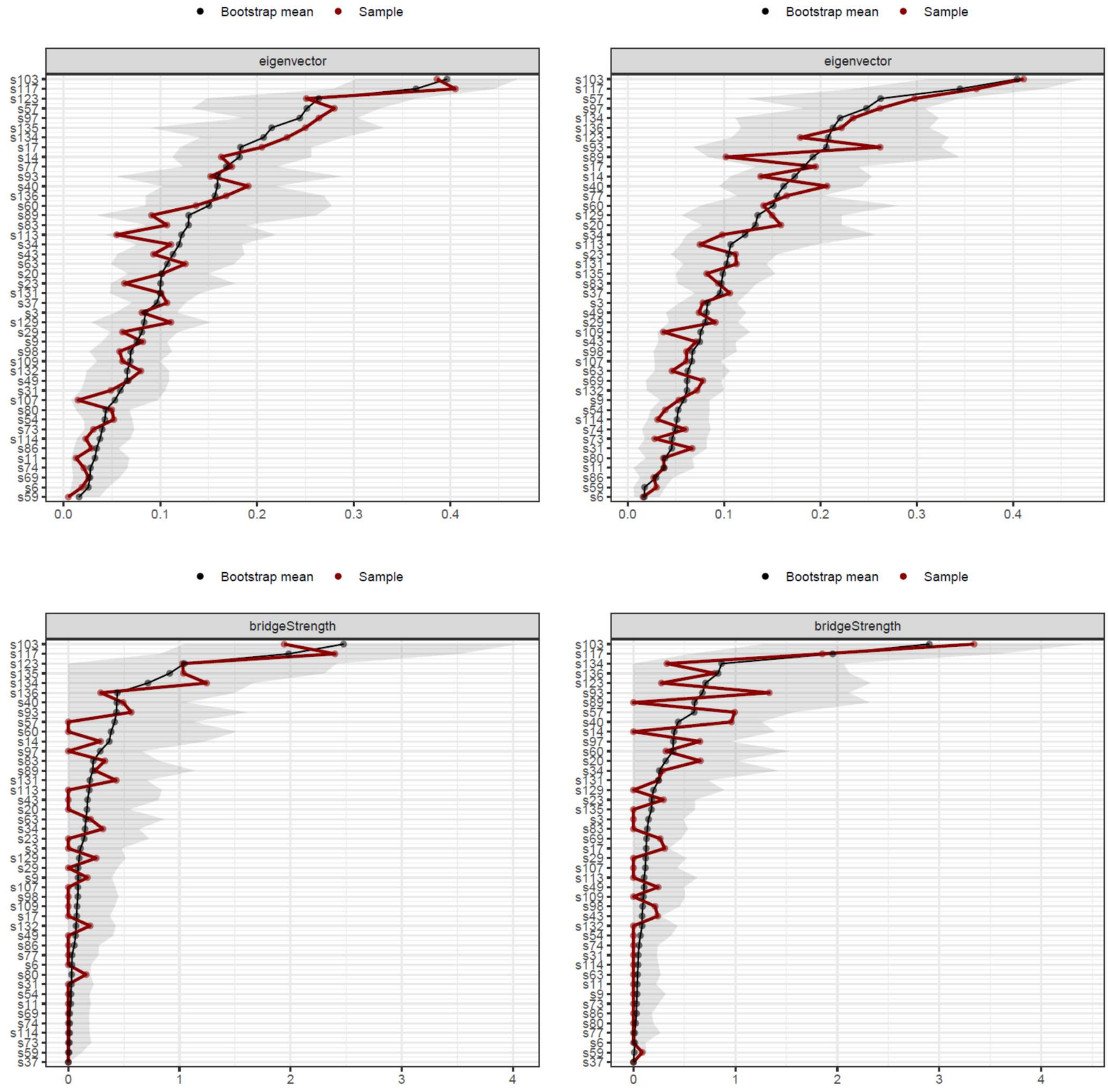

Supplementary Fig. SF15. Bootstrapped (nBoots = 20,000) confidence intervals of estimated node centrality for the network of 44 somatoform symptoms in patients diagnosed in 1980-2000 vs. 2000-2015.

UPPER LEFT PANEL: eigenvector; in **1980–2000**.
LOWER LEFT PANEL: bridge strength; in **1980–2000**.

UPPER RIGHT PANEL: eigenvector; in **2000–2015**.
LOWER RIGHT PANEL: bridge strength; **2000–2015**.

The *red line* indicates the sample values and the *gray area* the bootstrapped CIs. Each *horizontal line* represents one node of the network. The order is set from the strongest to the weakest node (according to bootstrap means). The mean of the bootstrap samples (*black line*) was used in ordering the edges. The *y*-axis labels are raw scores**.**

Comparison 3. continued

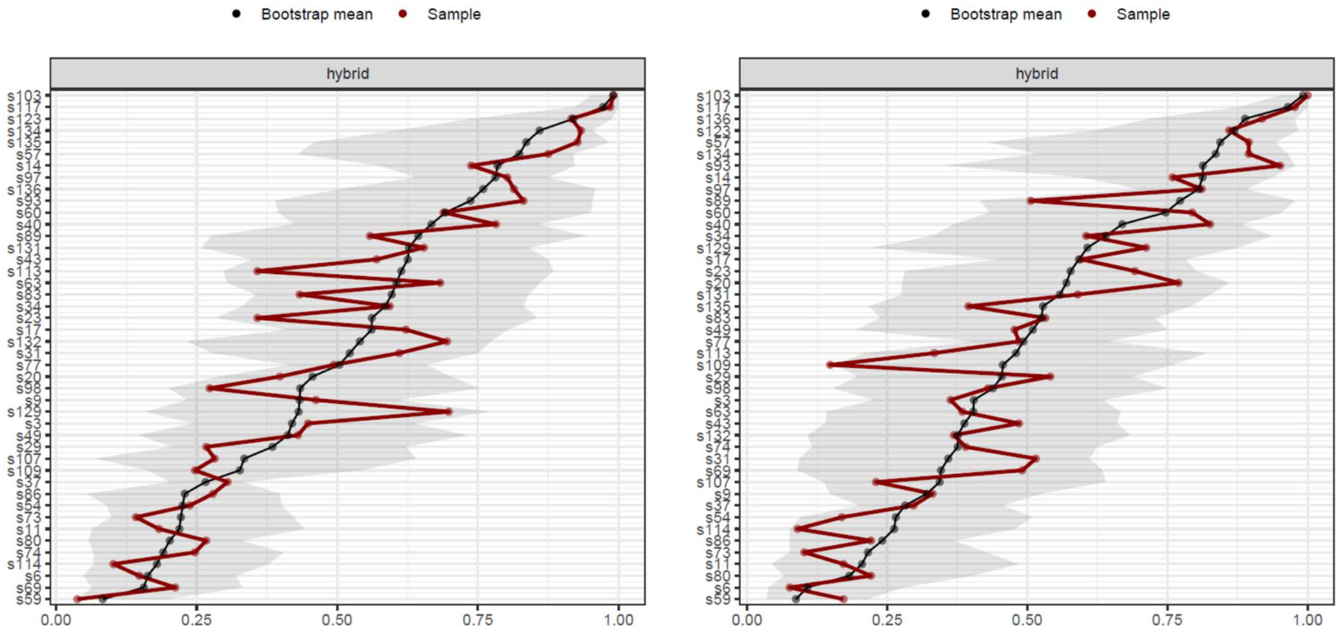

Supplementary Fig. SF16. Bootstrapped (nBoots = 20,000) confidence intervals of estimated node **hybrid** centrality for the network of 44 somatoform symptoms in patients diagnosed in 1980–2000 vs. 2000–2015.

LEFT PANEL: 1980–2000. **RIGHT PANEL: 2000–2015**.

Supplementary Table ST10. Comparison 3. Twenty edges in the network with the highest mean weights (raw scores): sample and bootstrap estimates

Comparison 3 and edges									
1980-2000					2000-2015				
**id**	**Sa**	**Bo**	**q2.5**	**q97.5**	**id**	**Sa**	**Bo**	**q2.5**	**q97.5**
**s77--s97**	0.54	0.54	0.51	0.58	**s17--s97**	0.55	0.55	0.52	0.59
**s17--s97**	0.51	0.51	0.47	0.54	**s77--s97**	0.55	0.55	0.51	0.58
**s17--s77**	0.48	0.48	0.44	0.53	**s17--s77**	0.54	0.54	0.50	0.58
**s89--s113**	0.46	0.46	0.42	0.50	**s89--s113**	0.44	0.44	0.40	0.48
**s57--s97**	0.45	0.45	0.41	0.49	**s57--s97**	0.44	0.44	0.40	0.48
**s14--s123**	0.42	0.42	0.38	0.46	**s23--s43**	0.41	0.41	0.36	0.46
**s57--s77**	0.42	0.42	0.37	0.46	**s93--s129**	0.41	0.41	0.37	0.45
**s117--s134**	0.41	0.41	0.37	0.45	**s14--s123**	0.40	0.40	0.36	0.44
**s17--s57**	0.40	0.40	0.36	0.45	**s17--s57**	0.38	0.38	0.34	0.42
**s23--s43**	0.38	0.38	0.33	0.43	**s117--s134**	0.37	0.37	0.33	0.41
**s93--s129**	0.37	0.36	0.32	0.41	**s89--s93**	0.37	0.36	0.32	0.41
**s123--s135**	0.35	0.35	0.30	0.39	**s57--s77**	0.36	0.36	0.32	0.40
**s103--s117**	0.34	0.34	0.30	0.39	**s89--s129**	0.36	0.36	0.00	0.41
**s37--s97**	0.35	0.34	0.29	0.39	**s37--s97**	0.35	0.34	0.00	0.40
**s97--s117**	0.34	0.34	0.30	0.38	**s9--s136**	0.34	0.34	0.30	0.38
**s9--s136**	0.34	0.33	0.00	0.38	**s103--s136**	0.34	0.34	0.30	0.38
**s14--s29**	0.34	0.33	0.00	0.39	**s37--s57**	0.35	0.34	0.00	0.40
**s37--s57**	0.34	0.32	0.00	0.39	**s97--s117**	0.36	0.33	0.00	0.40
**s83--s103**	0.33	0.32	0.26	0.37	**s103--s117**	0.33	0.33	0.29	0.37
**s57--s117**	0.34	0.32	0.00	0.38	**s93--s113**	0.33	0.33	0.28	0.38

id – edge identifier (with codes of the two nodes), Sa – score of the sample, Bo – mean of the bootstrap, q2.5 – 2.5th percentile, q97.5 – 97.5th percentile

Supplementary Table ST11. Comparison 3: sample raw scores and bootstrap estimated means of five centralities

Strength centrality

1980-2000					2000-2015				
Sy	Sa	Bo	q2.5	q97.5	Sy	Sa	Bo	q2.5	q97.5
**s103**	5.07	5.29	3.61	6.97	**s103**	5.61	5.61	3.69	7.32
**s117**	4.58	4.27	2.82	5.98	**s117**	4.57	4.41	2.62	6.28
**s123**	3.14	3.40	1.88	4.98	**s136**	3.09	3.06	1.79	4.64
**s57**	2.73	2.60	1.59	3.30	**s123**	2.83	3.02	1.46	4.98
**s135**	3.03	2.57	1.07	4.48	**s57**	3.11	2.87	1.52	3.71
**s97**	2.44	2.47	2.11	2.83	**s93**	3.61	2.78	1.26	4.59
**s134**	2.82	2.37	1.23	3.52	**s89**	1.42	2.67	1.36	4.77
**s93**	2.01	2.16	1.18	3.70	**s97**	2.55	2.64	2.28	3.20
**s14**	1.89	2.06	1.32	2.98	**s134**	2.78	2.44	1.17	4.03
**s136**	1.89	2.02	1.22	3.69	**s14**	1.84	2.31	1.28	3.49
**s89**	1.51	1.87	1.04	3.40	**s60**	2.17	1.97	0.92	3.60
**s17**	1.72	1.79	1.59	2.36	**s17**	1.78	1.85	1.67	2.39
**s77**	1.75	1.76	1.63	2.08	**s77**	1.76	1.79	1.65	2.06
**s43**	1.64	1.67	1.08	2.57	**s129**	1.85	1.65	1.00	2.68
**s113**	1.24	1.66	1.02	2.63	**s34**	1.37	1.62	0.84	3.37
**s60**	1.45	1.64	0.78	3.39	**s40**	1.94	1.57	0.88	2.51
**s63**	1.65	1.62	0.87	2.50	**s23**	1.63	1.45	0.93	2.20
**s23**	1.15	1.52	0.90	2.44	**s20**	1.91	1.37	0.87	2.52
**s31**	1.76	1.49	0.89	2.11	**s113**	1.07	1.36	0.99	2.28
**s131**	1.29	1.44	0.68	2.20	**s135**	0.81	1.19	0.75	1.97
**s132**	1.66	1.41	0.70	2.12	**s49**	1.01	1.16	0.69	1.79
**s40**	1.62	1.37	0.84	2.09	**s131**	0.96	1.13	0.69	2.08
**s34**	1.27	1.29	0.78	2.16	**s63**	1.29	1.12	0.71	1.72
**s83**	0.88	1.28	0.80	2.09	**s109**	0.77	1.10	0.69	1.80
**s129**	1.65	1.15	0.78	1.85	**s83**	1.00	1.06	0.74	1.63
**s98**	0.72	1.00	0.64	1.77	**s43**	1.33	1.05	0.77	1.66
**s37**	1.00	1.00	0.90	1.10	**s98**	0.93	1.05	0.64	1.83
**s49**	0.98	0.99	0.65	1.69	**s37**	1.02	1.03	0.92	1.23
**s9**	0.90	0.96	0.68	1.28	**s29**	1.02	1.00	0.72	1.47
**s20**	0.80	0.95	0.71	1.59	**s74**	0.84	0.98	0.60	1.45
**s29**	0.79	0.90	0.72	1.35	**s31**	1.10	0.96	0.60	1.42
**s3**	0.92	0.84	0.63	1.30	**s132**	0.82	0.90	0.58	1.54
**s107**	0.92	0.80	0.55	1.36	**s9**	0.87	0.80	0.61	1.12
**s73**	0.72	0.80	0.62	1.17	**s3**	0.71	0.79	0.64	1.21
**s109**	0.67	0.75	0.62	1.07	**s69**	0.86	0.76	0.55	1.23
**s11**	0.73	0.69	0.52	1.04	**s114**	0.66	0.76	0.58	0.98
**s74**	0.80	0.68	0.52	0.96	**s107**	0.62	0.75	0.54	1.32
**s114**	0.60	0.65	0.52	0.89	**s73**	0.67	0.69	0.58	0.95
**s54**	0.59	0.64	0.51	0.87	**s86**	0.68	0.69	0.51	0.81
**s69**	0.57	0.60	0.50	0.79	**s80**	0.86	0.69	0.52	0.95
**s80**	0.49	0.55	0.45	0.67	**s54**	0.59	0.68	0.54	0.95
**s86**	0.53	0.50	0.33	0.67	**s11**	0.55	0.64	0.48	1.09
**s59**	0.41	0.44	0.36	0.53	**s6**	0.51	0.52	0.35	0.73
**s6**	0.35	0.40	0.28	0.61	**s59**	0.46	0.49	0.39	0.62

Expected influence centrality

1980-2000					2000-2015				
Sy	Sa	Bo	q2.5	q97.5	Sy	Sa	Bo	q2.5	q97.5
**s103**	5.07	5.30	3.64	6.96	**s103**	5.61	5.61	3.73	7.36
**s117**	4.58	4.27	2.81	5.95	**s117**	4.57	4.40	2.60	6.32
**s123**	3.14	3.40	1.89	4.98	**s136**	3.09	3.05	1.80	4.62
**s57**	2.73	2.60	1.59	3.28	**s123**	2.83	3.02	1.46	4.99
**s135**	3.03	2.57	1.07	4.47	**s57**	3.11	2.87	1.52	3.71
**s97**	2.44	2.47	2.11	2.83	**s93**	3.61	2.79	1.28	4.61
**s134**	2.82	2.36	1.23	3.47	**s89**	1.42	2.67	1.36	4.77
**s93**	2.01	2.16	1.18	3.74	**s97**	2.55	2.64	2.30	3.19
**s14**	1.89	2.06	1.31	2.99	**s134**	2.78	2.44	1.17	4.02
**s136**	1.89	2.01	1.20	3.69	**s14**	1.84	2.32	1.29	3.50
**s89**	1.51	1.87	1.05	3.39	**s60**	2.17	1.98	0.92	3.62
**s17**	1.72	1.79	1.59	2.35	**s17**	1.78	1.85	1.67	2.39
**s77**	1.75	1.76	1.63	2.08	**s77**	1.76	1.79	1.64	2.07
**s43**	1.64	1.68	1.08	2.56	**s129**	1.85	1.65	1.00	2.67
**s113**	1.24	1.66	1.00	2.64	**s34**	1.37	1.62	0.84	3.33
**s60**	1.45	1.65	0.79	3.43	**s40**	1.94	1.56	0.88	2.52
**s63**	1.65	1.63	0.86	2.49	**s23**	1.63	1.46	0.94	2.20
**s23**	1.15	1.53	0.89	2.44	**s20**	1.91	1.37	0.87	2.52
**s31**	1.76	1.49	0.89	2.11	**s113**	1.07	1.35	0.99	2.26
**s131**	1.29	1.44	0.68	2.21	**s135**	0.81	1.19	0.75	1.95
**s132**	1.66	1.41	0.72	2.11	**s49**	1.01	1.16	0.69	1.79
**s40**	1.62	1.38	0.83	2.10	**s131**	0.96	1.14	0.69	2.09
**s34**	1.27	1.29	0.78	2.14	**s63**	1.29	1.12	0.71	1.72
**s83**	0.88	1.28	0.80	2.14	**s109**	0.77	1.09	0.70	1.80
**s129**	1.65	1.15	0.77	1.83	**s83**	1.00	1.06	0.74	1.62
**s98**	0.72	1.00	0.64	1.76	**s43**	1.33	1.06	0.77	1.67
**s37**	1.00	1.00	0.90	1.10	**s98**	0.93	1.05	0.64	1.83
**s49**	0.98	0.99	0.65	1.73	**s37**	1.02	1.03	0.92	1.23
**s9**	0.90	0.96	0.68	1.28	**s29**	1.02	1.00	0.72	1.48
**s20**	0.80	0.96	0.71	1.60	**s74**	0.84	0.98	0.60	1.46
**s29**	0.79	0.90	0.72	1.33	**s31**	1.10	0.96	0.60	1.42
**s3**	0.92	0.84	0.63	1.31	**s132**	0.82	0.90	0.58	1.55
**s107**	0.92	0.80	0.55	1.37	**s9**	0.87	0.80	0.61	1.11
**s73**	0.72	0.80	0.62	1.19	**s3**	0.71	0.79	0.64	1.19
**s109**	0.67	0.75	0.62	1.07	**s114**	0.66	0.76	0.58	0.99
**s11**	0.73	0.69	0.52	1.04	**s69**	0.86	0.76	0.55	1.22
**s74**	0.80	0.68	0.53	0.96	**s107**	0.62	0.75	0.54	1.32
**s114**	0.60	0.65	0.52	0.89	**s73**	0.67	0.69	0.58	0.95
**s54**	0.59	0.64	0.51	0.87	**s86**	0.68	0.69	0.52	0.82
**s69**	0.57	0.60	0.50	0.79	**s80**	0.86	0.68	0.52	0.94
**s80**	0.49	0.55	0.45	0.67	**s54**	0.59	0.68	0.54	0.97
**s86**	0.53	0.50	0.33	0.67	**s11**	0.55	0.64	0.49	1.08
**s59**	0.41	0.44	0.36	0.53	**s6**	0.51	0.52	0.34	0.74
**s6**	0.35	0.40	0.28	0.62	**s59**	0.46	0.49	0.39	0.62

Eigenvector centrality

1980-2000					2000-2015				
Sy	Sa	Bo	q2.5	q97.5	Sy	Sa	Bo	q2.5	q97.5
**s103**	0.39	0.40	0.30	0.47	**s103**	0.41	0.40	0.31	0.47
**s117**	0.41	0.36	0.28	0.44	**s117**	0.36	0.34	0.23	0.43
**s123**	0.25	0.26	0.15	0.37	**s57**	0.30	0.26	0.13	0.34
**s57**	0.28	0.25	0.14	0.33	**s97**	0.26	0.25	0.18	0.31
**s97**	0.26	0.24	0.19	0.30	**s134**	0.23	0.22	0.11	0.32
**s135**	0.25	0.21	0.09	0.33	**s136**	0.22	0.21	0.12	0.32
**s134**	0.23	0.21	0.14	0.29	**s123**	0.18	0.21	0.11	0.34
**s17**	0.21	0.18	0.12	0.25	**s93**	0.26	0.21	0.07	0.33
**s14**	0.16	0.18	0.11	0.26	**s89**	0.10	0.19	0.08	0.34
**s77**	0.17	0.17	0.13	0.22	**s17**	0.20	0.18	0.13	0.24
**s40**	0.19	0.16	0.10	0.22	**s14**	0.14	0.17	0.11	0.25
**s93**	0.15	0.16	0.06	0.29	**s40**	0.21	0.16	0.09	0.24
**s136**	0.17	0.16	0.09	0.27	**s77**	0.17	0.15	0.11	0.20
**s60**	0.14	0.15	0.09	0.28	**s60**	0.14	0.15	0.08	0.28
**s89**	0.09	0.13	0.03	0.27	**s129**	0.15	0.13	0.06	0.22
**s83**	0.11	0.13	0.09	0.19	**s20**	0.16	0.13	0.08	0.23
**s113**	0.06	0.12	0.04	0.22	**s34**	0.10	0.12	0.06	0.25
**s34**	0.11	0.12	0.08	0.18	**s113**	0.08	0.11	0.06	0.19
**s43**	0.09	0.11	0.06	0.19	**s23**	0.11	0.10	0.05	0.15
**s63**	0.13	0.11	0.05	0.18	**s131**	0.11	0.10	0.06	0.15
**s20**	0.10	0.10	0.07	0.15	**s135**	0.08	0.10	0.05	0.15
**s23**	0.06	0.10	0.05	0.18	**s83**	0.09	0.10	0.05	0.13
**s131**	0.10	0.10	0.05	0.14	**s37**	0.11	0.10	0.07	0.12
**s37**	0.11	0.10	0.07	0.13	**s3**	0.08	0.08	0.06	0.11
**s3**	0.08	0.08	0.05	0.12	**s49**	0.07	0.08	0.04	0.12
**s129**	0.11	0.08	0.03	0.15	**s29**	0.09	0.08	0.04	0.12
**s29**	0.06	0.08	0.05	0.12	**s109**	0.04	0.08	0.03	0.13
**s9**	0.08	0.08	0.04	0.11	**s43**	0.07	0.07	0.03	0.11
**s98**	0.06	0.07	0.03	0.11	**s98**	0.06	0.07	0.03	0.11
**s109**	0.06	0.07	0.04	0.10	**s107**	0.06	0.07	0.03	0.10
**s132**	0.08	0.07	0.02	0.11	**s63**	0.05	0.06	0.03	0.11
**s49**	0.07	0.07	0.02	0.11	**s69**	0.08	0.06	0.02	0.10
**s31**	0.05	0.06	0.02	0.11	**s132**	0.07	0.06	0.02	0.10
**s107**	0.02	0.05	0.01	0.10	**s9**	0.05	0.06	0.03	0.09
**s80**	0.05	0.04	0.01	0.07	**s54**	0.04	0.05	0.02	0.09
**s54**	0.05	0.04	0.02	0.07	**s114**	0.03	0.05	0.02	0.08
**s73**	0.03	0.04	0.02	0.07	**s74**	0.06	0.05	0.02	0.09
**s114**	0.02	0.04	0.01	0.07	**s31**	0.07	0.05	0.01	0.08
**s86**	0.03	0.03	0.01	0.06	**s73**	0.03	0.05	0.02	0.08
**s11**	0.01	0.03	0.01	0.07	**s80**	0.04	0.04	0.02	0.07
**s74**	0.02	0.03	0.01	0.07	**s11**	0.04	0.04	0.01	0.07
**s69**	0.03	0.03	0.01	0.06	**s86**	0.03	0.03	0.01	0.05
**s6**	0.02	0.03	0.01	0.05	**s59**	0.03	0.02	0.01	0.04
**s59**	0.01	0.02	0.01	0.04	**s6**	0.02	0.02	0.01	0.03

Bridge strength centrality

1980-2000					2000-2015				
Sy	Sa	Bo	q2.5	q97.5	Sy	Sa	Bo	q2.5	q97.5
**s103**	1.94	2.49	1.06	4.05	**s103**	3.34	2.92	1.31	4.45
**s117**	2.40	1.98	0.82	3.55	**s117**	1.85	1.95	0.55	3.56
**s123**	1.03	1.05	0.00	2.40	**s134**	0.33	0.86	0.00	2.03
**s135**	1.04	0.90	0.00	2.30	**s136**	0.80	0.83	0.00	2.09
**s134**	1.24	0.72	0.00	1.67	**s123**	0.27	0.71	0.00	2.31
**s136**	0.29	0.44	0.00	1.52	**s93**	1.33	0.68	0.00	2.15
**s40**	0.49	0.43	0.00	1.05	**s89**	0.00	0.60	0.00	2.31
**s93**	0.57	0.43	0.00	1.60	**s57**	0.99	0.59	0.00	1.55
**s57**	0.00	0.42	0.00	1.12	**s40**	0.96	0.43	0.00	1.24
**s60**	0.00	0.38	0.00	1.49	**s14**	0.00	0.40	0.00	1.37
**s14**	0.29	0.37	0.00	1.22	**s60**	0.32	0.40	0.00	1.56
**s97**	0.00	0.29	0.00	0.66	**s97**	0.65	0.39	0.00	0.93
**s83**	0.33	0.23	0.00	0.81	**s20**	0.66	0.32	0.00	1.00
**s89**	0.24	0.22	0.00	1.12	**s34**	0.29	0.26	0.00	1.41
**s131**	0.43	0.19	0.00	0.71	**s131**	0.24	0.25	0.00	0.72
**s113**	0.00	0.19	0.00	0.83	**s129**	0.00	0.19	0.00	0.90
**s43**	0.00	0.17	0.00	0.81	**s23**	0.29	0.18	0.00	0.60
**s20**	0.00	0.17	0.00	0.55	**s135**	0.00	0.17	0.00	0.60
**s63**	0.20	0.16	0.00	0.87	**s3**	0.00	0.15	0.00	0.49
**s34**	0.31	0.15	0.00	0.63	**s83**	0.00	0.14	0.00	0.53
**s23**	0.00	0.14	0.00	0.74	**s69**	0.26	0.13	0.00	0.47
**s3**	0.00	0.11	0.00	0.48	**s17**	0.31	0.12	0.00	0.34
**s129**	0.25	0.10	0.00	0.52	**s107**	0.00	0.12	0.00	0.43
**s9**	0.17	0.09	0.00	0.39	**s29**	0.00	0.12	0.00	0.52
**s29**	0.00	0.09	0.00	0.44	**s49**	0.24	0.11	0.00	0.50
**s98**	0.00	0.09	0.00	0.45	**s113**	0.00	0.10	0.00	0.59
**s107**	0.00	0.08	0.00	0.42	**s109**	0.00	0.10	0.00	0.51
**s109**	0.00	0.08	0.00	0.42	**s98**	0.21	0.09	0.00	0.44
**s17**	0.00	0.08	0.00	0.38	**s132**	0.00	0.09	0.00	0.43
**s132**	0.20	0.07	0.00	0.42	**s43**	0.24	0.09	0.00	0.27
**s49**	0.00	0.06	0.00	0.28	**s54**	0.00	0.07	0.00	0.37
**s86**	0.00	0.05	0.00	0.28	**s74**	0.00	0.05	0.00	0.25
**s6**	0.00	0.03	0.00	0.20	**s31**	0.00	0.05	0.00	0.24
**s77**	0.00	0.03	0.00	0.27	**s114**	0.00	0.05	0.00	0.25
**s80**	0.16	0.03	0.00	0.20	**s63**	0.00	0.04	0.00	0.26
**s31**	0.00	0.03	0.00	0.23	**s9**	0.00	0.04	0.00	0.31
**s54**	0.00	0.02	0.00	0.19	**s11**	0.00	0.03	0.00	0.22
**s11**	0.00	0.02	0.00	0.20	**s73**	0.00	0.03	0.00	0.24
**s69**	0.00	0.01	0.00	0.19	**s86**	0.00	0.03	0.00	0.20
**s74**	0.00	0.01	0.00	0.19	**s80**	0.00	0.02	0.00	0.20
**s114**	0.00	0.01	0.00	0.19	**s6**	0.00	0.01	0.00	0.11
**s73**	0.00	0.01	0.00	0.21	**s77**	0.00	0.01	0.00	0.26
**s59**	0.00	0.01	0.00	0.13	**s59**	0.09	0.01	0.00	0.13
**s37**	0.00	0.00	0.00	0.00	**s37**	0.00	0.00	0.00	0.00

Hybrid centrality

1980-2000					2000-2015				
Sy	Sa	Bo	q2.5	q97.5	Sy	Sa	Bo	q2.5	q97.5
**s103**	0.99	0.99	0.95	1.00	**s103**	1.00	0.99	0.95	1.00
**s117**	0.99	0.97	0.92	1.00	**s117**	0.98	0.96	0.88	1.00
**s123**	0.92	0.92	0.74	0.99	**s136**	0.92	0.89	0.72	0.98
**s134**	0.93	0.86	0.61	0.95	**s123**	0.86	0.87	0.62	0.98
**s135**	0.93	0.84	0.46	0.98	**s57**	0.90	0.84	0.43	0.94
**s57**	0.88	0.82	0.43	0.92	**s134**	0.90	0.84	0.52	0.97
**s14**	0.74	0.78	0.58	0.92	**s93**	0.95	0.81	0.36	0.98
**s97**	0.80	0.78	0.64	0.88	**s14**	0.76	0.81	0.60	0.94
**s136**	0.81	0.76	0.51	0.96	**s97**	0.81	0.81	0.70	0.89
**s93**	0.83	0.74	0.39	0.95	**s89**	0.51	0.77	0.42	0.98
**s60**	0.69	0.69	0.40	0.95	**s60**	0.79	0.75	0.43	0.95
**s40**	0.78	0.67	0.42	0.86	**s40**	0.83	0.67	0.37	0.88
**s89**	0.56	0.64	0.28	0.94	**s34**	0.61	0.64	0.33	0.94
**s131**	0.65	0.63	0.26	0.84	**s129**	0.71	0.61	0.22	0.87
**s43**	0.57	0.63	0.35	0.87	**s17**	0.60	0.59	0.45	0.78
**s113**	0.36	0.61	0.30	0.88	**s23**	0.69	0.58	0.28	0.80
**s63**	0.68	0.61	0.31	0.86	**s20**	0.77	0.57	0.28	0.86
**s83**	0.43	0.60	0.36	0.84	**s131**	0.59	0.56	0.28	0.83
**s34**	0.59	0.58	0.33	0.83	**s135**	0.40	0.53	0.22	0.79
**s23**	0.36	0.56	0.29	0.86	**s83**	0.53	0.52	0.24	0.73
**s17**	0.62	0.56	0.41	0.80	**s49**	0.48	0.51	0.20	0.75
**s132**	0.70	0.54	0.23	0.77	**s77**	0.49	0.49	0.41	0.64
**s31**	0.61	0.52	0.28	0.75	**s113**	0.33	0.48	0.21	0.82
**s77**	0.49	0.50	0.42	0.66	**s109**	0.15	0.46	0.16	0.76
**s20**	0.40	0.46	0.29	0.70	**s29**	0.54	0.45	0.20	0.69
**s98**	0.27	0.43	0.20	0.75	**s98**	0.43	0.44	0.15	0.75
**s9**	0.46	0.43	0.23	0.61	**s3**	0.36	0.40	0.23	0.63
**s129**	0.70	0.43	0.16	0.77	**s63**	0.38	0.40	0.14	0.67
**s3**	0.45	0.42	0.24	0.64	**s43**	0.49	0.39	0.14	0.66
**s49**	0.43	0.41	0.15	0.73	**s132**	0.37	0.38	0.11	0.68
**s29**	0.27	0.38	0.21	0.62	**s74**	0.39	0.38	0.12	0.63
**s107**	0.28	0.33	0.07	0.64	**s31**	0.52	0.36	0.09	0.61
**s109**	0.25	0.33	0.17	0.54	**s69**	0.49	0.35	0.09	0.63
**s37**	0.31	0.27	0.20	0.33	**s107**	0.23	0.34	0.14	0.64
**s86**	0.28	0.23	0.06	0.40	**s9**	0.33	0.32	0.15	0.52
**s54**	0.24	0.23	0.09	0.40	**s37**	0.30	0.28	0.20	0.46
**s73**	0.14	0.22	0.10	0.42	**s54**	0.17	0.27	0.10	0.51
**s11**	0.18	0.22	0.06	0.44	**s114**	0.09	0.26	0.08	0.48
**s80**	0.27	0.20	0.06	0.33	**s86**	0.22	0.24	0.08	0.37
**s74**	0.25	0.19	0.07	0.40	**s73**	0.10	0.22	0.08	0.43
**s114**	0.10	0.18	0.07	0.38	**s11**	0.17	0.21	0.04	0.49
**s6**	0.15	0.16	0.05	0.32	**s80**	0.22	0.18	0.07	0.40
**s69**	0.21	0.16	0.06	0.33	**s6**	0.08	0.11	0.04	0.28
**s59**	0.04	0.08	0.04	0.20	**s59**	0.17	0.09	0.04	0.22

Sy – symptom (node) identifier, Sa – score of the sample, Bo – mean of the bootstrap, q2.5 – 2.5th percentile, q97.5 – 97.5th percentile
