# Supplementary material for: Network analysis of body-related complaints in patients with neurotic or personality disorders referred to psychotherapy
Source: Heliyon. 2023 Feb 24;9(3):e14078. doi: 10.1016/j.heliyon.2023.e14078 (PMC10018473; doi:10.1016/j.heliyon.2023.e14078)
Supplement: Multimedia component 2 [file mmc2.pdf]

APPENDIX

DEPARTMENT OF PSYCHOTHERAPY
COLLEGIUM MEDICUM
JAGIELLONIAN UNIVERSITY
CRACOW, POLAND

Identification ....

SYMPTOM CHECKLIST “O”

Name: .... age: .... date of completion: ....

Profession: .... education: ....

address: ....

These items concern symptoms and difficulties that sometimes occur in neurotic disorders. Please read every item carefully and circle the answer that best indicates the intensity of your symptoms during the last week. Please use this key:

0 = this symptom did not occur during the last week
a = this symptom occurred at a slight intensity during the last week
b = this symptom occurred at a moderate intensity during the last week
c = this symptom occurred at a strong intensity during the last week

0 = this symptom did not occur during the last week
a = this symptom occurred at a slight intensity during the last week
b = this symptom occurred at a moderate intensity during the last week
c = this symptom occurred at a strong intensity during the last week

1. Fear whenever you are on a balcony/ bridge/ or the edge of a cliff......0 a b c
2. Feelings of sadness (gloom)..0 a b c
3. Choking sensations/ like the feeling of a “lump” in the throat ....0 a b c
4. Persistent feelings of fear without any reason......................................0 a b c
5. Frequent crying ....0 a b c
6. Feelings of fatigue and weakness in the morning that disappear during the day..0 a b c
7. Dissatisfactions with sexual life...0 a b c
8. Impressions that familiar things have .become unknown and strange..0 a b c
9. Vomiting in stressful situations ....0 a b c
10. Feelings of discomfort in large groups..0 a b c
11. Skin itching or rashes that disappear quickly.......................................0 a b c
12. Checking over and over whether everything is done correctly (the door locked, the oven turned off, and so on).................................0 a b c
13. Muscle cramps that always happen during certain activities – for example, fingers cramp during writing or playing music and so on .....0 a b c
14. Dizziness ....0 a b c
15. Lack of self-dependence..0 a b c
16. Feelings of annoying internal tensions..0 a b c
17. Discovering all kinds of serious diseases in yourself...........................0 a b c
18. Compulsive, bothersome thoughts, words/ or fantasies.......................0 a b c
19. Nightmares/ frightening dreams...0 a b c
20. Strong heartbeats (palpitations) without any physical activity ....0 a b c
21. Fear and other unpleasant sensations whenever staying alone, for example in an empty room and so on...0 a b c
22. Feelings of guilt/ blaming yourself..0 a b c
23. Loss of sensitivity in parts of the body ....0 a b c
24. Petrifying unexplainable fear that stops you from any kind of intensive experiencing of any unpleasant events.............................0 a b c
25. Very intensive experiencing of any unpleasant events.........................0 a b c
26. Problems with memory (getting worse)..0 a b c
27. Difficulties in sexual life because of – for example, tension of muscles in women or early ejaculation in men, and so on...............0 a b c
28. Feeling as if the world is in a fog...0 a b c
29. Persistent headaches...0 a b c
30. Strongly bothered by feelings that you have no one really close to you0 a b c
31. Wind (flatulence), or involuntary passing of gas.................................0 a b c
32. Frequently repeating the same acts that seem strange or unnecessary..0 a b c
33. Stuttering or stammering...0 a b c
34. Feeling flushes of blood into the head..0 a b c
35. Annoying lack of self-confidence ....0 a b c

0 = this symptom did not occur during the last week
a = this symptom occurred at a slight intensity during the last week
b = this symptom occurred at a moderate intensity during the last week
c = this symptom occurred at a strong intensity during the last week

36. Losses of attention that interrupt your activity....................................0 a b c
37. Performing ritualistic actions to try to avoid disease...........................0 a b c
38. Persistently fighting with thoughts of hurting or insulting someone ....0 a b c
39. Difficulties in falling asleep ....0 a b c
40. Heart pain ....0 a b c
41. Fear whenever in a car, train, .bus, or so on..0 a b c
42. Lack of self-confidence ....0 a b c
43. Temporary (periodic) paralyses of legs or hands ....0 a b c
44. Attacks of panic..0 a b c
45. Experiencing emotions strongly and deeply ....0 a b c
46. Feeling that your thinking is slower and not as clear as usual ....0 a b c
47. Aversions to sexual contacts with persons of the opposite sex ....0 a b c
48. Feeling that the world is unreal ....0 a b c
49. Dryness of the mouth ....0 a b c
50. Avoiding people, even those close to you ....0 a b c
51. Fainting...0 a b c
52. Strong internal desires to do useless things – for example, washing hands constantly and so on ....0 a b c
53. Sudden involuntary movements (tics)..0 a b c
54. Loss of appetite ....0 a b c
55. Being helpless in life ....0 a b c
56. Nervousness (restlessness) in performing that decreases your effectiveness ....0 a b c
57. Pertinent concerns over body functions – for example, heart-beats, pulse, digestion, and so on ....0 a b c
58. Obsessive: immoral thoughts ....0 a b c
59. Attacks of hunger – for example, the necessity to eat at night.............0 a b c
60. Feelings of heat or (and) cold without reasons ....0 a b c
61. Fears whenever you are in open places – for example, in large square, field, and so on ....0 a b c
62. Desire to take your life (suicidal thoughts) ....0 a b c
63. Periodic blindness or deafness ....0 a b c
64. Apprehensiveness...0 a b c
65. Inability to control your emotions despite the consequences...............0 a b c

0 = this symptom did not occur during the last week
a = this symptom occurred at a slight intensity during the last week
b = this symptom occurred at a moderate intensity during the last week
c = this symptom occurred at a strong intensity during the last week

66. Difficulty in concentration .... 0 a b c
67. Decrease or lack of sexual desire .... 0 a b c
68. Feelings of strangeness of one's own body .... 0 a b c
69. Diarrhoea .... 0 a b c
70. Shyness and embarrassment with persons of the opposite sex .... 0 a b c
71. Fears or other unpleasant sensations that appear only in locked (closed) spaces .... 0 a b c
72. Apathy – showing down of activity and thinking .... 0 a b c
73. Aphonia – inability to speak that suddenly appears and suddenly disappears .... 0 a b c
74. Constipation .... 0 a b c
75. Feelings of being worse than other people .... 0 a b c
76. Destroying things when you are angry or upset .... 0 a b c
77. Fears about one's own health and about contracting serious diseases .... 0 a b c
78. Persistent obsessive counting – for example, pedestrians, cars, lights, and so on .... 0 a b c
79. Frequently waking up during sleep .... 0 a b c
80. Reddening (blushing) on the face, neck, or chest .... 0 a b c
81. Fears when in crowds .... 0 a b c
82. Pessimism, expecting failure or disaster in the future .... 0 a b c
83. Faintness in difficult or unpleasant situations .... 0 a b c
84. Feelings of being threatened – without any reason .... 0 a b c
85. Unexpected strong feelings of happiness, joy, ecstasy .... 0 a b c
86. Constant fatigue .... 0 a b c
87. Unpleasant feelings connected with masturbation .... 0 a b c
88. Feelings that you are living as if in a dream .... 0 a b c
89. Trembling of legs, hands, or whole body .... 0 a b c
90. Feeling that people influence you easily .... 0 a b c
91. Allergic symptoms – colds, hay fevers, swellings and so on .... 0 a b c
92. Internal pressure to perform acts very slowly and exactly .... 0 a b c
93. Muscle cramps in different parts of the body .... 0 a b c
94. Excessive saliva in the mouth .... 0 a b c
95. Losing yourself in daydreams .... 0 a b c
96. Attacks of anger, hostility, that you cannot control .... 0 a b c

0 = this symptom did not occur during the last week
a = this symptom occurred at a slight intensity during the last week
b = this symptom occurred at a moderate intensity during the last week
c = this symptom occurred at a strong intensity during the last week

97. Feelings of haying serious diseases that threaten your life ....0 a b c
98. Excessive thirst..0 a b c
99. Insomnia ....0 a b c
100. Feelings of chill or heat without reason ....0 a b c
101. Fears of contact with things, animals, or places that are not dangerous ....0 a b c
102. Lack of energy and strength in any kind of activity.............................0 a b c
103. Difficulties in breathing – for example, breathlessness that appears and disappears suddenly...0 a b c
104. Feelings of apprehension (dread) before meetings, and so on ....0 a b c
105. Feeling that people do not think highly of you ....0 a b c
106. A lowering in the speed of thinking and perceiving.............................0 a b c
107. Pains or other disorders in the sexual organs ....0 a b c
108. Impressions that you have seen something before when you really have seen it for the first time ....0 a b c
109. Unpleasant feelings or pains under the influence of noise, bright light, delicate touch..0 a b c
110. Feelings that people do not like you (are prejudiced against you)........0 a b c
111. Involuntary passing of urine, for example during sleep.......................0 a b c
112. Excessive drinking of alcohol ....0 a b c
113. Trembling of ‘the face, eyelids, head, or other parts of the body..........0 a b c
114. Excessive perspiration in stress situation ....0 a b c
115. Feelings of being under the influence of the environment...................0 a b c
116. Persistent feelings of anger and hostility..0 a b c
117. Undefined “travelling” pains ....0 a b c
118. Feelings of rebelliousness ....0 a b c
119. Sleepiness during the day that forces you to fall asleep for a while, despite the situation...0 a b c
120. Flushing (a rush of blood) to your head ....0 a b c
121. Fears about the safety of close relatives that are not in any danger......0 a b c
122. Feelings of inferiority when compared to other people ....0 a b c
123. Disorders of balance...0 a b c
124. Fears of doing something terrible or of something terrible happening ....0 a b c
125. Feelings that people do not care about you and your problems...........0 a b c
126. Pressure (floods) of thoughts...0 a b c

0 = this symptom did not occur during the last week
a = this symptom occurred at a slight intensity during the last week
b = this symptom occurred at a moderate intensity during the last week
c = this symptom occurred at a strong intensity during the last week

127. Menstrual disorders in women .... 0 a b c
128. Feeling low intensities of emotions .... 0 a b c
129. Feelings of muscle tensions .... 0 a b c
130. Need to be alone .... 0 a b c
131. Heartburn .... 0 a b c
132. Passing urine frequently .... 0 a b c
133. Cramps (spasms) that force you to turn your head .... 0 a b c
134. Muscle pains – for example, in. The back, chest, and so on .... 0 a b c
135. Buzzing in the ears .... 0 a b c
136. Nausea .... 0 a b c
137. Decrease in sex drive .... 0 a b c
138. Deja vu .... 0 a b c
